# Molecular Recognition Affords Ligand-Free Au Nanoparticle Catalysts on an Interfacially Inert Supramolecular Support

**DOI:** 10.1021/acs.chemmater.6c01997

**Published:** 2026-09-28

**Authors:** Kushaan Bahl, Gerald Siu Hang Poon Ho, Brandon C. Bukowski, Marcella Lusardi

**Affiliations:** † Department of Chemical and Biological Engineering, Princeton University, Princeton, New Jersey 08544, United States; ‡ Princeton Materials Institute, Princeton University, Princeton, New Jersey 08544, United States; § Department of Chemical and Biomolecular Engineering, 1466 Johns Hopkins University, Baltimore, Maryland 21218, United States

## Abstract

Decoupling support and ligand contributions in supported metal nanoparticle (NP) catalysts remains an open challenge, and the challenge is synthetic: what makes a support interface inert is also what limits its ability to bind and disperse NPs, requiring ligands that are themselves active participants in the catalytic cycle. Here, we address this challenge using melamine cyanurate (MCA), whose supramolecular construction exposes coordinatively saturated terminations of melamine and cyanuric acid (CA) subunits that are interfacially inert yet accessible for functionalization using molecular recognition. Under mild aqueous conditions, a Au-bound thiol-functionalized CA derivative uniformly disperses onto the edges of MCA sheets via complementary hydrogen-bond donor–acceptor pairs, as shown by X-ray photoelectron spectroscopy (XPS), transmission electron microscopy (TEM), and density functional theory (DFT). Thermal treatment yields 2.5 ± 0.5 nm, monodisperse (dispersity index = 1.1), ligand-free Au^0^ NPs. MCA’s inertness at both NP-support and reactant-pool-support interfaces is established by XPS and the Au-catalyzed aerobic oxidative dehydrogenation of *N*-methyl-4-piperidinone, respectively, and benchmarked against conventional supports (graphite, silica, P25 TiO_2_), with and without capping ligands. The interfacial inertness of MCA, together with the bare, uniform NPs afforded by the recognition-based synthesis, enables quantitative decoupling of support and ligand contributions to reactivity, revealing that carbonaceous deposit formation and overoxidation are direct consequences of support functionality, rather than intrinsic to the Au^0^ active site. Free of both contributions, Au NPs on MCA achieve 100% selectivity to the target enaminone and 90% yield, versus <70% on conventional supported catalysts. More broadly, by resolving the usual trade-off between support inertness and ligand-free NP functionalization, this strategy, rooted in supramolecular principles, expands the design space of noninterfering supports for metal NP catalysts.

## Graphical abstract

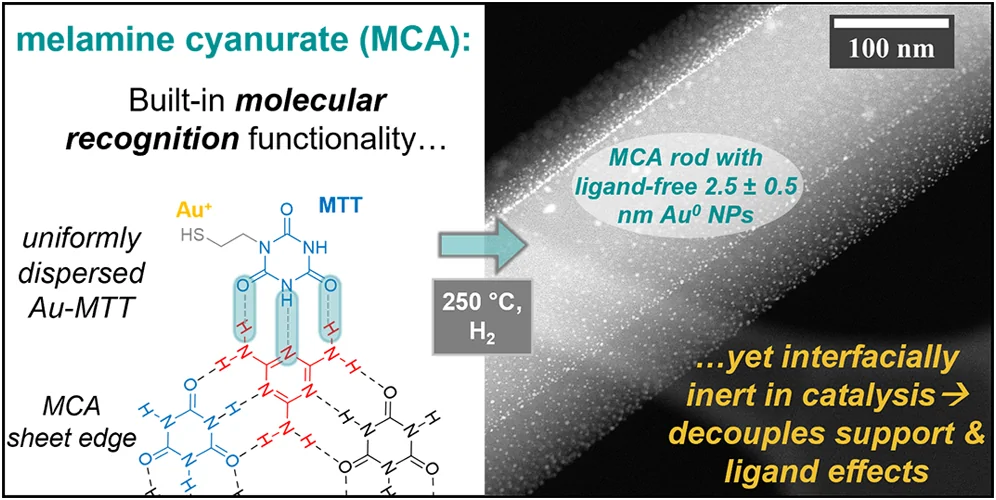

## 1 Introduction

Supported metallic nanoparticles (NPs) are widely deployed in oxidation, hydrogenation, and C–C coupling catalysis,^1–8^ and in these applications the support often plays a governing role in the catalytic cycle. Supports can modulate the electronic state of the NP through electronic metal–support interactions (EMSIs), as well as directly interact with catalytic intermediates (e.g., via acid–base interactions, redox cycles), tuning reaction rates and mechanisms, and by extension, turnover and selectivity.^9–19^ While such interactions are prevalent on many oxides (e.g., TiO_2_) and highly functionalized carbons, nonreactive supports such as graphite exhibit minimal interactions at both the NP-support and reactant-pool-support interfaces.^9,20^ This inertness is itself a valuable design handle. Inert supports can improve product selectivity, especially for reactive substrates, by limiting interactions with catalytic intermediates that promote undesired side pathways.^20–24^ They also provide closer approximations to the intrinsic reactivity of the NP surface, isolated from support contributions, enabling structure–function insights specific to the NP that inform the interpretation and design of NP-support systems more broadly.^20,25,26^

However, functionalizing inert supports with uniform, dispersed NPs remains a fundamental synthetic challenge: the same low surface functionality that makes a support inert also limits the anchoring and dispersion of metal precursors and the resulting NPs. Consequently, NP deposition on graphite, a prototypical nonreactive support, typically relies on capping ligands (e.g., poly­(vinyl alcohol), PVA) as mediators of both size control and dispersion.^23,24^ Yet, ligands undermine the central advantages of an inert platform; similar to reactive supports, they can participate in the catalytic cycle through strong steric and electronic contributions that obscure the intrinsic reactivity of the NP surface.^14,27–32^ Indeed, pronounced ligand effects on activity and selectivity have been reported across diverse reactions, including in selective oxidation routes involving hydroxymethyl furfural,^28^ glycerol,^31,32^ and propanediol substrates.^14^ While postsynthetic ligand removal is possible, the intensive conditions required (e.g., high calcination temperatures, solvent reflux) tend to drive NP aggregation and polydispersity, reducing active metal surface area and sacrificing the uniformity required for fundamental studies.^33–35^ Altogether, this highlights a trade-off in supported metal NP catalyst design, in which supports either participate in the catalytic cycle or require ligands that do. Thus, a strategy for achieving small, monodisperse, ligand-free NPs on a support that is inert at both the NP-support and reactant-pool-support interfaces remains an outstanding challenge.

Here, we introduce a synthetic strategy to break this trade-off based on melamine cyanurate (MCA, Figure 1a,b), the robust supramolecular assembly of melamine and cyanuric acid. MCA’s potential as an inert support is rooted in its supramolecular nature. In conventional supports, surfaces terminate by truncating the bulk coordination, leaving undercoordinated sites at the surface whose distinct electronic structure gives rise to EMSIs and reactant binding;^36^ in MCA, covalent bonding is fully saturated within each molecular subunit, and the lattice is held together by noncovalent interactions (hydrogen bonds, π–π stacking).^37^ Surface terminations therefore expose intact, coordinatively saturated molecules rather than undercoordinated sites, removing the electronic states that underpin EMSIs and reactant-pool interactions. At the same time, this supramolecular construction endows these surface terminations with inherent molecular recognition functionality arising from exposed melamine and cyanuric acid subunits. In this way, MCA decouples NP anchoring functionality from interfacial reactivity. We exploit this duality to prepare ligand-free NP catalysts, demonstrated here with Au, on MCA using 1-(2-mercaptoethyl)-1,3,5-triazinane-2,4,6-trione (MTT, Figure 1c), a mercaptoalkyl-functionalized cyanuric acid derivative. Au binds to the thiol of MTT, while MTT’s cyanuric acid backbone integrates into recognition sites at MCA sheet edges, yielding uniformly dispersed Au-MTT complexes. We show that these recognition sites, which are absent on graphite, whose ideal surfaces offer only nonspecific π–π stacking, are essential for uniform dispersion. Upon thermal treatment, Au is liberated from MTT and nucleates ligand-free Au^0^ NPs anchored at the sheet edges. The resulting 2.5 nm NPs exhibit narrow size distributions (dispersity near unity) matching those of colloidal synthesis methods,^38–41^ yet involve neither surface-passivating ligands nor a reactive support interface.

**Figure 1:**
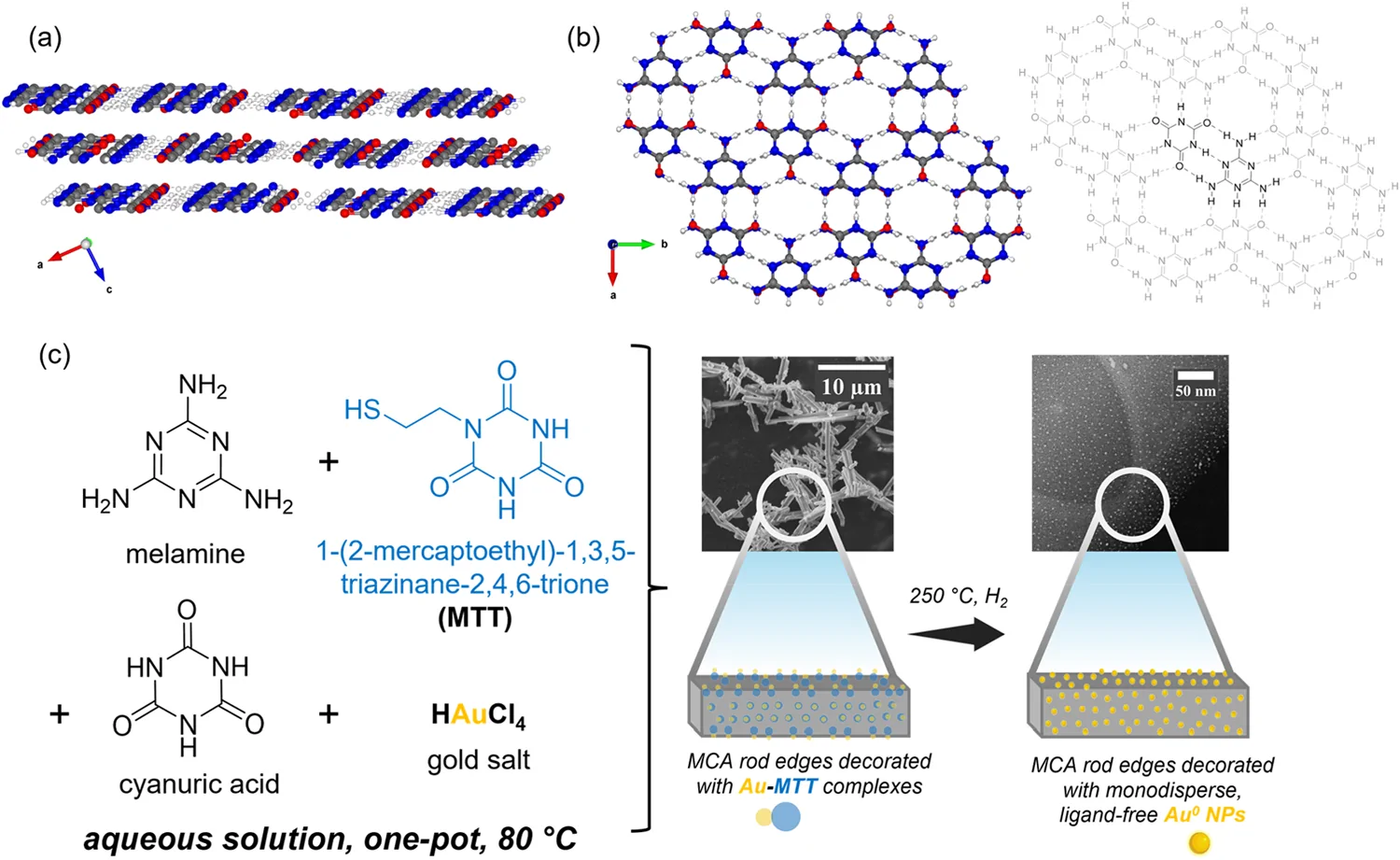
(a) Side and (b) top views of MCA’s layered structure showing hydrogen-bonded sheets that stack via π–π interactions. (c) Schematic of the MTT bridge synthesis method designed to functionalize MCA with monodisperse, ligand-free metal (metal = Au) NPs using molecular recognition between metal-MTT precursors and the MCA support.

We validated this design in the Au-catalyzed aerobic oxidative dehydrogenation of *N*-methyl-4-piperidinone (MP) to 1-methyl-2,3-dihydropyridinone (MDHP). Since both MP and MDHP are reactive substrates susceptible to side routes (e.g., sequential dehydrogenation), selectivity profiles provide a mechanistically informative probe of the support and ligand contributions to the catalytic cycle. Our results verify the inertness of MCA at both the NP-support and reactant-pool-support interfaces through interfacial X-ray photoelectron spectroscopy (XPS) before and after catalysis and through substrate adsorption and serial oxidation controls. These studies are benchmarked against conventional support materials (graphite, silica, and P25 TiO_2_), prepared with and without PVA-capping ligands, which span different modes of support inertness. Altogether, these studies reveal that the selectivity-limiting carbonaceous deposit formation and secondary oxidation of MDHP under these conditions are attributable to direct reactant- and product-support interactions rather than inherent to Au^0^, and that PVA ligands retard the desired first dehydrogenation step but do not influence selectivity significantly. In the absence of support and ligand contributions, Au NPs on MCA enable 100% MDHP selectivity at all conversions and result in the highest yield (90% vs <70% on conventional supported catalysts). These performance advantages, together with the quantitative decoupling of intrinsic NP, ligand, and support contributions to reactivity, are a direct consequence of supramolecular support surfaces and the synthetic strategy that exploits their mild yet accessible functionality.

## 2 Experimental Section

### 2.1 Reagents

All reagents were purchased from Sigma-Aldrich and used as-received: melamine (99%), cyanuric acid (98%), sodium hydroxide (98%), benzene (98%), 2-amino 2-thiazoline hydrochloric acid (98%), ethoxycarbonyl isothiocyanate (98%), dimethylformamide (99%), dimethyl sulfoxide (DMSO)-d6 (99.9%), gold­(III) chloride trihydrate (49.0% Au basis), chloroplatinic acid hexahydrate (37.5 wt % Pt basis), graphite (extra pure fine powder), silica gel (high purity grade, 60 Å, 200–400 mesh), 3-mercaptopropyl trimethoxysilane (MPTMS, 95%), (poly)­vinyl alcohol (80% hydration), sodium borohydride (NaBH_4_, 98%), ethanol (denatured with 1% methyl ethyl ketone), *N*-methyl-4-piperidinone (MP, 99%), and *N*-methyl pyrrolidone (NMP, 98%), toluene (99.5%), *n*-hexane (95%), acetone (99.5%), hydrochloric acid (37.5 wt %), sulfuric acid (95–98 wt %), nitric acid (70 wt %), ammonium hydroxide (28–30% NH_3_ basis), TraceCERT Au standard for ICP (997 mg/L standard), TraceCERT sulfur standard for ICP (1 g/L standard), and TraceCERT Pt standard for ICP (1 g/L standard). P25 TiO_2_ was kindly provided by Evonik. Ultrapure water with 18.2 MΩ·cm resistivity was delivered by a Milli-Q IQ7000 system. Gaseous oxygen (industrial grade), nitrogen (ultrahigh purity), argon (ultrahigh purity), and hydrogen (ultrahigh purity) were purchased from Airgas.

### 2.2 Catalyst Synthesis

All supported metal NP samples were prepared with a target loading of 1 wt % metal NP. For alkylthiol-Au functionalization on MCA and silica, 1.5 mol S/mol Au ratios were used for both supports.

#### 2.2.1 Synthesis of MCA-as

To a solution of 409 mg of cyanuric acid and 400 mL of DI water at 80 °C, 400 mg of melamine was added under vigorous mixing. The white precipitate (MCA) immediately formed, and the solution was stirred for an additional 0.5 h. The precipitate was either vacuum-filtered, washed with 100 mL of DI water, and dried in air at 30 °C overnight to generate MCA-as; or, in one-pot, further functionalized by MTT and Au (see below).

#### 2.2.2 Synthesis of Supported Au NPs by Traditional Sol Immobilization

Supported Au NPs were prepared by the sol immobilization method using PVA as a ligand following the procedure outlined by Hutchings et al.^24^ Samples prepared using MCA and graphite supports are denoted as Au-PVA-MCA and Au-PVA-G, respectively. A calcined Au-PVA-G sample was prepared using a 2 °C/min ramp to 450 °C, held at 450 °C for 4 h, in air.

#### 2.2.3 Synthesis of Supported Au NPs by Traditional Deposition-Reduction or Deposition-Precipitation

Supported Au NPs were prepared by deposition of the gold salt, with or without subsequent reduction with NaBH_4_, on MCA or graphite. 500 mg of the support was dispersed in 400 mL of DI water at room temperature. To this solution, 5.0 mL of a 5.1 mM HAuCl_4_ solution was added, and the mixture was stirred for 1 h. Then, for reduction by NaBH_4_, 3 mL of an aqueous solution of sodium borohydride (0.1 M) was added to the mixture dropwise. After stirring for 1 h, the precipitate was filtered and washed with 100 mL of DI water and dried at room temperature overnight. For deposition-precipitation preparation of Au NPs on TiO_2_, 400 mg of P25 TiO_2_ was dispersed in 200 mL of DI water at room temperature under stirring. A 1 wt % ammonium hydroxide solution was prepared and added dropwise (approximately 0.5 mL) to the solution to bring the mixture to pH 8. To this, a 4 mL of a 5.1 mM HAuCl_4_ solution was added dropwise and the pH was monitored, with more 1 wt % ammonium hydroxide solution added dropwise (approximately 0.2 mL) until the mixture reached pH 9. After stirring for 1 h, the solid was separated by centrifugation (10,000 rpm, 5 min, with an additional 100 mL of DI water wash) and dried at 80 °C overnight. The recovered material was then calcined in 100 sccm air (ramped at 2 °C/min to 400 °C, held at 400 °C for 4 h) to yield Au–P25 TiO_2_.

#### 2.2.4 Synthesis of Supported Au NPs on SiO_2_ by MPTMS

1 g of vacuum-dried SiO_2_ was dispersed in 40 mL of anhydrous toluene under reflux. A separate solution was prepared containing 15.5 mg MPTMS in 1 mL anhydrous toluene. Under vigorous stirring, this solution was added dropwise to the SiO_2_-containing solution, and stirring continued under reflux for 15 h. The mixture was vacuum-filtered, washed with 100 mL of toluene, hexanes, and ethanol, respectively, then dried at 80 °C for 12 h to yield MPTMS-SiO_2_. For gold functionalization, 800 mg MPTMS-SiO_2_ was dispersed in 400 mL of DI water at room temperature and 8.2 mL of an aqueous solution of HAuCl_4_ (5.1 mM) was added dropwise under stirring. The sample was then filtered, washed with 100 mL of water, and dried at 80 °C for 12 h to yield Au-MPTMS-SiO_2_-as (as-synthesized). To generate supported NPs, this sample was calcined in 100 sccm air (ramped at 2 °C/min to 550 °C, held at 550 °C for 4 h) to generate Au-MPTMS-SiO_2_.

#### 2.2.5 Synthesis of MTT

MTT was synthesized following the procedure described by Klayman et al.^42^ Briefly, a 10 mL of a 1.4 M solution of ethoxycarbonyl isothiocyanate in benzene was added to a solution of 2-amino-2-thiazoline hydrochloric acid (10 mmol) dispersed in 10 mL water and 40 mL of benzene under vigorous stirring. 10 mL of 1 M NaOH was added, and the mixture was stirred for 15 min, after which another 10 mL of 1 M NaOH was added, and the mixture was left to stir for 1 h. The aqueous layer was collected, and the benzene solution was extracted with 10 mL of 1 M NaOH. 3 M H_2_SO_4_ was added dropwise to the resultant aqueous phase until a pH of 3 was reached and a precipitate was formed. The precipitate was filtered, washed with 100 mL of DI water, and dissolved in a solution of warm DMF at 80 °C (1 g of precipitate in 10 mL of DMF). To this solution, water was added dropwise until a precipitate formed, and the mixture was allowed to cool; the recrystallized product was vacuum-filtered. 1 g of the recrystallized product was dispersed in 50 mL of 18% HCl and heated to reflux under Ar atmosphere for 6 h. The mixture was cooled, precipitating white crystals that were filtered and washed with 100 mL of water to yield MTT. ^1^H NMR confirmed the structure (Figure S1).

#### 2.2.6 Synthesis of Supported NPs by the MTT Method

10 mL of a 6.6 mM MTT in ethanol solution was prepared at 80 °C and diluted in 60 mL of DI water at the same temperature. This solution was added dropwise to the MCA synthesis solution (80 °C, 800 mg of MCA) under vigorous stirring. After stirring for 1 h, the solution was allowed to cool, and 8.2 mL of an aqueous solution of HAuCl_4_ (5.1 mM) was added dropwise under stirring. After stirring for an additional 1 h, the solid was vacuum-filtered, washed with 100 mL of DI water, and dried in air at 30 °C overnight. The sample is denoted as Au-MTT-MCA-as (as-synthesized). To generate supported Au NPs, the -as form was heated in a tube furnace under H_2_ flow (200 sccm) with a ramp program of 2.3 °C per min to 250 °C, 6 h hold at 250 °C, to produce Au-MTT-MCA. Control samples were prepared in an identical fashion using graphite as a support (Au-MTT-G-as, Au-MTT-G upon thermal treatment in hydrogen). Supported Pt NPs were prepared in the same way, but using H_2_PtCl_6_ instead of HAuCl_4_ and a thermal treatment at 270 °C in H_2_ instead of 250 °C (Pt-MTT-MCA-as, Pt-MTT-MCA after thermal treatment).

### 2.3 Characterization

The ^1^H nuclear magnetic resonance (NMR) spectrum of MTT was collected on a Bruker Avance III 500 MHz spectrometer in DMSO-d6 solvent. Powder X-ray diffraction (XRD) patterns were collected on a Bruker D8 Discover X-ray diffractometer with Cu Kα radiation (λ = 1.54 Å) at a scan rate of 10° per minute with a step size of 0.05°. Scanning electron microscopy (SEM) was performed using a FEI Quanta 200 scanning electron microscope equipped with a Schottky field emission gun (FEG) with an accelerating voltage of 5 kV. Transmission electron microscopy (TEM) and high-angle annular dark-field (HAADF) scanning TEM (STEM) were conducted on a Talos F200X instrument equipped with a high-brightness FEG, a SuperX detector for energy-dispersive X-ray spectroscopy (EDS), and a HAADF detector. Samples were prepared by drop-casting sonicated suspensions of a given sample in ethanol onto carbon-coated Cu grids (CF200-Cu-50, Electron Microscopy Sciences). The maximum magnification on MCA samples was limited to 200kX and 75kX on TEM and HAADF-STEM, respectively, due to the instability of MCA under higher magnification.^43,44^ Aberration-corrected HAADF-STEM on selected samples was conducted on a Titan Cubed Themis 300 double Cs-corrected scanning/transmission electron microscope equipped with a Schottky X-FEG high-brightness electron source with 0.07 nm (TEM), 0.06 nm (STEM) information limit and a double Cs-corrector DCOR (Cs image + DCOR probe corrector). These samples were prepared in the same fashion as above but using lacey carbon-coated Cu grids (01824, Ted Pella). Particle size distributions are reported using measurements from ImageJ on at least 200 particles. In addition to reporting the average particle size with one standard deviation, the PSDs also report the surface-averaged NP diameters from the TEM measurements, ⟨*d*_TEM_⟩, where *n*_*i*_ is the number of NPs with a given diameter *d*_*i*_ (eq 1), and dispersity index (DI) values (via eq 2).^45^

1 $$\left\langle d_{TEM}\right\rangle =\frac{\sum n_{i}d_{i}^{3}}{\sum n_{i}d_{i}^{2}}$$

2 $$DI=\frac{\frac{\sum n_{i}d_{i}^{3}}{\sum n_{i}d_{i}^{2}}}{\frac{\sum n_{i}d_{i}}{\sum n_{i}}}$$

X-ray photoelectron survey spectra as well as high-resolution spectra were collected on a Thermo Scientific K-α+ spectrometer with an Al Kα source and electron flood gun. A thin layer of powdered sample was deposited onto carbon tape, and all binding energies were corrected by the C 1s peak at 284.8 eV. At least two spectra were collected per sample to ensure reproducibility (defined by ≤0.1 eV binding energy difference). High-resolution XPS spectra were fit using a Voigt function in the Thermo Scientific Avantage software, with all fitted peaks in a given spectrum constrained to the same degree of Gaussian/Lorentzian.^46^ For nitrogen environments in MCA, we fit the N 1s signal with three peaks of equal area and full width at half-maximum (FWHM) values to account for the three primary chemical environments of N in MCA (3 endocyclic N in cyanuric acid, 3 endocyclic N in melamine, and 3 exocyclic N in melamine).^47,48^ In the O 1s region, we fit the spectrum for MCA with one peak corresponding to the O in cyanuric acid (532.0 eV). Sulfur peaks were fit using two S 2p doublets due to spin–orbit splitting, corresponding to 2p 3/2 and 2p 1/2 in a 2:1 ratio, respectively.^49^ Au 4f peaks were fit using two doublets due to spin–orbit splitting corresponding to 4f 7/2 and 4f 5/2 in a 4:3 ratio, respectively. Pt 4f peaks were fit in a similar fashion but with an asymmetric correction factor applied.^50^

Inductively coupled plasma (ICP)-atomic emission spectroscopy experiments were conducted on an Agilent 5800 ICP-OES instrument. Prior to analysis, 20 mg of sample was digested in 12 mL aqua regia overnight and then diluted with 5 wt % aqueous HNO_3_ to between 0.1 and 2 ppm Au concentrations. A calibration curve was created with a 997 mg/L Au standard. Sulfur and platinum ICP quantification protocols followed a similar procedure. Au loadings on silica samples were measured by difference. After functionalization of MPTMS-SiO_2_ with Au, the powder was filtered and 25 mL of liquid filtrate was combined with 25 mL of 10 wt % aqueous HNO_3_. The amount of Au loaded onto the SiO_2_ was calculated from the difference between the filtrate concentration and the HAuCl_4_ amount added in solution for functionalization. The limit of quantification for S is defined by 10× the standard deviation at 0 concentration, extrapolated from triplicate runs on calibration standards. N_2_ adsorption–desorption isotherms were collected at 77 K on a Micromeritics 3Flex instrument. Prior to analysis, samples were outgassed at 100 °C for 2 h. The Brunauer–Emmett–Teller (BET) surface area was calculated from the adsorption isotherm in the region between 0.05 and 0.3 *P*/*P* ^0^. Thermogravimetric analysis (TGA) was conducted on a Mettler-Toledo TGA 2. 20 mg of the sample was loaded into an alumina crucible and heated from 25 to 800 °C with a 5 °C/min ramp rate under 20 sccm of air.

### 2.4 DFT

We used the Vienna Ab Initio Simulation Package (VASP) version 6.3 to perform density functional theory (DFT) calculations.^51,52^ The BEEF-vdW functional^53^ with dispersion-corrected generalized gradient approximation (GGA) as well as projector augmented wave (PAW) pseudopotentials were used for geometry optimization.^54,55^ All calculations were performed spin-polarized due to the presence of Au, and a dipole correction perpendicular to the surface was applied. A plane-wave cutoff of 475 eV, a force convergence criterion of 0.03 eV Å^–1^, and an energy convergence criterion of 1 × 10^–5^ eV were chosen. As MCA is a wide-bandgap molecular insulator, the electronic structure does not vary with *k*, and the Brillouin zone of all systems investigated in this work was sampled using a single Γ-centered *K*-point.

The melamine cyanurate (MCA) structure, belonging to the monoclinic *I*2/*m* space group, was obtained from the Cambridge Structural Database (CSD-QACSUI01),^56^ which was deposited based on the experimental structure reported by Prior et al.^57^ The lattice parameters reported by Prior et al. (*a* = 14.8152(19) Å, *b* = 9.6353(18) Å, *c* = 7.0405(9) Å) were relaxed to final lattice vectors *a* = 14.7456(70) Å, *b* = 9.7174(19) Å, *c* = 7.3457(25) Å. For calculations of potential energy at the reference state, melamine, cyanuric acid, Au-MTT, and their coordinated complexes were all optimized in an 18 × 18 × 18 Å cubic unit cell.

To create surface models, we used the Ogre Python package developed by Yang et al.^58^ to cleave slabs from the (001) MCA surface of the primitive unit cell while keeping surface molecules intact, thereby preventing unphysical fragmented melamine or cyanuric acid terminations. Slabs were constructed using the Atomic Simulation Environment (ASE)^59^ with 2 bilayers (4 layers, 432 atoms in total) and 25 Å of vacuum between images of the periodic slab in the *z*-direction. Two symmetry-inequivalent (001) terminations arise from the alternating layer stacking of bulk MCA. Slab models of both were constructed, with the second generated from the first by cyclic permutation of the layers, i.e., translating the topmost layer to the bottom of the slab to expose the alternate stacking registry at the surface. Both models present the natural terminations of melamine and cyanuric acid monomers (−N, −NH_2_, −N–H, −C=O) at the step edge, which represents the exposed sheet edges of MCA rods. These surfaces feature a lower and upper basal plane separated by the step edge, with both basal planes being ∼7.6 Å wide and ∼19.4 Å in the *y*-axis direction within the supercell. Considering the dimensions of the coordinating Au-MTT complex, 1 × 2 × 2 supercells were used to eliminate possible undesirable interactions between periodic images of coordinating ligands, but replications were kept to a minimum for computational efficiency. Static calculations on the two MCA structures found that their potential energies were within 0.03 eV of each other, indicating that the rearrangement results in structures that are almost thermally degenerate and could coexist in equilibrium. To elucidate favorable geometries of Au-MTT coordinated to MCA and probe molecular recognition, initial Au-MTT placements were chosen to resemble the molecular packing of bulk MCA and relaxed to the 0, 2, or 3 hydrogen-bond minima, resulting in six distinct configurations with varied combinations of hydrogen bonding and π–π stacking.

The adsorption energy of Au-MTT on MCA surfaces was calculated as shown in eq 3, where *E*_AuMTT/MCA_ is the potential energy of the Au-MTT complex bound to MCA, *E*_MCA_ is the potential energy of the bare MCA slab, *E*_AuMTT_(g) is the potential energy of Au-MTT in vacuum, and ΔZPE is the zero-point energy correction (eq 4). Zero-point energy (ZPE) corrections were obtained from a vibrational frequency calculation using an adsorbate-only Hessian approach. All MCA surface atoms were held fixed during the calculation, with only the Au-MTT adsorbate atoms displaced. The ZPE of the isolated gas-phase Au-MTT molecule was calculated, where the sum for ZPE runs over real vibrational frequency modes only. For vibrational frequency calculations, VASP settings are identical to the ones previously described for geometry optimization.

3 $$E_{ads}=E_{AuMTT/MCA}-E_{AuMTT}\left(g\right)-E_{MCA}+\Delta Z\mathrm{PE}$$

4 $$\Delta Z\mathrm{PE}={Z\mathrm{PE}}_{AuMTT/MCA}-{Z\mathrm{PE}}_{AuMTT}\left(g\right)$$

### 2.5 MCA Stability Testing

The stability of MCA as a support under different conditions representing catalytic environments was systematically tested. MCA-as was subjected to oxidizing (air) and reducing (100% H_2_) conditions at 250 °C under 200 sccm gas flow in an open alumina crucible, and refluxing in water (100 °C), ethanol (80 °C), and toluene (110 °C) under magnetic agitation (50 mg MCA/10 mL solvent). All treatments were conducted for 6 h. In the solution-phase experiments, solids were recovered by vacuum filtration.

### 2.6 Catalytic Reactivity of Supported Au NPs in the Aerobic Oxidative Dehydrogenation of *N*-Methyl-4-piperidinone

Catalysts were evaluated in the oxidative dehydrogenation of *N*-methyl-4-piperidinone (MP) to 1-methyl-2,3-dihydropyridinone (MDHP). To a 20 mL vial with a PTFE-septum lid, 2.0 mL of DI H_2_O, 0.1 mmol of MP, and 0.1 mmol of *N*-methyl pyrrolidone (NMP) as an internal standard were added. 50 mg of the catalyst was then added to the solution. The vial was sealed, and O_2_ at 100 sccm was flowed through the headspace for 5 min to create an O_2_ atmosphere at 1 atm before heating the vial to 60 °C, while stirring at 400 rpm. The reaction was allowed to proceed for a specified amount of time (between 5 min and 6 h) before cooling to room temperature. Prior to injection onto a gas chromatograph (Agilent 8890B) equipped with a CP-SIL-5 column, Polyarc, and flame ionization detector, 0.1 mL of the reaction mixture was passed through a Choice Hydrophilic PTFE 0.22 μm tip filter and diluted with 0.9 mL acetone. MP adsorption tests were conducted in an identical fashion to the reactions, but on bare (Au-free) supports. Tests with NMP (the internal standard) were conducted to confirm that it has no significant interactions (e.g., adsorption) onto the supports used.

For the MDHP serial oxidation test, standard reaction conditions were used with the Au-MTT-MCA catalyst, and the reaction was allowed to go to completion (18 h). 100% conversion of MP was measured by GC, and the reaction liquor was confirmed to contain only MDHP as the product by ^13^C NMR. This solution was vacuum-filtered to recover the reaction liquor and charged into a reaction vessel with fresh catalyst. This reaction proceeded as described above, except with 0.03 mmol of MDHP and 20 mg of catalyst.

Conversion of MP, product yield, and liquid-phase selectivity are reported on a mole basis: respectively, mmol MP converted per mmol MP charged; mmol MDHP or MHP produced per mmol MP charged; and MDHP produced per mmol MDHP and MHP produced, where MHP is the side product 1-methyl-4­(1H)-pyridinone. Site-time yield (STY, eq 5) is calculated based on the mmol of MDHP generated per mmol surface Au per hour (eq 6), as estimated from the dispersion *D* (equal to the fraction of metal atoms exposed on NP surfaces, eq 7), and total Au loading (measured by ICP = *m*_Au_/*m*_catalyst_)­

5 $$STY=\frac{mmol\,\;MDHP\;produced}{mmol{Au}_{surface}\cdot hr}$$

6 $$mmol{Au}_{surface}={\frac{D^{*}{m_{catalyst}}^{*}\left(m_{Au}/m_{catalyst}\right)}{{MW}_{Au}}}^{*}\left(1000\frac{mmol}{mol}\right)$$

7 $$D=6\frac{{\mathrm{v̅}}_{m}/{\mathrm{a̅}}_{m}}{\left\langle d_{TEM}\right\rangle }$$

where *v̅*_m_ (1.65 × 10^–2^ nm^3^) and *a̅*_m_ (8.75 × 10^–2^ nm^2^) are the effective bulk atomic density and effective area occupied by a metal atom on polycrystalline surfaces for Au, respectively; MW_Au_ is the molecular weight of Au; and *m*_catalyst_ is the mass of catalyst loaded into the reaction.^45^ This method is used to estimate surface Au since typical chemisorption methods for rigorous quantification of metal dispersion are not feasible for gold.^60–62^ Reactions were repeated a minimum of two times, and the average values are reported. Error bars reflect one standard deviation from the average. The carbon balance is >95% in all runs.

Selected samples were filtered and mixed in a 90:10% ratio with D_2_O for analysis by ^13^C NMR for product determination (Bruker Avance III 500 MHz spectrometer, 128 scans, 28.6 s recycle delay). Control studies were conducted in an identical fashion as described above using no catalyst (i.e., a blank); MCA-as without Au; and Au-MTT-MCA catalyst with Ar instead of O_2_, all for 6 h. For analyzing recyclability and the spent catalyst, the reaction was terminated after 6 h by cooling down to room temperature, after which the reaction mixture was vacuum-filtered. The retentate was washed with 50 mL of DI H_2_O to recover the spent catalyst. The catalyst was then dried at 80 °C before being redispersed into a fresh solution of the reaction mixture to proceed for another cycle. For quantifying leached Au, the filtrate was subjected to the same dilution scheme and ICP analysis as described above.

## 3 Results and Discussion

### 3.1 Characterization of the MCA Support and Its Stability

XRD (Figure S2) and high-resolution XPS (Figure S3) confirm phase-pure MCA with the expected hydrogen-bonded structure. MCA synthesized in aqueous solution adopts a rod-like morphology as indicated by SEM (Figure S4a), with the rod ends defined by hydrogen-bonded layers and the length of the rod formed from layer stacking.^63,64^ Nitrogen adsorption–desorption isotherms (Figure S5) show a nonporous material with a BET surface area <10 m^2^/g, similar to that of graphite. To validate MCA’s stability under working conditions, it was subjected to environments representative of catalyst processing and deployment, including oxidizing (air) and reducing (H_2_) at 250 °C, as well as refluxing solvents with varied polarity (water, ethanol, and toluene) under stirring. Analysis by XRD and SEM shows that, despite its supramolecular nature, MCA retains its long-range order and morphology across all treatments except toluene (Figures S4 and S6–S8; Table S2; detailed discussion in the Supporting Information (SI)). After toluene reflux, some rods shorten (from ∼5–10 μm to ∼1 μm), which we attribute to delamination by interruption of interlayer π–π stacking interactions (rather than intralayer hydrogen bonds), due to the lack of competitive hydrogen-bond donors/acceptors in nonpolar toluene;^65^ even so, long-range order is preserved, and >90 wt % of the MCA is recovered, indicating no significant dissolution. Moreover, N and O 1s environments in XPS are unperturbed by H_2_ or air at 250 °C (Figure S7, Table S1), consistent with the redox inertness anticipated from MCA’s saturated molecular coordination. Thus, while MCA’s noncovalent backbone restricts it to milder conditions than covalent supports, these results demonstrate that MCA withstands the processing and reaction environments typical of liquid-phase selective oxidation^22,23,66–68^ and hydrogenation^69^ catalysis.

### 3.2 Ligand-Free Metal NP Synthesis on MCA via Molecular Recognition

Leveraging MCA as a catalyst support requires techniques to functionalize it with dispersed, uniform metal NPs. On “active” supports such as TiO_2_, rich surface functionality enables simple deposition-precipitation or deposition-reduction methods.^70–73^ However, these deposition techniques are not transferable to MCA as they result in low Au uptake (Table S3) or very poor dispersion (Figure S9). Functionalizing MCA therefore requires a different strategy. An established method on silica supports uses bifunctional “bridge” molecules, e.g., mercaptopropyl silanes, in which the thiol binds the metal precursor while the silane covalently grafts to the silica surface.^74–77^ Molecular-level dispersion of the resulting metal–thiol complexes promotes uniform nucleation and growth environments upon thermal treatments that liberate the bridge, resulting in monodisperse, ligand-free metal NPs.^78,79^ We introduce MTT as a bridge molecule for MCA that maintains the thiol functionality for binding metal precursors but replaces covalent anchoring with molecular recognition, as we establish computationally in Section 3.2.2. We benchmark this approach against silica, using the established (3-mercaptopropyl)­trimethoxysilane (MPTMS) bridge, and against graphite, an inert support that shares MCA’s low surface functionality but lacks its molecular recognition sites. To demonstrate the generalizability of the molecular recognition synthesis strategy, we applied the same one-pot MTT protocol to Pt; analogous precursor coordination, dispersion, and NP formation were observed, with full characterization provided in the SI (Figure S10).

#### 3.2.1 MTT as a Bridge Molecule: Metal–Thiol Binding and Dispersion on MCA

To assess the efficacy of MTT in binding and dispersing Au precursors onto MCA, we evaluated Au-MTT-MCA-as using high-resolution XPS. Upon functionalization of MCA with MTT and the gold chloride salt in the one-pot method, XPS confirms the presence of S and Au species (Figure 2a,b, respectively). The 2p 3/2 and 2p 1/2 peaks at 162.9 and 164.1 eV, respectively, are attributed to S bound to Au.^80^ In the Au 4f region, two peaks manifest at 84.5 and 88.2 eV, corresponding to 4f 7/2 and 4f 5/2, respectively. These binding energies are consistent with the presence of Au in a uniform, oxidized state (Au^+^) bonded to a thiol. Au-MPTMS-SiO_2_-as was loaded with the same ratio of mol S:g support and mol S:mol Au as Au-MTT-MCA-as. In Au-MPTMS-SiO_2_-as_,_ both the S and Au environments have the same binding energies (within 0.2 eV) as Au-MTT-MCA-as, indicating similar Au–S binding.^75^ The silica sample also has a smaller, second population of sulfur species at 2p 3/2∼164 eV that can be assigned to the presence of free thiols.^80,81^ Elemental analysis by ICP shows nearly complete uptake of solution-phase Au onto the thiol-functionalized MCA and silica supports, corresponding to 0.97 and 1.0 wt % Au, respectively (Table 1). This ∼10× improvement in Au utilization compared to the MTT-free deposition (Table S3) demonstrates efficient, thiol-mediated Au binding on MCA.

**Figure 2:**
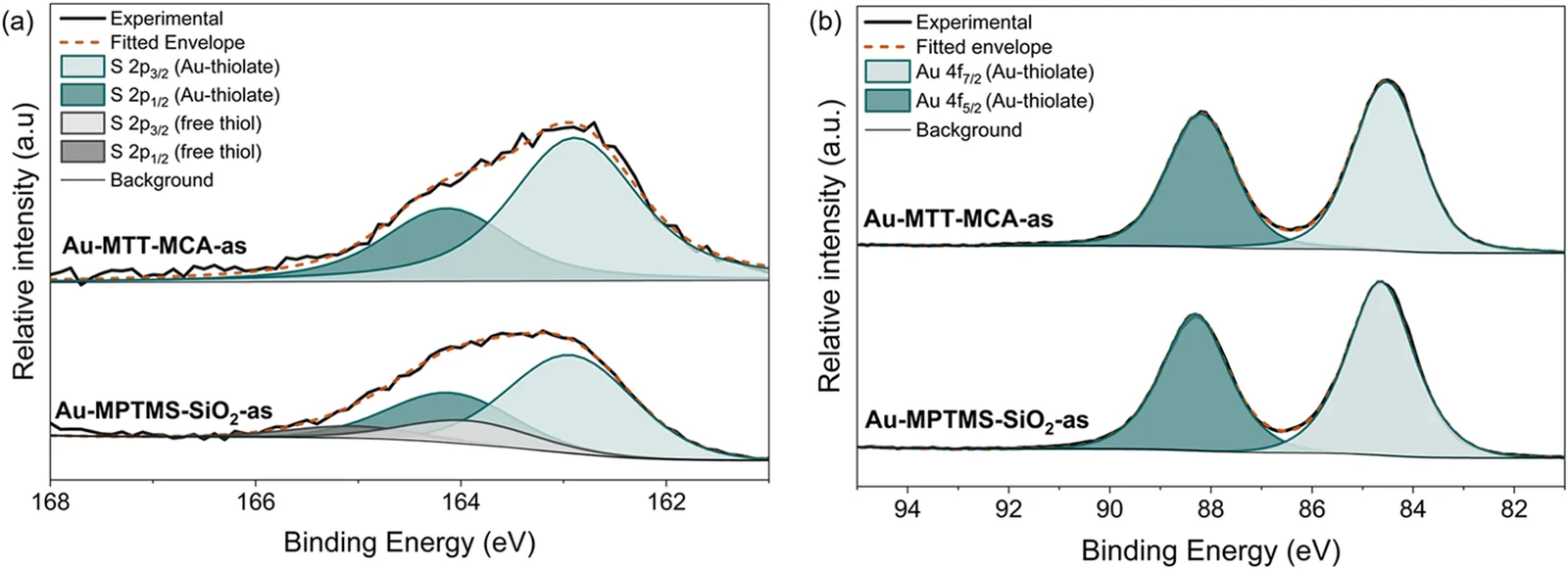
High-resolution (a) S 2p and (b) Au 4f XPS spectra of as-synthesized catalysts prepared using the thiolated bridge approach on MCA and silica supports.

**Table 1:**
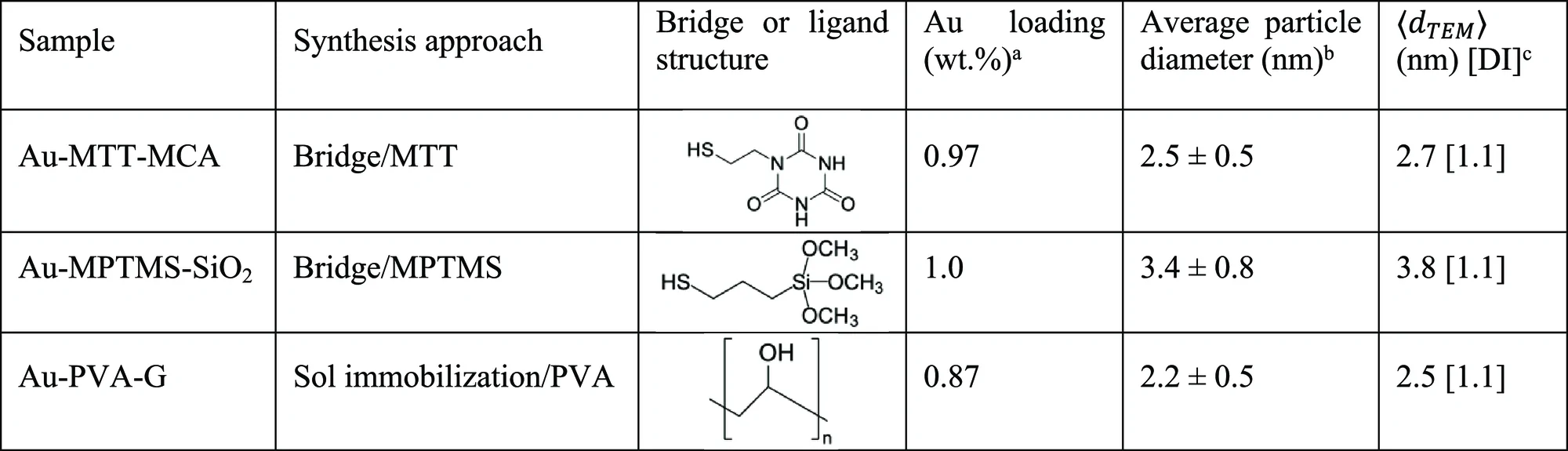
Summary of Synthesis Approaches, Au Loadings, and NP Particle Diameters of Au NPs Prepared on Various Supports

a Measured by ICP.

b Measured by TEM; ± reflects one standard deviation from the average.

c Calculated from TEM using eqs 1 and 2.

TEM analysis on Au-MTT-MCA-as (Figure 3a) shows no resolvable Au structures (e.g., nanoparticles, clusters). This is further evidenced by HAADF-STEM (Figure 3b) and elemental mapping (Figure 3c–g), which show uniform Au and S distributions indicative of rods highly decorated with S-bound Au species. Together, results from high-resolution XPS, TEM/STEM-EDS, and ICP suggest that MTT fulfills both functions of binding Au and dispersing it on the support surface. Analogous TEM and HAADF-STEM-EDS analysis of Au-MPTMS-SiO_2_-as likewise indicates high, thiol-mediated Au dispersion (Figure S11a–g). To probe whether the dispersion observed on Au-MTT-MCA-as requires the recognition sites of MCA, the synthesis was conducted with graphite in place of MCA. In stark contrast, significant Au–S coclusters are observed in the as-synthesized form (Au-MTT-G-as, Figure S12a–g). Since graphite offers no specific hydrogen-bond donor–acceptor sites that are complementary to MTT, Au-MTT complexes presumably bind nonspecifically via π–π stacking or at defect sites of heterogeneous distribution and strength. These results indicate that specificity in the bridge-support interaction is responsible for uniform dispersion of the metal-bridge complex, a prerequisite for monodisperse NP formation upon thermal treatment.

**Figure 3:**
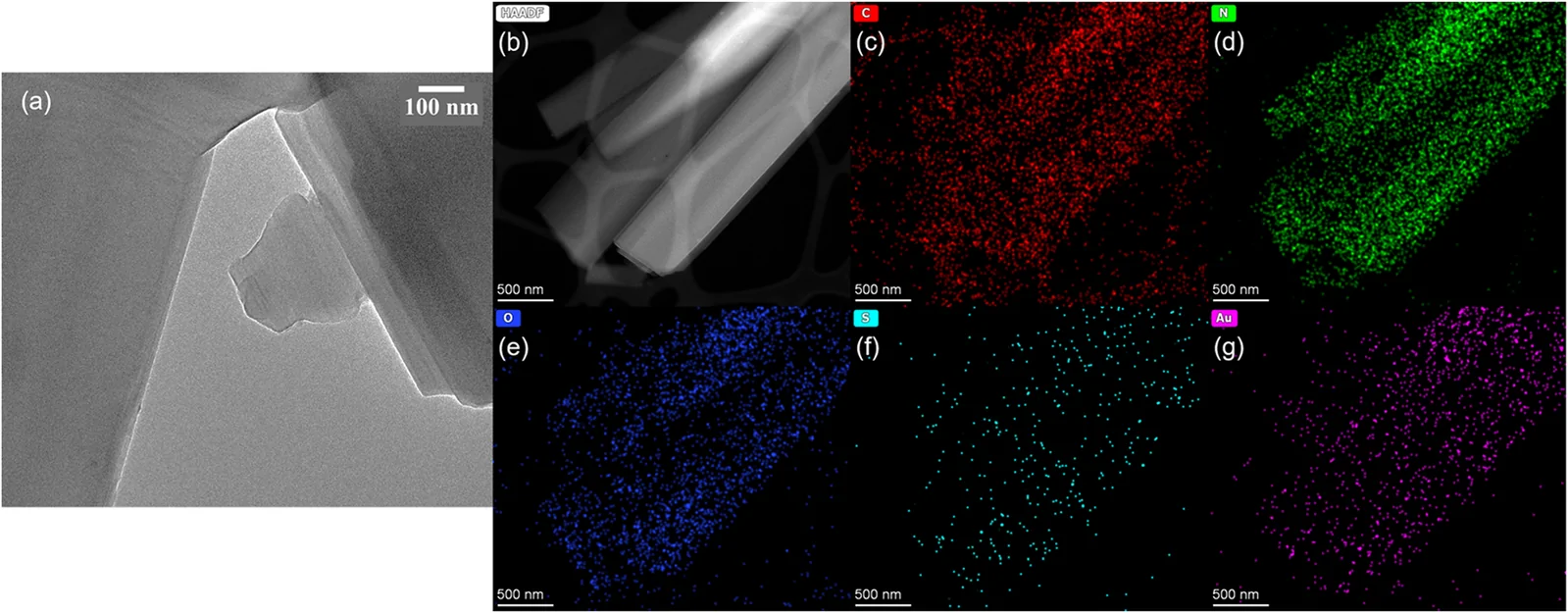
High-resolution (a) TEM and (b) HAADF-STEM with (c–g) EDS of Au-MTT-MCA-as.

#### 3.2.2 Molecular Basis for Efficient Au-MTT Dispersion: DFT Analysis of Au-MTT/MCA Interactions

To establish the role of molecular recognition in the functionalization of MCA by metal-MTT complexes, we calculated zero-point energy (ZPE)-corrected adsorption energies (*E*_ads_) of Au-MTT on edge and basal-plane sites of MCA. We hypothesized that Au-MTT interacts with MCA through a combination of in-plane hydrogen bonding between the cyanuric acid backbone of Au-MTT and donor–acceptor sites at sheet edges, and nonspecific, out-of-plane π–π stacking between triazine/triazinane rings. To differentiate hydrogen bonding from nonspecific noncovalent contributions, we chose an MCA surface with both an extended hydrogen-bonded sheet and a sheet edge (Figure S13). Geometry optimizations reveal that Au-MTT adsorbs to MCA through either 0, 2, or 3 hydrogen bonds. For a given number of hydrogen bonds (nH), we calculated the adsorption energies for two distinct configurations of Au-MTT relative to the MCA surface (A and B), generating a total of six representative configurations and adsorption energies (Figures S14–S16, Table S4). We focus the discussion here on the more energetically favorable configuration (nH-A) for a given number of hydrogen bonds, which is summarized in Figure 4a–c.

**Figure 4:**
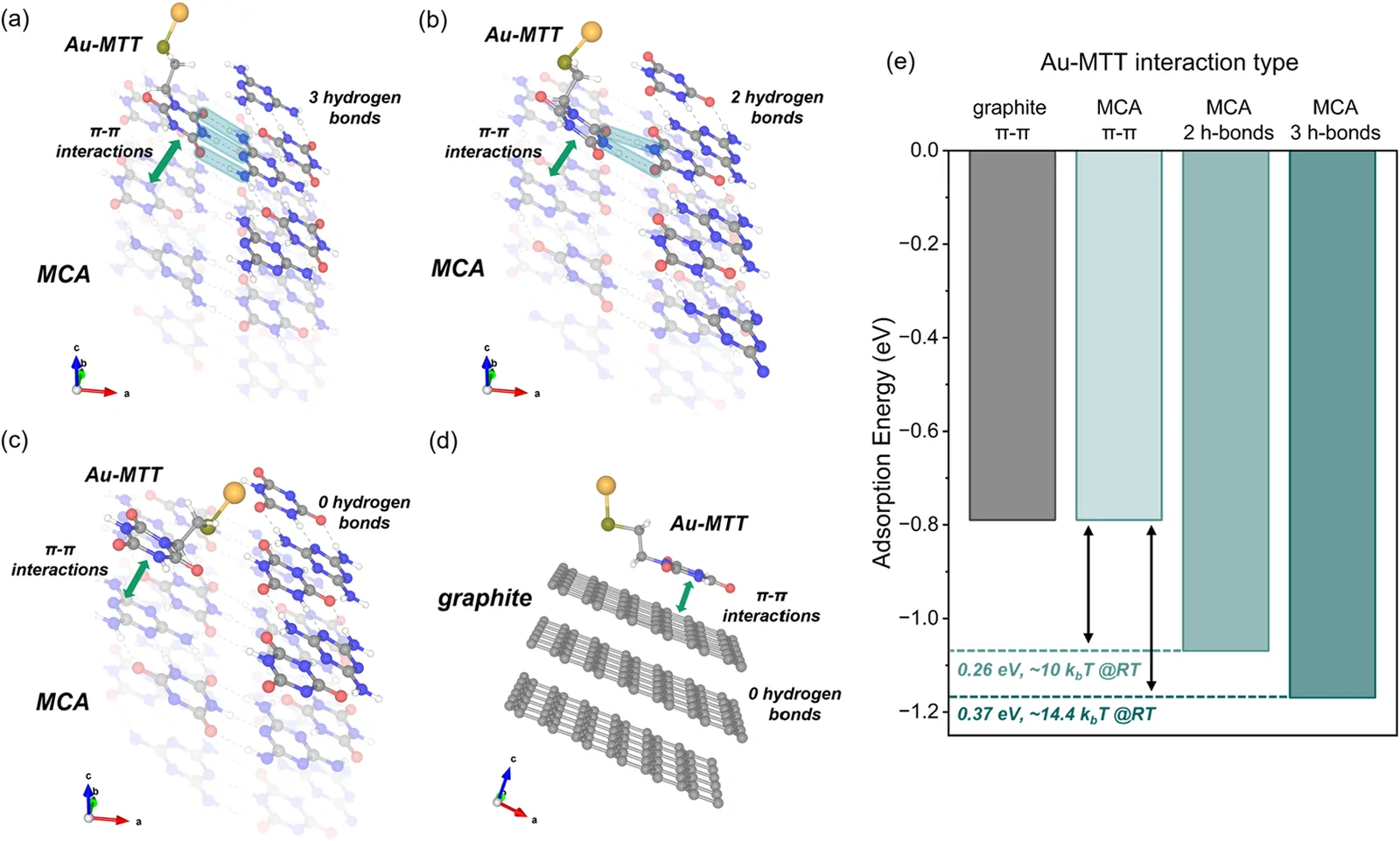
Representative lowest-energy adsorption configurations of Au-MTT on MCA exhibiting π–π stacking with or without accompanying hydrogen bonding (highlighted in teal): (a) molecular recognition with melamine, forming 3 hydrogen bonds (3H-A), (b) molecular recognition with cyanuric acid, forming 2 hydrogen bonds (2H-A), and (c) 0 hydrogen bonds (only nonspecific π–π interactions). (d) Lowest-energy adsorption configuration of Au-MTT on graphite (0H-C), where Au-MTT adsorbs nonspecifically via π–π interactions. (e) Zero-point corrected adsorption energies (*E*_ads_) of Au-MTT on three possible MCA sites (3H-A, 2H-A, 0H-A) versus Au-MTT on graphite (0H-C).

Geometry optimizations demonstrate that the configuration with three hydrogen bonds (3H-A) gives the most favorable adsorption (−1.16 eV), with a minor difference in binding strength as the number of hydrogen bonds decreases to two (−1.05 eV, 2H-A). This ∼0.1 eV difference could reflect minor differences in binding configuration and indicates that both melamine and cyanuric acid subunits provide comparable binding strength through molecular recognition.^37,82^ In contrast, adsorption via nonspecific π–π stacking alone without hydrogen bonds (0H-A, Figure 4c) is significantly weaker (by at least 0.26 eV; ∼10 *k*_B_·*T* at 298 K). This indicates that molecular recognition through complementary hydrogen-bond donor–acceptor pairs dominates Au-MTT adsorption on MCA.

To further contextualize the relative strength of the nonspecific π–π interactions, we calculated Au-MTT adsorption on graphite, where only π–π stacking is available on the ideal surface. The geometry-optimized configuration of Au-MTT on graphite shows an adsorption energy of −0.79 eV (0H-C, Figure 4d,e), identical to that of the π–π stacking configuration on MCA (0H-A, Figure 4e, Table S4), indicating that the two supports offer Au-MTT comparable nonspecific basal-plane interactions. The distinction between these two supports lies at the sheet edges, where MCA exposes molecularly uniform hydrogen-bonding sites while graphite’s edges are themselves defects in the intralayer covalent network. Any hydrogen-bonding moieties graphite presents at these edges (among other defects we do not probe computationally here) are therefore inherently nonuniform in distribution and strength.^83,84^ We postulate that such sites contribute to the nonuniform distribution of Au-MTT complexes observed by TEM and HAADF-STEM (Figure S12). Collectively, these results demonstrate that molecular recognition provides what neither nonspecific stacking nor defect-mediated binding can offer—namely uniform and molecularly defined adsorption sites of sufficient strength—enabling molecular-scale dispersion of the Au-MTT precursor prior to NP nucleation during thermal treatment.

#### 3.2.3 Formation of Monodisperse, Ligand-Free Au NPs upon Thermal Treatment

As in metal-MPTMS-functionalized SiO_2_, thermal treatment is needed to remove the organic species and create catalytically active NPs.^74,75^ Upon thermal treatment of Au-MTT-MCA-as in N_2_ and air, XPS in the S 2p region shows residual sulfur signals (Figure S17a). However, the same thermal treatment in H_2_ results in elimination of the S signal in XPS (Figure S17a), indicating that MTT removal proceeds by reductive cleavage of the Au-thiolate bond rather than thermal desorption at this temperature. In contrast, the higher thermal treatment temperature used for Au-MPTMS-SiO_2_-as (550 °C) eliminates the S signal from MPTMS even in air (Figure S17b). The removal of MTT under H_2_ is supported by quantitative sulfur analysis (ICP), which showed S content below the limit of quantification of the measurement (≤0.01 wt % S), as obtained for the bare MCA support. TEM images of Au-MTT-MCA show particles with an average diameter of 2.5 ± 0.5 nm (Figure 5a,c). Bright-field (Figure S18a) and HAADF-STEM images (Figure 5b) show finely dispersed NPs decorating the sheet edges of the MCA rods, consistent with nucleation from the edge-bound Au-MTT complexes indicated by DFT. While the beam sensitivity of MCA greatly limits high-resolution TEM imaging, lattice fringes could be resolved for one Au NP, with a measured d-spacing of 2.4 Å consistent with fcc Au(111) (Figure S18b). The dispersity index (DI = 1.1, Table 1) is consistent with excellent monodispersity,^45,85^ which is notable given that neither ligands nor a high surface area, high surface functionality support is required.

**Figure 5:**
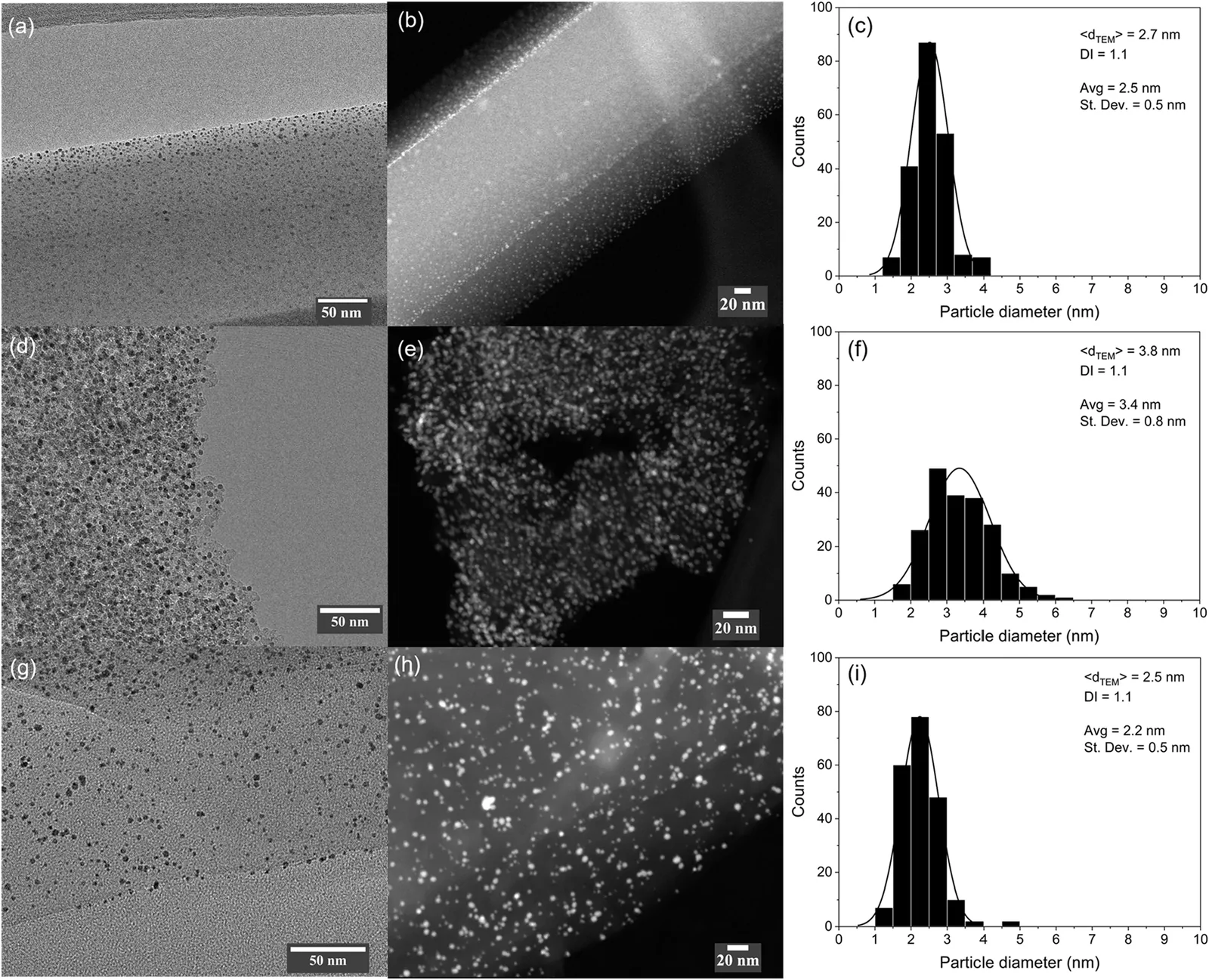
(a, d, g) TEM images, (b, e, h) HAADF-STEM images, and (c, f, i) particle size distributions of supported Au NPs prepared on MCA (Au-MTT-MCA, top), silica (Au-MPTMS-SiO_2_, middle), and graphite (Au-PVA-G, bottom).

For comparison, the bridge method on silica yields 3.4 ± 0.8 nm NPs (Figure 5d–f),^75^ while traditional sol immobilization with PVA ligands on graphite yields 2.2 ± 0.5 nm NPs (Figure 5g–i).^86^ Both likewise exhibit high monodispersity (Table 1). In contrast, thermal treatment of Au-MTT-G-as, in which the Au-MTT precursors were nonuniformly dispersed, results in polydisperse NPs spanning ∼2 nm particles to ∼20 nm agglomerates (Figure S19a,b; 2.6 ± 2.1 nm, DI = 1.5, just above the threshold for monodispersity^45^). The contrast to Au-MTT-MCA (DI = 1.1) traces monodispersity directly to the uniformity of the dispersed Au-MTT complexes, and by extension, to the molecular recognition that enables it. Additional comparands for the catalytic studies (Au-PVA-MCA, Au–P25 TiO_2_) were prepared by established methods (Table S3, Figure S19c–f).

### 3.3 Interfacial Inertness of MCA and Its Catalytic Consequences in Selective Oxidative Dehydrogenation

#### 3.3.1 Absence of EMSIs at the NP-Support Interface

High-resolution XPS was used to elucidate the electronic nature of the Au-MCA interface and compare it against the NP-support interfaces on graphite and silica, supports reported to lack EMSIs.^75,86^ In the Au 4f region (Figure 6a), Au-MTT-MCA has peaks centered at 84.1 and 87.7 eV corresponding to the Au 4f 7/2 and 5/2 signals, respectively. These binding energies are consistent with metallic Au^0^ and are identical (within the instrument resolution, 0.2 eV) to those obtained for Au NPs prepared by MPTMS on silica and sol immobilization on graphite with PVA ligands (Table 2); we attribute the broader peak widths on the MCA and silica-supported Au NPs compared to those on graphite to charging due to their insulating nature.^87^ The absence of a shift relative to these benchmarks demonstrates that MCA is electronically inert at the NP-support interface.^88,89^ In contrast, Au NPs on P25 TiO_2_ give rise to a Au 4f 7/2 signal shifted by 0.7 eV (83.4 eV, Figure S20), indicating EMSIs in the form of electron transfer from the TiO_2_ support to the Au NPs.^88,90^

**Figure 6:**
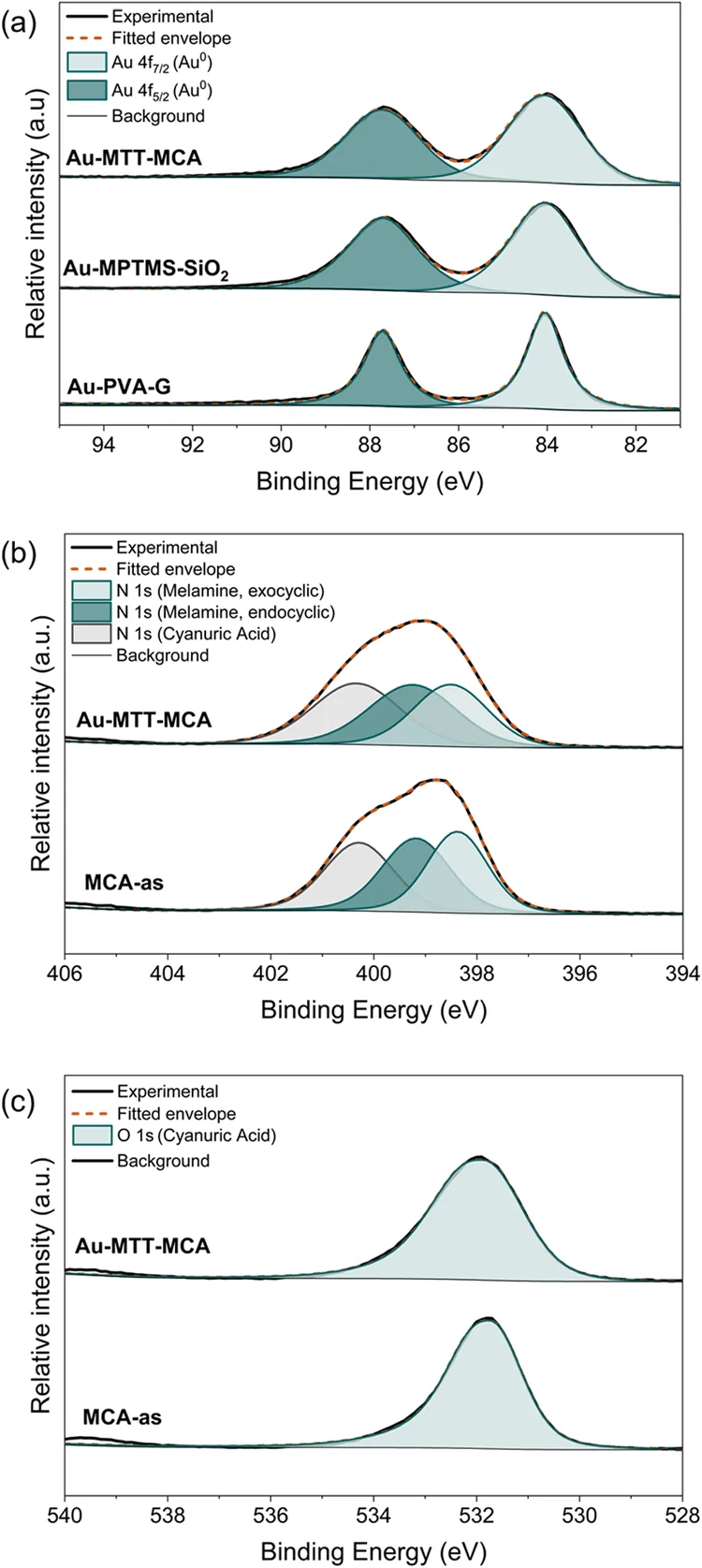
High-resolution (a) Au 4f XPS of supported Au NP catalysts and (b) N 1s and (c) O 1s spectra of MCA-as and Au-MTT-MCA.

**Table 2:** Binding Energies Measured by High-Resolution O 1s, N 1s, and Au 4f XPS in Various Samples^a^

sample	O 1s (eV)	N 1s, melamine exocyclic (eV)	N 1s, melamine endocyclic (eV)	N 1s, cyanuric acid (eV)	Au 4f 7/2 (eV)
MCA-as	531.7	398.4	399.2	400.3	n/a
Au-MTT-MCA	531.9	398.5	399.2	400.3	84.1
Au-MTT-MCA-spent	531.8	398.4	399.3	400.4	84.1
Au-MPTMS-SiO_2_	n.q.	n/a	n/a	n/a	84.1
Au-PVA-G	n.q.	n/a	n/a	n/a	84.0

a n.q. = not quantified.

To probe whether the MCA support itself is electronically modified due to the Au-MCA interface, high-resolution XPS spectra in the N 1s (Figure 6b) and O 1s (Figure 6c) regions were collected on Au-MTT-MCA. These spectra show that neither the binding energies of N atoms nor O atoms shift with Au NPs present (Table 2), indicating the absence of measurable electron transfer from the support side of the interface. We do observe a modest broadening in the FWHM (Table S5) for both N and O environments relative to bare MCA, which does not occur in the absence of Au and is therefore attributed to increased heterogeneity in local charge distributions at surface N and O atoms, rather than to any net electron transfer.^91^ Collectively, XPS of the metal-MCA interface reveals no measurable charge transfer in either direction, confirming the absence of EMSIs at the Au-MCA interface.

#### 3.3.2 Catalytic Reactivity of Supported Au NPs in the Aerobic Oxidative Dehydrogenation of MP

Having characterized the electronic and structural properties of the MCA-supported Au NPs, we now examine their behavior in the aerobic oxidative dehydrogenation of MP to the enaminone product MDHP (Scheme 1). Since MP and MDHP are susceptible to side reactions, selectivity serves as a probe of interfacial contributions to the catalytic cycle. We assess these contributions, together with those of NP size and ligand state, across the full set of supported Au catalysts. This set spans conventional supports of varying surface functionality (graphite, silica, and P25 TiO_2_) and both ligand states (bare and PVA-capped) on each of MCA and graphite, enabling support and ligand contributions to be varied independently.

**Scheme 1:**
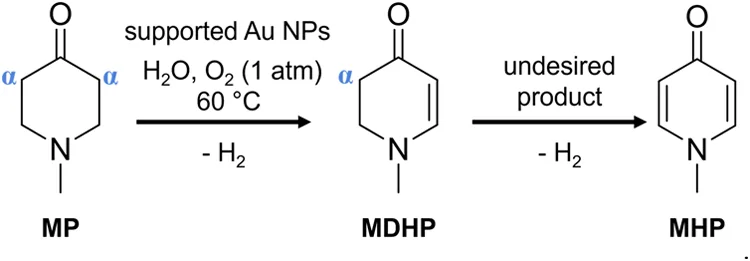
Aerobic Oxidative Dehydrogenation of MP to MDHP and Sequential Oxidative Dehydrogenation to MHP

Control reactions (Figure S21) in O_2_ with MCA alone or with no catalyst show zero MDHP yield, and reaction under Ar on Au-MTT-MCA yields only ∼3% MDHP, which we attribute to residual O_2_ in the system (rigorous purging is not feasible due to the high volatility of MP). Binding energies at the Au-MCA interface are unchanged after catalysis (Table 2, Figure S22a–c), and mass recovery of the spent catalyst is nearly 100%, indicating that MCA does not participate directly or indirectly as an O_2_ source or sink. ICP of the reaction liquor shows no detectable Au, confirming heterogeneous catalysis by support-bound NPs. Upon recycle, Au-MTT-MCA retains activity with only a modest drop in yield (Figure S22d), attributed to aggregation of a minor fraction of NPs (2.8 ± 1.5 nm by TEM of the spent catalyst, Figure S22e,f), while MCA retains its long-range order and morphology (XRD and SEM, Figure S22g,h). Collectively, these tests indicate that α-H abstraction of MP, reported as the rate-determining step on other Au NP catalysts under similar conditions,^92^ proceeds by activation of O_2_ on Au without redox activity from MCA, and that MCA is a stable support under these catalytic conditions. Since graphite and silica also lack lattice-based redox-active sites, α-H abstraction may be expected to proceed similarly on the NPs they support.

The conversion profiles of MP and yields of MDHP over time, site-time-yields (STYs), and MDHP selectivity profiles as a function of MP conversion are summarized in Figure 7a–c. The conversion and yield profiles for Au-MTT-MCA track closely at all times, consistent with MDHP being the only product detected by ^13^C NMR analysis in the reaction liquor (Figures 7a and S23). In contrast, Au-MPTMS-SiO_2_ shows the formation of a secondary product whose chemical shifts can be assigned to 1-methyl-4­(1*H*)-pyridinone (MHP); Au-PVA-G shows only minor production of MHP (Figures 7c, S23, and S24). By 6 h, the MDHP yields are 90% on Au-MTT-MCA, 69% on Au-PVA-G, and 60% on Au-MPTMS-SiO_2_. The reactivity on these three catalysts may be influenced by NP size, support contributions, and/or ligand effects. To account for differences in NP size, the STYs quantify MDHP formation per-surface Au, as estimated from ⟨*d*_TEM_⟩ (Figure 7b).^45,74^ If size were the only contributing factor to the reactivity trends, the per-Au activity should align for all catalysts. However, particularly at early times (<2 h), MDHP production on Au-MTT-MCA is ∼2× that on Au-PVA-G, and ∼0.5× that on Au-MPTMS-SiO_2_. It is possible that site-type population differences contribute, which STY would not account for. Approximating site type fractions using a truncated cuboctahedron model for Au NPs (Table S6),^93,94^ Au-MTT-MCA and Au-PVA-G have nearly identical site populations, indicating that their different performances arise from support and/or ligand contributions. On the other hand, Au-MPTMS-SiO_2_ has more substantial differences in densities of edges and corners (∼25 and 50% lower, respectively), which could enhance STY if such sites are inhibitory (e.g., by binding intermediates too strongly). However, even if site populations play a role, the analysis below (Section 3.3.3) suggests that support effects also contribute substantially to the overall reactivity of the Au-MPTMS-SiO_2_ catalyst.

**Figure 7:**
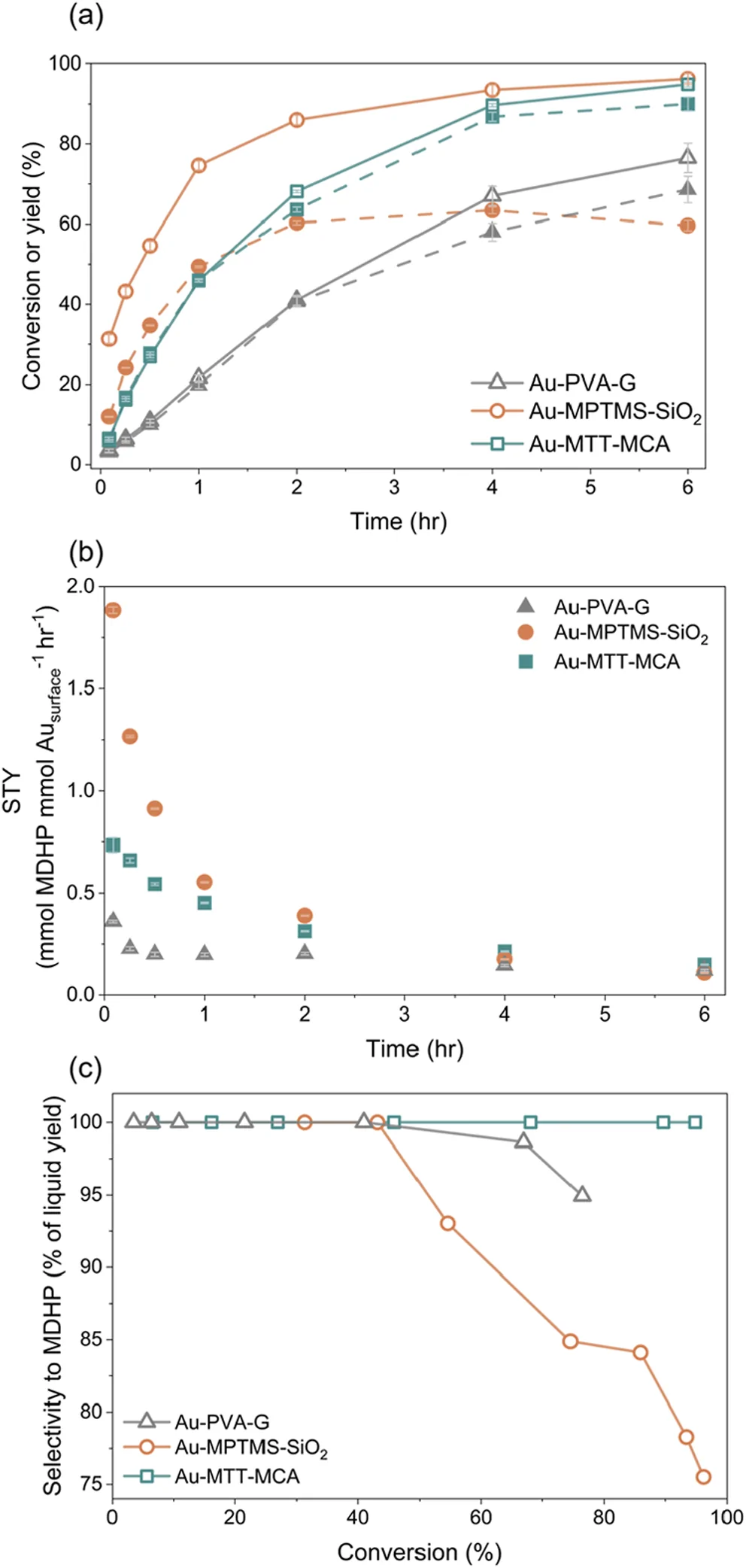
(a) MP conversion (open symbols) and MDHP yield (closed symbols); (b) STYs toward MDHP formation; and (c) MDHP selectivity vs MP conversion of Au NPs on different supports in the oxidative dehydrogenation of MP: 0.1 mmol of MP, 0.1 mmol of NMP (internal standard), 2 mL of DI H_2_O, 1 bar O_2_, 50 mg of catalyst, 60 °C. Lines in (a) and (c) are intended to guide the eye.

To characterize the degree of support contributions to the activity differences on Au-MTT-MCA and Au-PVA-G, Au-MTT-G and Au-PVA-MCA were evaluated to decouple ligand effects. Figure 8 shows the MDHP yield and MP conversion rates, normalized per surface Au. Au-MTT-G exhibits a lower 6 h-yield than Au-MTT-MCA (79 vs 90%, Figures 8 and S25). This cannot be attributed to different electronic environments of the Au NP since all catalysts have the same oxidation state (i.e., Au^0^) as demonstrated by XPS (Figures 6 and S20). Normalizing for surface Au, Au-MTT-MCA and Au-MTT-G have similar MP conversion rates. Thus, the lower MDHP yield on Au-MTT-G is attributed to its larger, polydisperse NPs. The liganded NPs behave comparably in yield and conversion rate regardless of support, but convert MP more slowly than the bare NPs, suggesting that PVA passivates surface sites for O_2_ activation.^95^ These comparisons assign the differences among MCA- and graphite-supported catalysts entirely to NP size and ligand state. With no residual support effect, MCA behaves equivalently to graphite in this reaction. As MCA does not enhance the intrinsic reactivity of Au, its advantage is synthetic, affording the bare, monodisperse NPs that graphite cannot while matching graphite’s interfacial inertness during catalysis, which we establish directly for MP and MDHP below.

**Figure 8:**
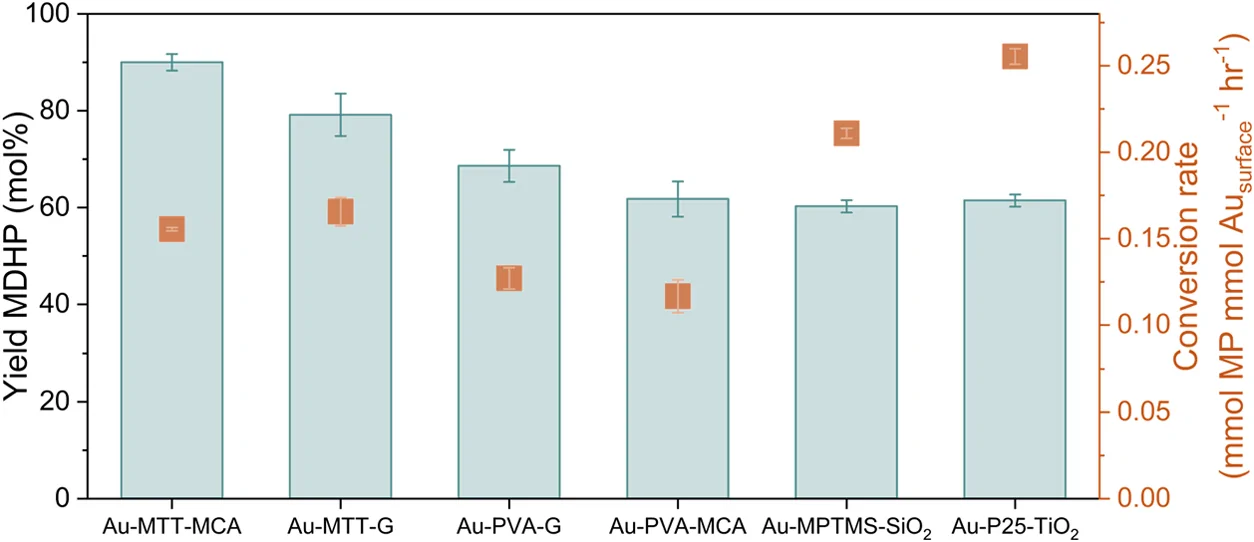
Yields of MDHP and conversion rates of MP measured at 6 h over supported Au NP catalysts. Conditions: 0.1 mmol of MP, 0.1 mmol of NMP (internal standard), 2 mL of DI H_2_O, 1 bar O_2_, 50 mg of catalyst, 60 °C.

#### 3.3.3 Origin of Selectivity Differences: Reactant-Pool-Support Interactions

On a per surface-Au basis, the ligand-free Au NPs supported on graphite and MCA are less active than those supported on silica and P25 TiO_2_ as a representative “active” support (MP conversion rate, Figure 8). Since these Au NPs are also ligand-free, this indicates that support contributions are responsible. On silica, Au NPs have the same electronic environment as that on graphite and MCA (Figure 6a), so their reactivity differences cannot be attributed to EMSI effects. Thus, silica is nonreactive at the NP-support interface, but reactive at the reactant-pool-support interface: even in the absence of a catalytically active site (i.e., Au), the adsorption of MP to the support is significantly higher on SiO_2_ (21 mol %) compared to graphite (∼1%) and MCA (<1%) (Table 3). We attribute this to its high surface area (∼10–100× larger than that of MCA and graphite) and many acidic silanols that can readily bind carbonyl compounds, which act as a hydrogen-bond acceptor.^96–98^

**Table 3:** Adsorption of MP on Different Bare (Au-Free) Supports^a^

catalyst support	adsorption of MP (mol %)
MCA-as	0.4
graphite	1.3
SiO_2_ gel	21
P25 TiO_2_	2.3

a Conditions: 0.1 mmol of MP, 0.1 mmol of NMP (internal standard), 2 mL of DI H_2_O, 1 bar O_2_, 50 mg of catalyst, 60 °C.

Prior to discussing the support contributions in more detail, we confirmed that the observed selectivity on silica is not from differences in site population influencing the sequential oxidative dehydrogenation step. To do this, larger Au NPs were formed on graphite via calcination of Au-PVA-G. Calcination produces a broad, tailed distribution containing particles substantially larger than those in any of the three catalysts (Figure S26a,b), and therefore a higher overall fraction of terrace sites with fewer edge and corner sites, while retaining the ∼2.5 nm population of the parent sample. Catalysis on this sample showed MDHP formation and no MHP detected in the reaction liquor by ^13^C NMR (Figure S26c), in contrast to the substantial MHP formation observed on Au-MPTMS-SiO_2_ (19% yield at 6 h). This demonstrates that the sequential oxidative dehydrogenation of MDHP to MHP observed on Au-MPTMS-SiO_2_ cannot be attributed to its larger particle size (⟨*d*_TEM_⟩ = 3.8 nm) relative to Au-MTT-MCA and Au-PVA-G and its correspondingly higher terrace and lower edge/corner populations. Thus, we attribute this selectivity difference to reactant-pool-support interactions, which have direct consequences for the reactivity of Au-MPTMS-SiO_2_.

First, this causes adsorption without liquid-phase product generation, as demonstrated by the 30% conversion of MP with only 10% yield of MDHP (and no yield of MHP) at 5 min of reaction time (Figures 7a and S24). Thermal gravimetric analysis on the spent catalyst demonstrates that this adsorbed MP converts to carbonaceous deposits by only 30 min of reaction time (Figure S27a). After this time, only liquid-phase products are detected. Second, we postulate that silanols facilitate enhanced MDHP production at early reaction times based on the higher early-time STY on silica in this regime (Figure 7b). The silanols may increase the rate of the first oxidative dehydrogenation to MDHP by increasing the surface coverage of MP near the active sites (Au NPs), activating the carbonyl to increase the acidity of the α-H, or through silanol-mediated stabilization of transition states, which collectively have been observed to play key roles in related reactions involving carbonyl-containing substrates on silica.^99–104^ While these interactions are favorable for increasing the STY of MDHP, they also likely play a key role in reducing selectivity over time due to MHP formation. Based on the mechanism, MHP may form as a secondary product via the oxidation of MDHP, owing to the relatively facile deprotonation of the second α-H.^92,105^ Adsorption of MDHP by surface silanols can help facilitate this second deprotonation step, compromising product selectivity.^106^ The loss in MDHP selectivity on Au-MPTMS-SiO_2_ is observed at only 30 min of reaction time, which aligns with the onset of detection of MHP (Figure S24). This indicates that MHP forms in appreciable quantities as MDHP accumulates, causing a progressive drop in MDHP selectivity over time and resulting in a 19% yield of MHP by 6 h.

In contrast to silica, where support contributions during the oxidative dehydrogenation reaction are dominated by reactant-pool-support interactions via silanols, P25 TiO_2_ is largely noninteracting with MP by adsorption (Table 3), similar to MCA and graphite. This indicates that its high MP conversion rate and low STY of MDHP (Figure 8) are attributable to other support effects. First, higher electron density on Au NPs supported on P25 TiO_2_ (Au 4f 7/2 at 83.4 eV, Figure S20) can facilitate the formation of stronger oxidizing intermediates, e.g., superoxide, through more favorable and facile electron transfer.^92,107^ Second, an Ar control (Figure S28) shows that P25 TiO_2_ can directly facilitate MP conversion in the absence of O_2_ through a lattice oxygen-mediated mechanism, as observed previously on Au NPs supported on OMS-2,^92,108^ although the activity is 4× lower than in O_2_. The net effect of these support features is high conversion of MP (93%, Figure S25) but lower MDHP yield (62%) due to MHP formation (19%) and carbonaceous deposits (Figure S27b). Together, these comparisons trace each catalyst’s behavior to its interfaces. Contributions at either interface, whether by substrate adsorption (silica) or electron transfer with redox capability (P25 TiO_2_), sacrifice selectivity; graphite’s interfaces are inert, enabling high selectivity, but its requisite PVA ligands suppress conversion; and Au-MTT-MCA, uniquely combining interfacial inertness with small, bare NPs, enables full MDHP product selectivity and achieves 90% yield at 6 h, compared to 60–69% on conventional supported catalysts.

To further evaluate reactant-pool-support contributions to the secondary oxidation of MDHP (Scheme 1), we conducted a controlled serial oxidation experiment. Of the two support-active catalysts, we focus on silica because its Au NPs are electronically identical to those on MCA and graphite, while those on P25 TiO_2_ have confounding EMSI contributions. Since the per-surface Au normalization of the STYs further accounts for differences in NP size, reactant-pool-support interactions remain the differentiating variable. Using MDHP as the reactant, STYs for MHP production are 0, 0.1, and 3.3 mmol MHP surface-Au^–1^ h^–1^ on Au-MTT-MCA, Au-PVA-G, and Au-MPTMS-SiO_2_, respectively, in the absence of MP (Figure 9). These results demonstrate that MHP can form directly from MDHP via a support-facilitated serial oxidation step, whose extent correlates with the surface functionality. Even with a high concentration of MDHP that can, in principle, enhance surface coverage and promote the serial oxidation route, graphite and especially MCA retain catalytic inertness to the MDHP substrate, while silica promotes oxidation to MHP.

**Figure 9:**
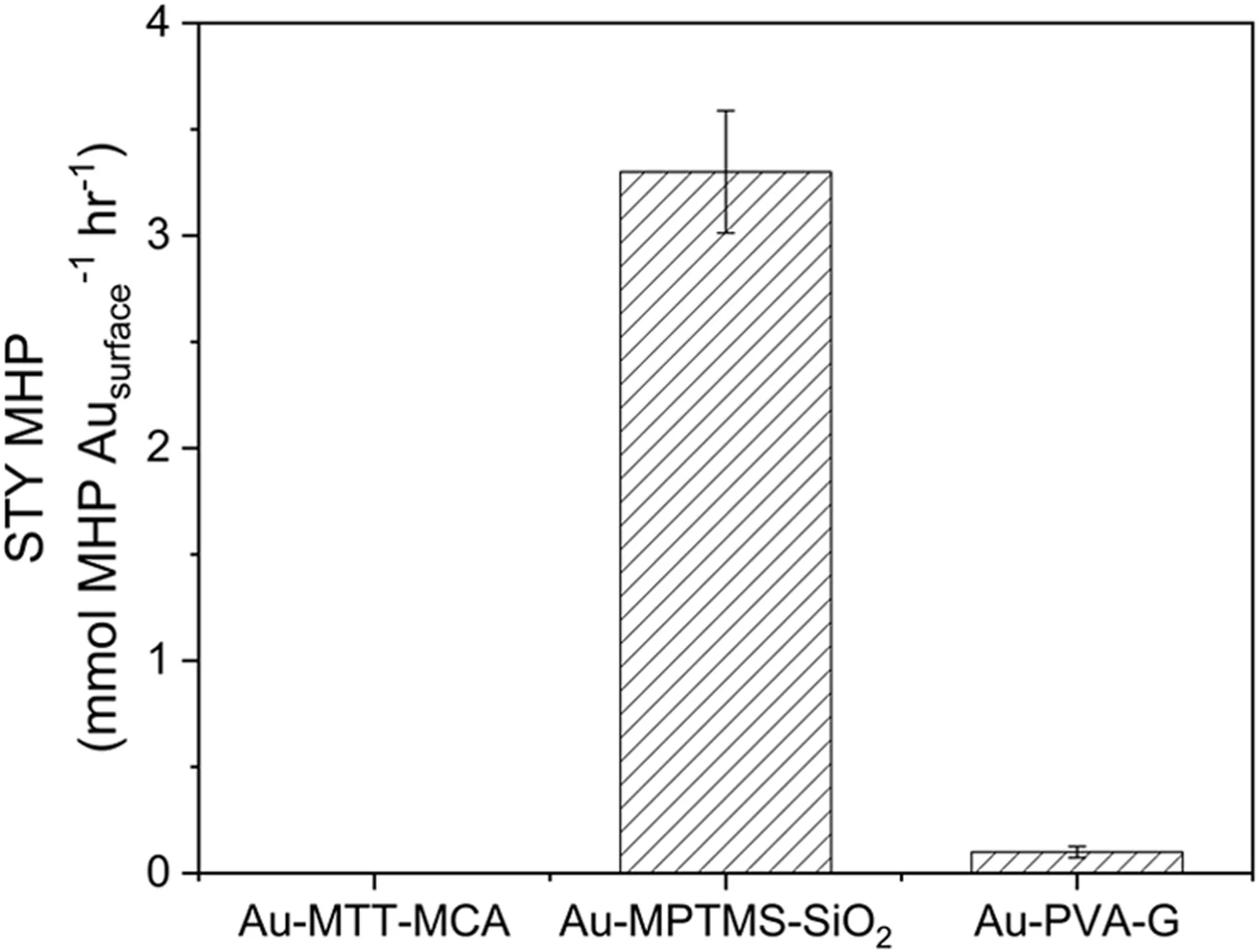
STY of MHP in MDHP oxidation: 0.03 mmol of MDHP, 0.03 mmol of NMP (internal standard), 2 mL of DI H_2_O, 1 bar O_2_, 20 mg of catalyst, 60 °C, 6 h.

Collectively, these results establish the interfacial inertness of MCA as a catalytic support for Au NPs in the selective oxidative dehydrogenation of MP to the target enaminone product, MDHP, and demonstrate the value of a catalyst with an explicitly defined active site environment free of ligand and support contributions such that their effects can be disentangled: under these conditions, the overoxidation of MDHP is fully attributable to reactant-pool-support interactions, rather than being intrinsic to Au^0^ NPs themselves.

## 4 Conclusion

In summary, we introduced a synthetic strategy based on molecular recognition to functionalize the supramolecular material, MCA, with small (2.5 ± 0.5 nm), monodisperse (DI = 1.1), ligand-free Au NPs, mediated by MTT as a bridge prior to thermal treatment. High-resolution XPS, TEM and HAADF-STEM, ICP, and DFT analysis, coupled with control studies on graphite, demonstrated that Au binds to MTT at its thiol site, forming Au-MTT complexes that disperse onto MCA supports through complementary hydrogen-bond donor–acceptor pairs between the cyanuric acid backbone of MTT and exposed melamine and cyanuric acid subunits at MCA’s sheet edges. The molecular-level uniformity afforded by the lattice-based edge sites, distinct from defect terminations, maps to the uniformity in Au NPs obtained upon removal of the MTT bridge and stems directly from MCA’s supramolecular construction. Despite its noncovalent backbone, we demonstrated the relatively wide stability windows of MCA through thermal and solvent-based stress tests, along with structural characterization after deployment in the aerobic selective oxidative dehydrogenation of MP, indicating its potential as a catalyst support, e.g., under mild liquid-phase oxidation conditions.

While MP conversion rates and MDHP selectivity profiles were convolved by ligand and support effects on conventional catalysts (graphite, silica, P25 TiO_2_), Au-MTT-MCA uniquely afforded separable analyses of these contributions, along with 100% liquid-phase selectivity and the highest MDHP yield. Referencing against its bare Au^0^ surfaces assigns PVA ligands a single role, suppressing MP conversion without significantly influencing selectivity, and resolves silica’s contributions into two distinct interactions: support-mediated MP adsorption that enhances per-surface-Au MDHP production, and MP and MDHP binding at the silica interface that drives carbonaceous deposit formation and overoxidation. These assignments, which are inaccessible on conventional catalysts, motivate the design of support functionality that maintains Au^0^ NP character while balancing the competing effects of adsorption on activity and selectivity. More broadly, we anticipate that the recognition-based synthetic strategy developed here can extend to other metal-bridge-support interfaces with distinct framework structures, recognition motifs, and metal-binding chemistries, widening the design space for supported metal NP catalysts.

## Supplementary Material



## Notes

Characterization (^1^H and ^13^C NMR, XRD, SEM, XPS, and TEM/HAADF-STEM), stability tests, and catalysis (additional comparands, controls, and recycle tests) (PDF)
