# Supplementary material for: Molecular Recognition Affords Ligand-Free Au Nanoparticle Catalysts on an Interfacially Inert Supramolecular Support
Source: Chem Mater. 2026 Sep 28;38(19):10282–97. doi: 10.1021/acs.chemmater.6c01997 (PMC13673943; doi:10.1021/acs.chemmater.6c01997)
Supplement: Supplementary file 1 [file cm6c01997_si_001.pdf]

Supporting Information for

Molecular Recognition Affords Ligand-Free Au Nanoparticle Catalysts on an Interfacially Inert Supramolecular Support

Kushaan Bahl,[†] *Gerald Siu Hang Poon Ho,*[^] *Brandon C. Bukowski,*[^] *Marcella Lusardi*[†‡*]

[†]Department of Chemical and Biological Engineering, Princeton University, Princeton, NJ 08544 United States

[‡]Princeton Materials Institute, Princeton University, Princeton, NJ 08544 United States

[^]Department of Chemical and Biomolecular Engineering, Johns Hopkins University, Baltimore, MD 21218

*mlusardi@princeton.edu

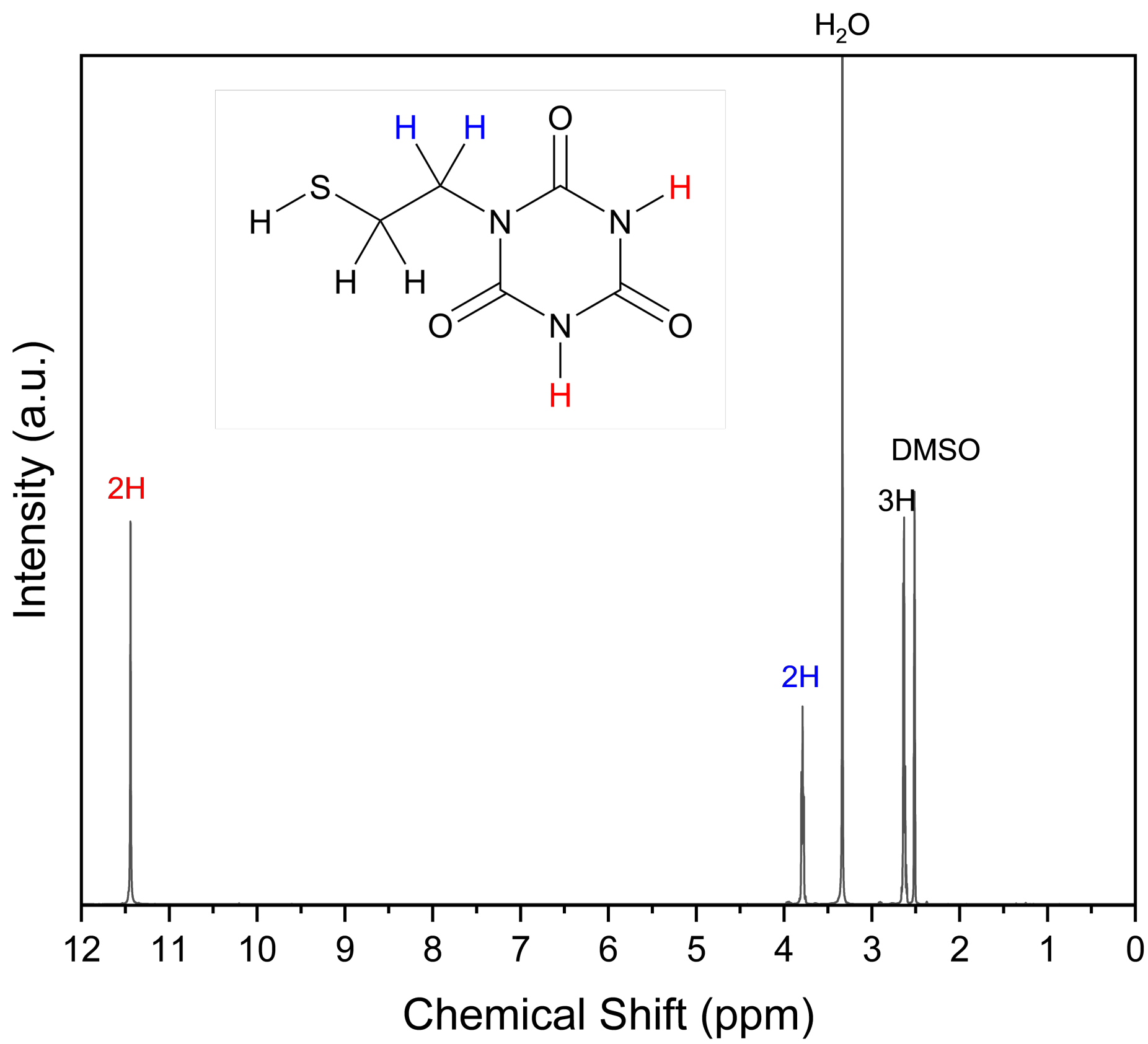

Figure S1. ^{1}H NMR of MTT in DMSO-d6. δ (ppm): 2.66 (m,3, CH_2S + SH; black), 3.80 (m,2, CH_2N; blue), and 11.45 (s,2, NH; red).[1] Peaks corresponding to residual hydrogens in DMSO-d6 (2.5 ppm) and H_2O (3.4 ppm) are impurities.

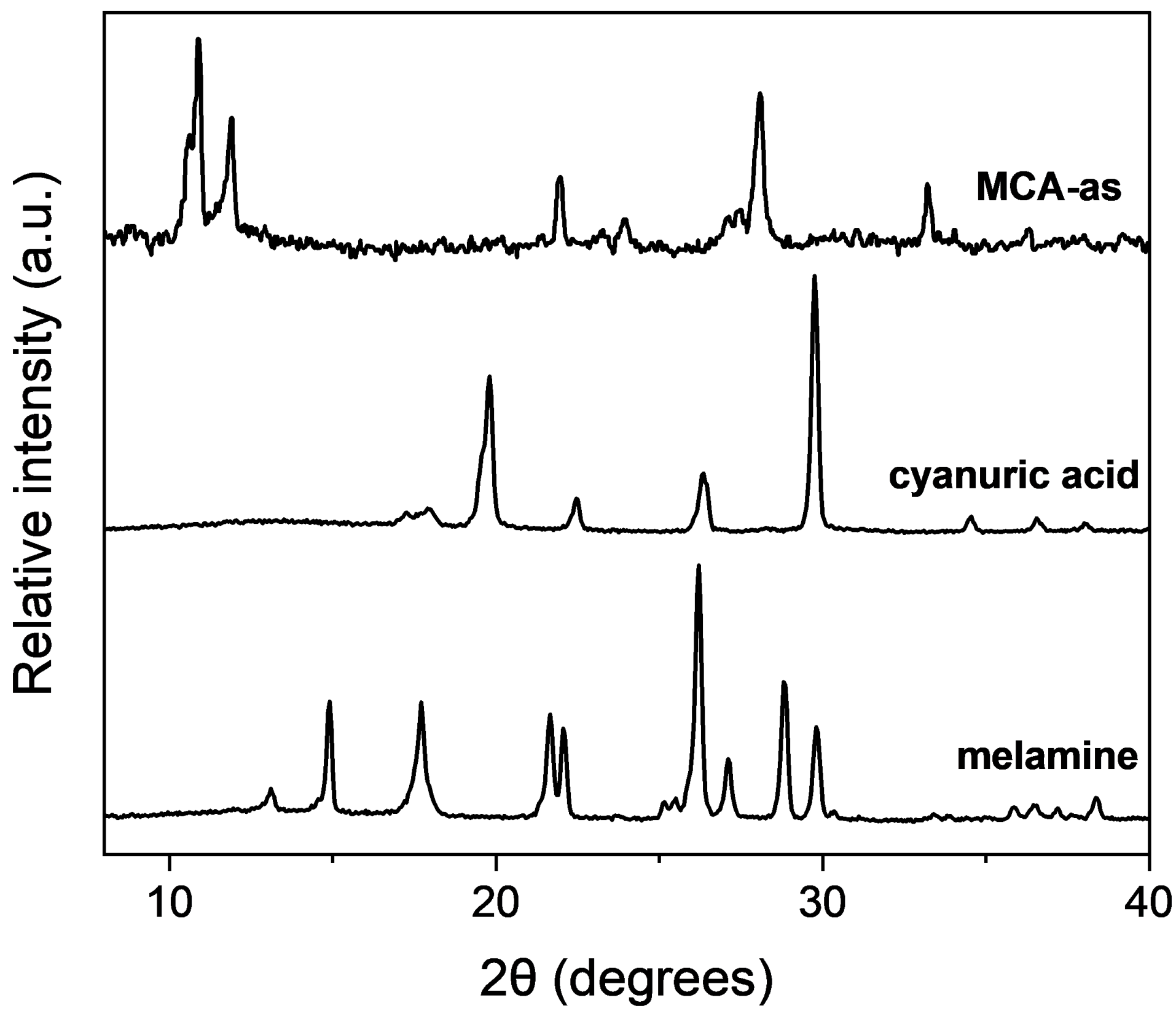

Figure S2. Powder XRD patterns of melamine, cyanuric acid, and MCA-as.

The XRD pattern for MCA-as shows the characteristic peaks at 10.6º and 27.9º that can be assigned to (110) (from intralayer hydrogen bonds) and (202) (from interlayer π-π stacking interactions), respectively (Figure S2).[2]

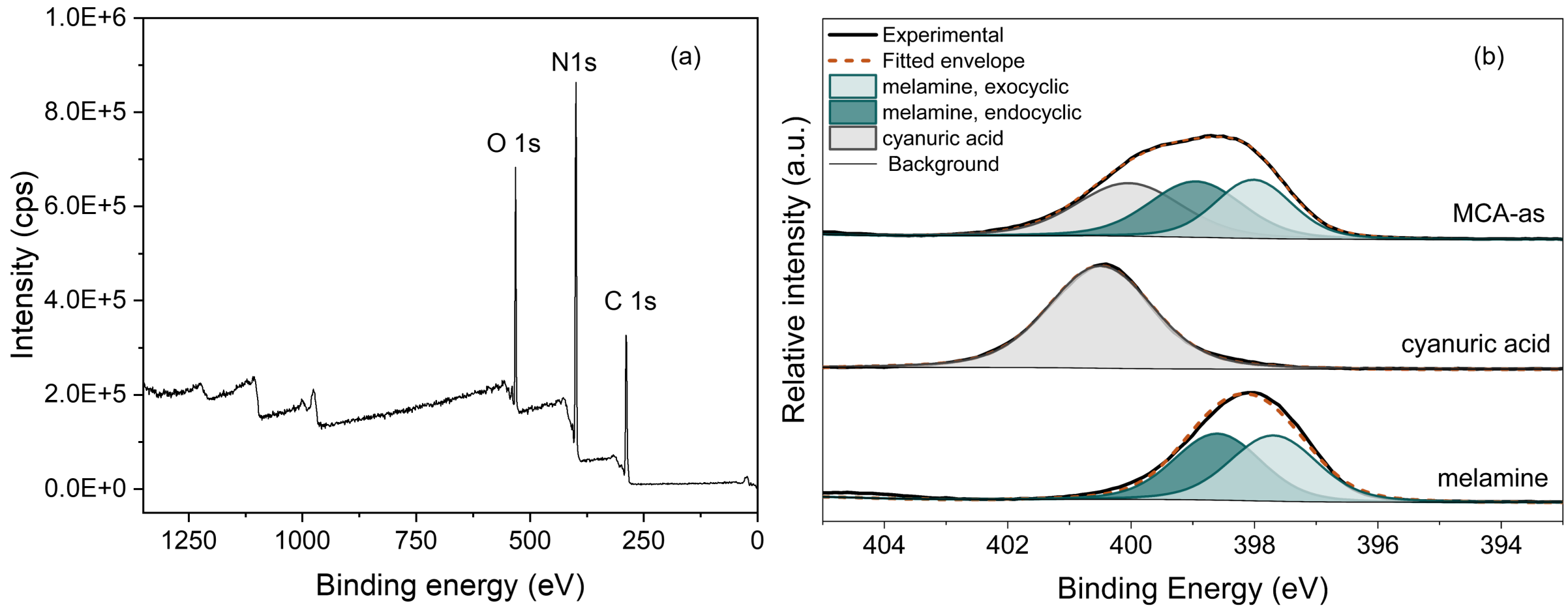

Figure S3. (a) Survey XPS spectrum of MCA-as. (b) High-resolution N 1s XPS spectra of melamine, cyanuric acid, and MCA-as, with curve fitting for MCA-as corresponding to N atoms in cyanuric acid, melamine (endocyclic), and melamine (exocyclic).

The XPS survey spectrum of MCA-as in Figure S5a shows the expected peaks attributable to the presence of C, N, and O on the MCA surface. High-resolution XPS spectra in the N 1s region (Figure S5b) show that the binding energies of surface N atoms in melamine (397.8, 398.7 eV) and cyanuric acid (400.5) shift in MCA-as to 398.5, 399.3, and 400.3, respectively, consistent with previous reports in MCA formation from its monomers.[3]

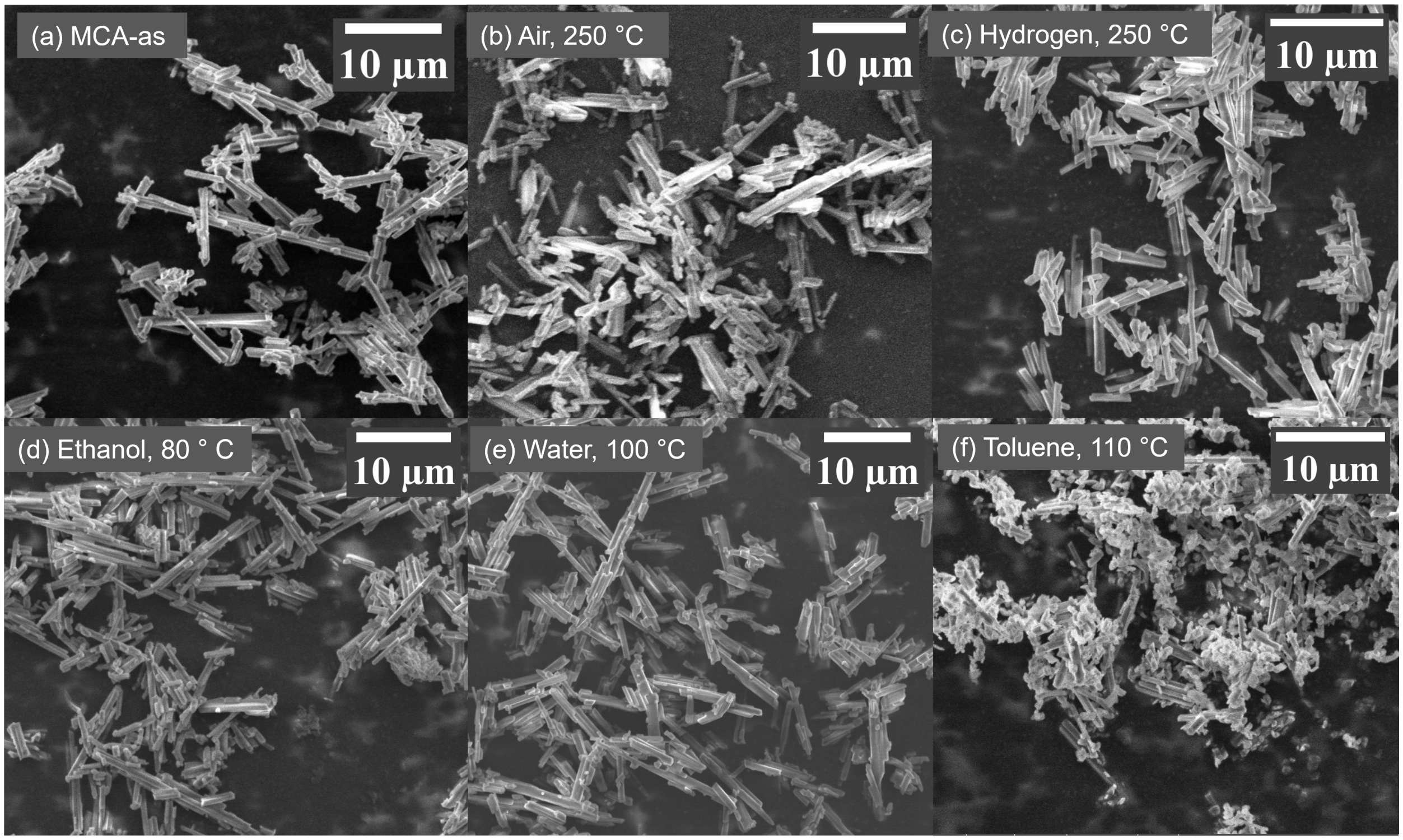

Figure S4. SEM images of (a) MCA-as and MCA after being subjected to 250 °C in (b) air or (c) hydrogen; or after refluxing in (d) ethanol, (e) water, or (f) toluene. All treatments were carried out for 6 hr.

Stability tests and results

Since MCA has a noncovalent backbone, characterizing its stability limits is particularly important in the context of catalytic applications. While there have been studies on its thermal stability under a temperature ramp and general solvent stability at room temperature,[4,5] there is lacking information regarding its stability under the relatively harsh conditions experienced during both catalyst processing (e.g., reduction steps) and catalytic environments (e.g., hot solvents, under agitation). We aimed to address this knowledge gap and validate MCA as a support candidate for a subset of catalytic domains. To do this, MCA was subjected to oxidizing (air) and reducing (H_2) environments at 250 °C, as well as refluxing solvents with varied polarity (water, ethanol, and toluene). 250 °C was chosen for the gaseous treatments since the onset of decomposition of MCA is ~300 °C (Figure S6).[4] High-resolution N 1s (Figure S7a) and O 1s (Figure S7b) XPS show no shift in binding energies (within 0.2 eV, the instrument resolution) due to either strongly oxidizing or reducing atmospheres, indicating that the lattice nitrogen and oxygen in MCA lack the ability to directly participate in redox cycles (Table S1).[6,7] This is expected given that these atoms are covalently bonded to or within the triazin(an)e ring in the melamine and cyanuric acid monomers.

The XRD patterns and SEM images of MCA after 6 hr of each treatment are included in Figures S8 and S3b-f, respectively. The XRD patterns retain the same primary diffraction peaks as those in the parent MCA-as sample, indicating retention of long-range order despite strong oxidizing or reducing atmospheres and boiling solvents with mixing. SEM images demonstrate retention of the rod-like morphology as well, with unchanged length scales (~5-10 um long by ~0.5 um wide) for all treatments except toluene. The sample refluxed in toluene contains some smaller, ~1 um

features which likely form by interruption of π-π stacking interactions, rather than intralayer hydrogen bonds, due to the lack of competitive hydrogen-bond donors/acceptors in nonpolar toluene.[8] Still, long-range order is retained as indicated by XRD. Further, the mass recovery for all methods is >90% (Table S2), suggesting no significant volatilization or solubilization of the structure throughout the 6 hr treatments. While this is not an exhaustive set of catalytic environments, these results demonstrate a remarkable robustness of MCA despite its supramolecular nature. A complete analysis of the MCA stability under true catalytic conditions in the MP oxidative dehydrogenation reaction is included in Section 3.3.

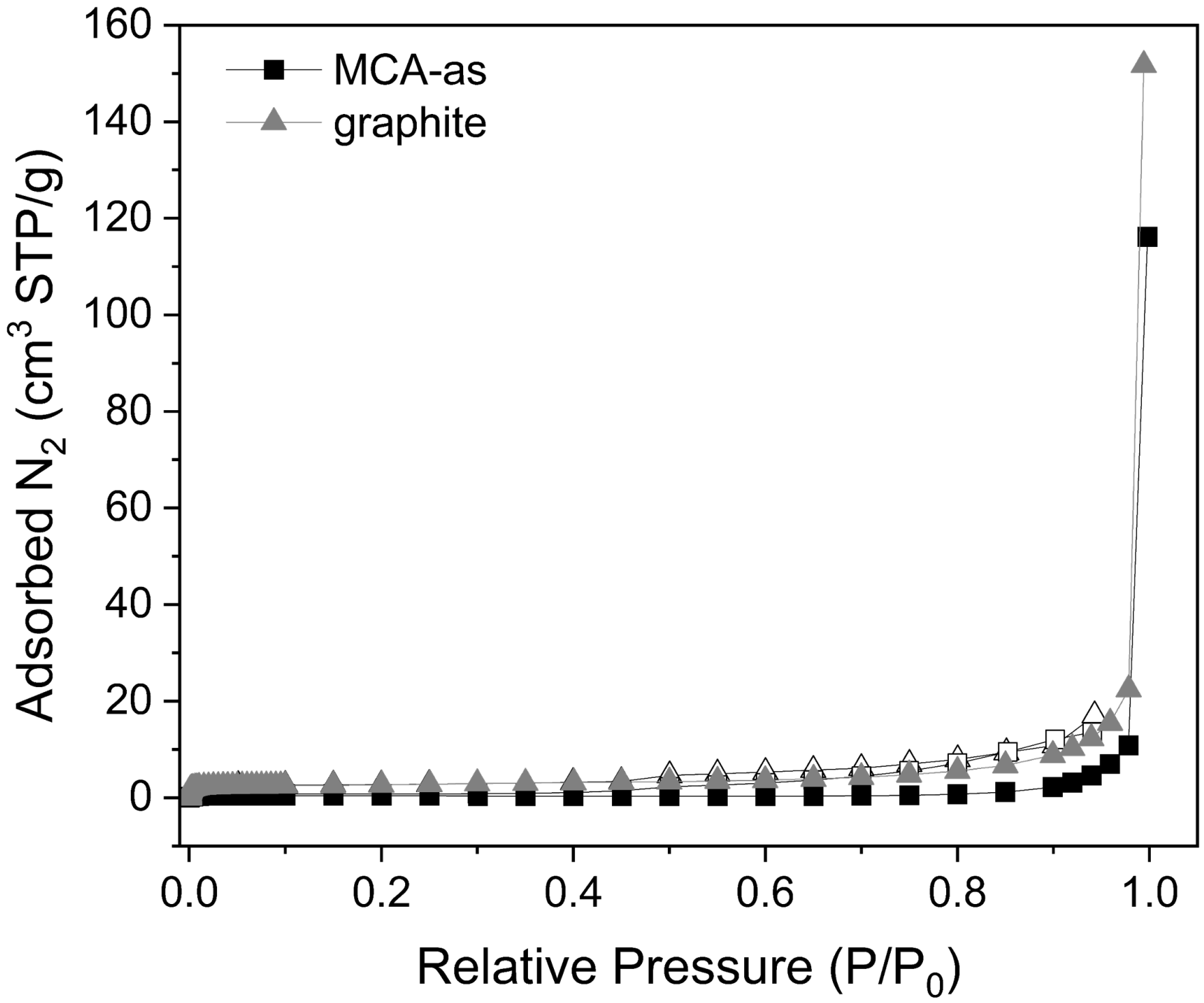

Figure S5. N_2 adsorption (closed symbols)-desorption (open symbols) isotherms collected at 77 K for MCA-as and graphite (as-received).

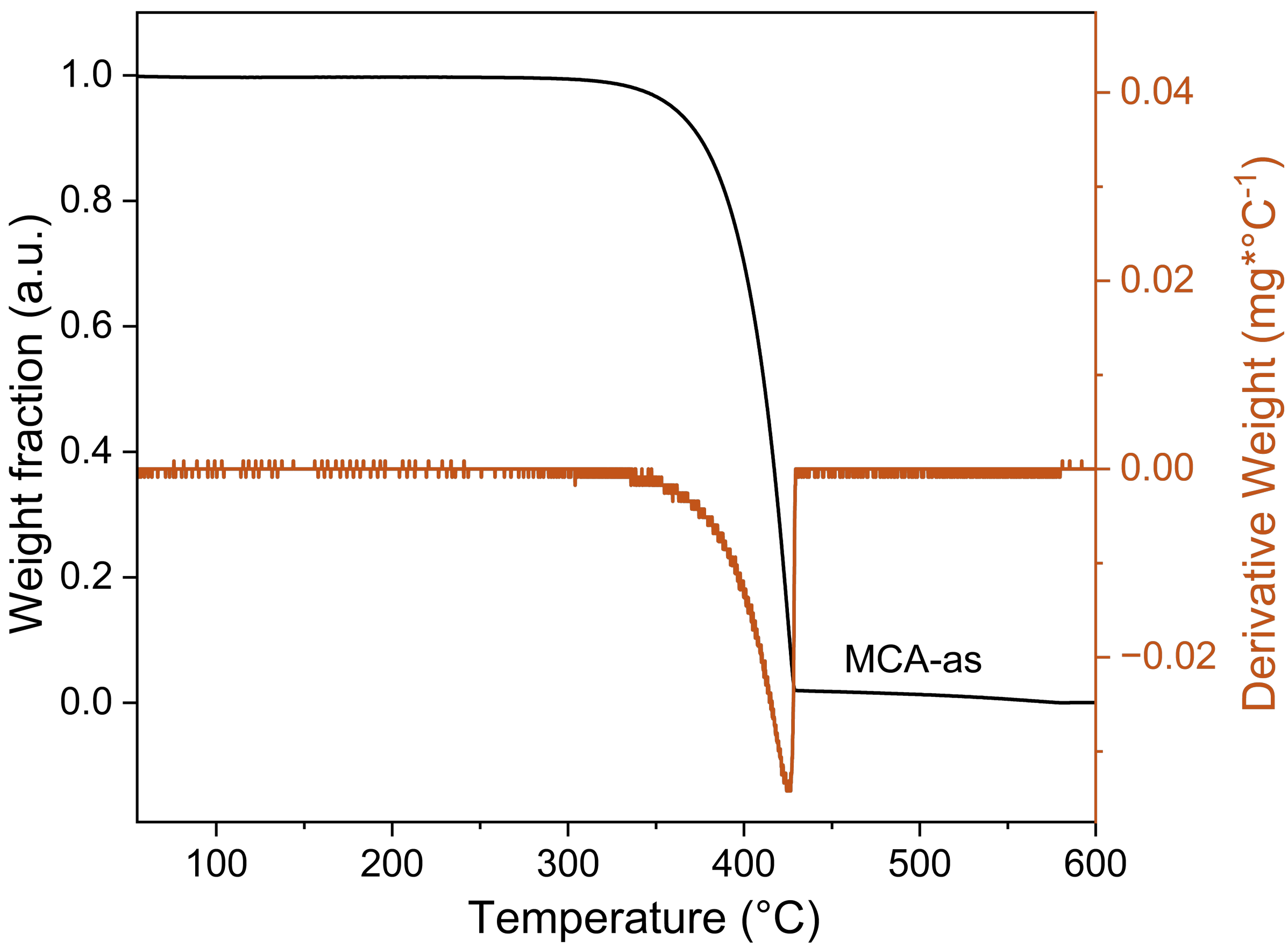

Figure S6. TGA profiles of MCA decomposition in air. Run conditions: 20 sccm air, 5 °C/min ramp rate from 30 °C to 800 °C.

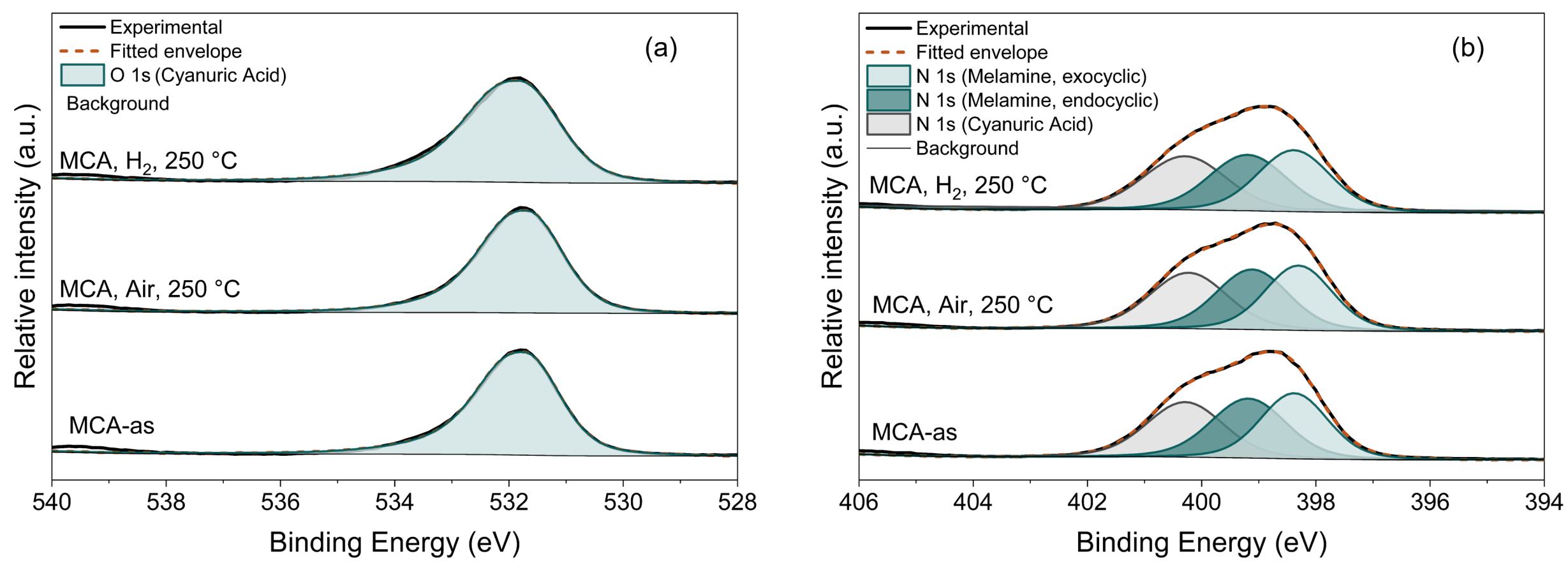

Figure S7. High-resolution XPS (a) O 1s spectra and (b) N 1s spectra of MCA before (-as) and after thermal treatment at 250 °C in either air or hydrogen for 6 hr.

Table S1. Binding energies in the O 1s and N 1s regions from XPS on MCA after thermal treatments.

MCA treatment	O 1s (eV)	N 1s, melamine exocyclic (eV)	N 1s, melamine endocyclic (eV)	N 1s, cyanuric acid (eV)
As-synthesized (-as)	531.7	398.4	399.2	400.3
Air, 250 °C, 6 hr	531.7	398.3	399.1	400.2
H_2, 250 °C, 6 hr	531.8	398.4	399.2	400.3

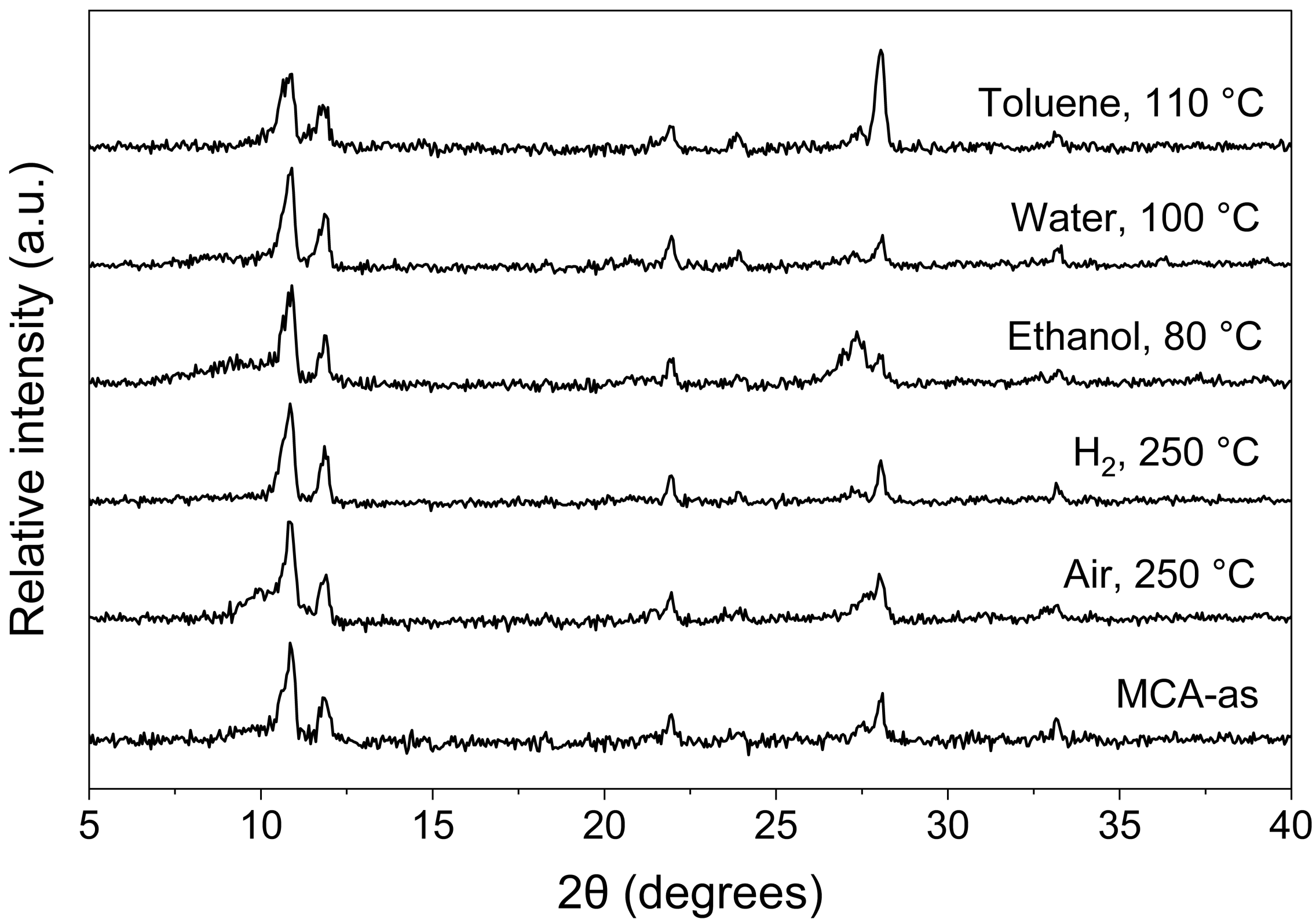

Figure S8. Powder XRD patterns of MCA subjected to different treatments. All treatments were carried out for 6 hr.

Table S2. Weight percentage recovery of MCA after various treatments, normalized to account for transfer losses.

Treatment	Wt.% Recovery
Air, 250 °C, 6 h	95.9
H_2, 250 °C, 6 h	100.4
Ethanol, 80 °C, 6 h	98.5
Water, 100 °C, 6 h	99.5
Toluene, 110 °C, 6 h	91.5

Table S3. Au loadings and NP diameters of selected catalysts as measured by ICP and TEM, respectively.

Sample	Au wt.%	Au NP diameter (nm)	$\langle d_{TEM} \rangle$ (nm) [DI]
Au-MCA, deposition	0.10	n/a	n/a
Au-MCA, deposition/reduction by $NaBH_4$	1.0	n/a	n/a
Au-PVA-MCA	0.89	2.5 +/- 0.5	2.7 [1.1]
Au-MTT-G	1.07	2.6 +/- 2.1	3.6 [1.5]
Au-P25 TiO_2	1.05	3.6 +/- 1.4	4.9 [1.4]

Deposition of Au onto MCA results in binding only one-tenth of the Au amount in solution to the support, as measured by ICP (Table S3). Introducing sodium borohydride as a strong reducing agent during deposition significantly enhances binding (reaching 100% Au utility, i.e., 1 wt.% loading), but gives rise to polydisperse Au NPs ranging from ~7 nm spheres to >40 nm aggregates (Figure S9a, TEM). This is analogous to what is observed on graphite using deposition and reduction by sodium borohydride (Figure S9b).[9] In principle, MCA has the potential for cation interactions with electron-rich sites (e.g., edge $-NH_2$ sites) on terminal melamine and cyanuric acid subunits, yet these do not coordinate significantly in practice. We attribute this to the weaker electron donation character of ring substituents (rather than, e.g., alkylamines) as well as the fact that the exposed edge sites on melamine and cyanuric acid are fully saturated in their coordination – a distinguishing feature of the facets of supramolecular materials – rather than defective terminations.

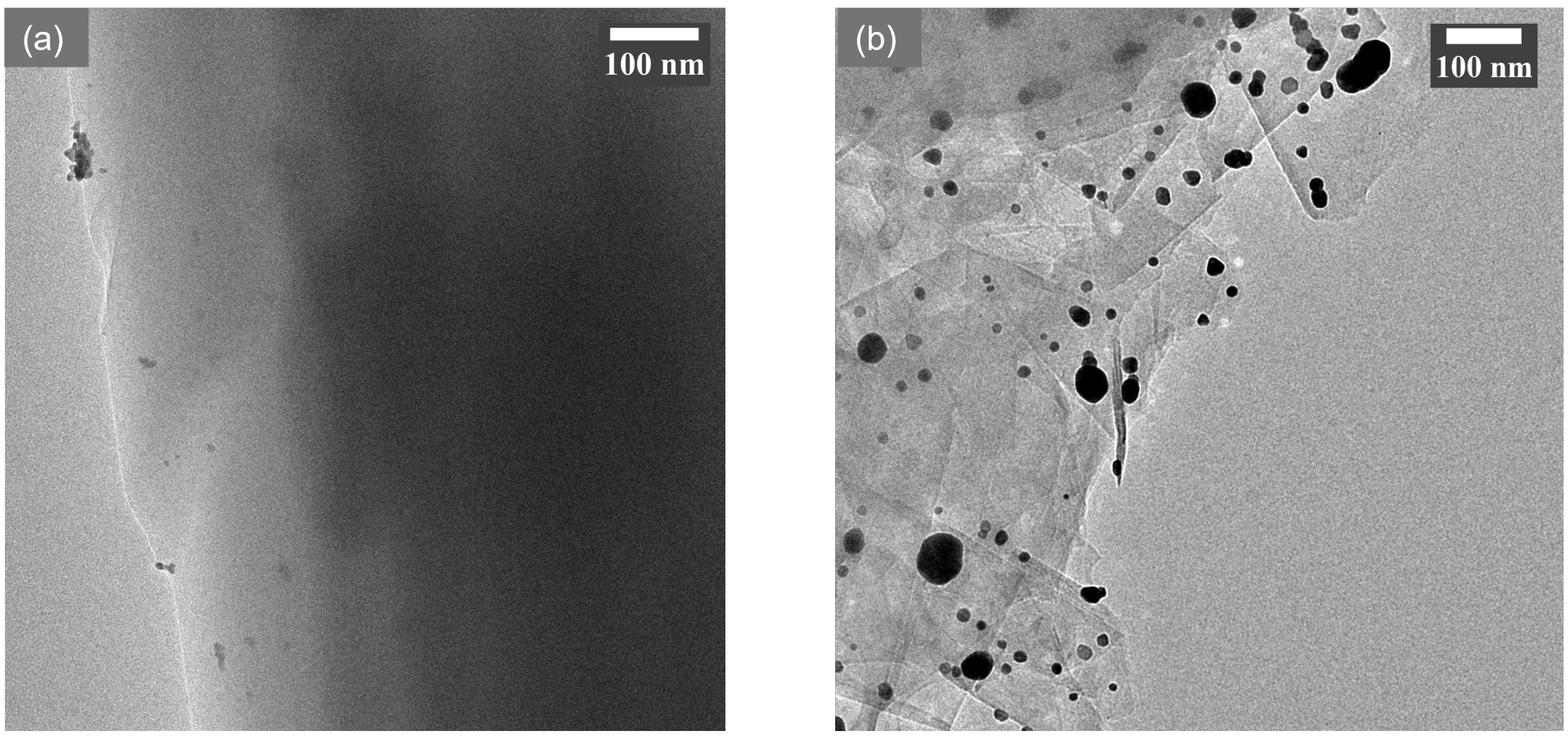

Figure S9. TEM images of Au loaded onto (a) MCA and (b) graphite by deposition and reduction with $NaBH_4$.

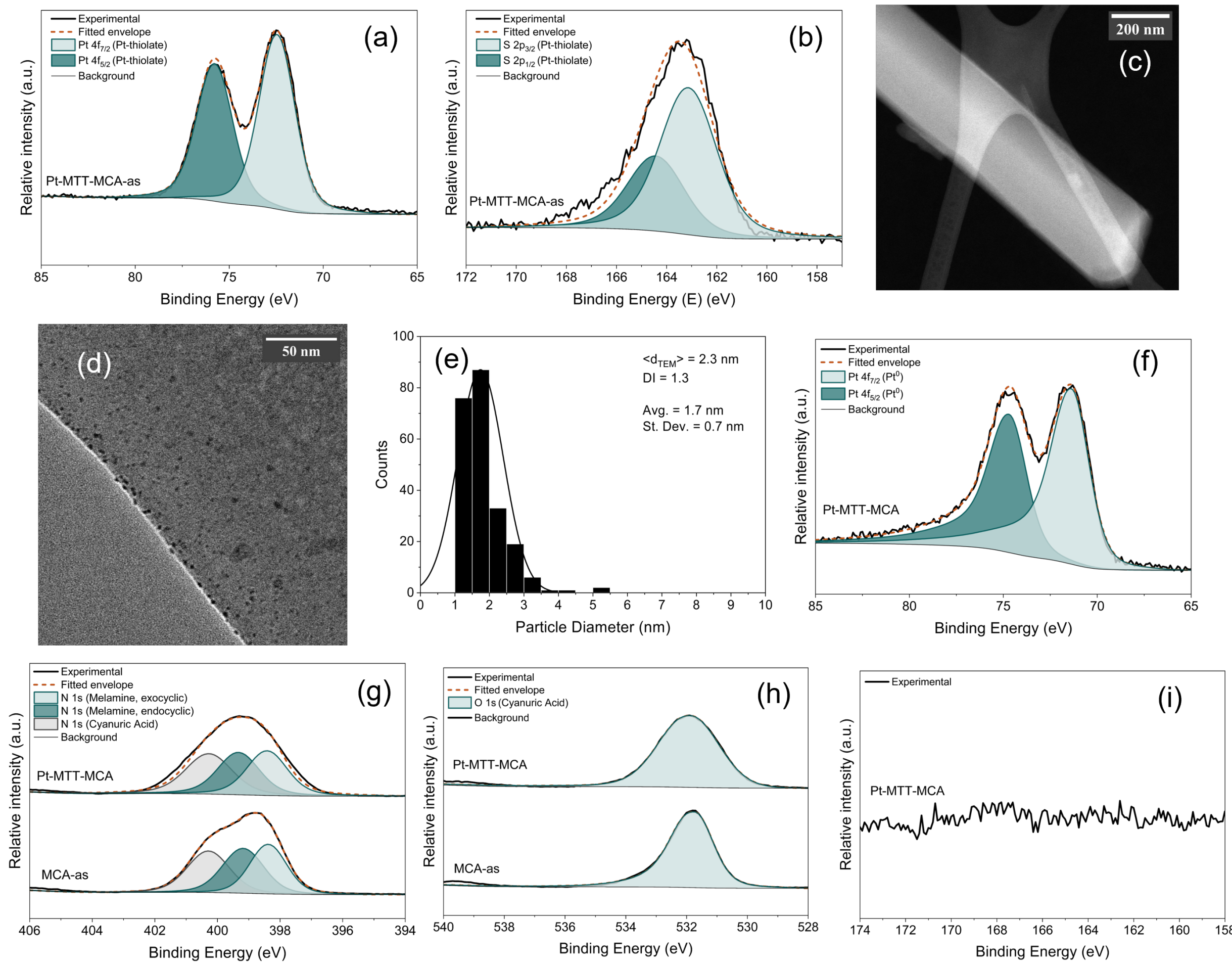

Figure S10: High resolution (a) Pt 4f and (b) S 2p XPS spectra of Pt-MTT-MCA-as. (c) High resolution TEM of Pt-MTT-MCA-as. (d) High resolution TEM image and (e) particle size distribution (PSD) of Pt-MTT-MCA. High resolution (f) Pt 4f, (g) N 1s, (h) O 1s, and (i) S 2p XPS spectra of Pt-MTT-MCA.

Analysis by high-resolution Pt 4f (Figure S10a) and S 2p (Figure S10b) XPS confirms the presence of Pt in a uniform oxidation state consistent with a Pt-thiol complex.[10] This, coupled with the lack of clusters and/or NPs observed in TEM (Figure S10c), suggests that Pt-MTT complexes highly disperse onto the MCA support by a similar molecular recognition mechanism as for Au. Upon thermal treatment (270 °C vs. 250 °C for Au), TEM imaging (Figure S10d) shows the formation of 1.7 +/- 0.7 nm Pt NPs with $\langle d_{TEM} \rangle$ = 2.3 nm, DI = 1.3 (Figure S10e). XPS in the Pt 4f regions shows the Pt 4f 7/2 signal (Figure S10f) at 71.5 eV, which can be attributed to very small (~2 nm or less) Pt^0 species.[11] Analysis of the N 1s (Figure S10g) and O 1s (Figure S10h) regions show no binding energy shifts (≤0.2 eV, the instrument resolution) relative to MCA-as, indicating no measurable electron transfer from/to MCA to the Pt NPs at the Pt^0 NP/MCA interface. While the S 2p signal is absent after thermal treatment (Figure S10i), ICP analysis indicates the presence of some residual S (0.03-0.04 wt.%). We attribute this to a stronger Pt-S interaction that precludes full cleavage at this temperature. These findings indicate that the molecular recognition-directed dispersion underlying this strategy extends to the Pt-MTT system, while complete removal of the

bridge depends on the M-bridge pairing. Thus, M-bridge interactions must be carefully designed to ensure selective, high dispersion along with NP liberation from the bridge molecule.

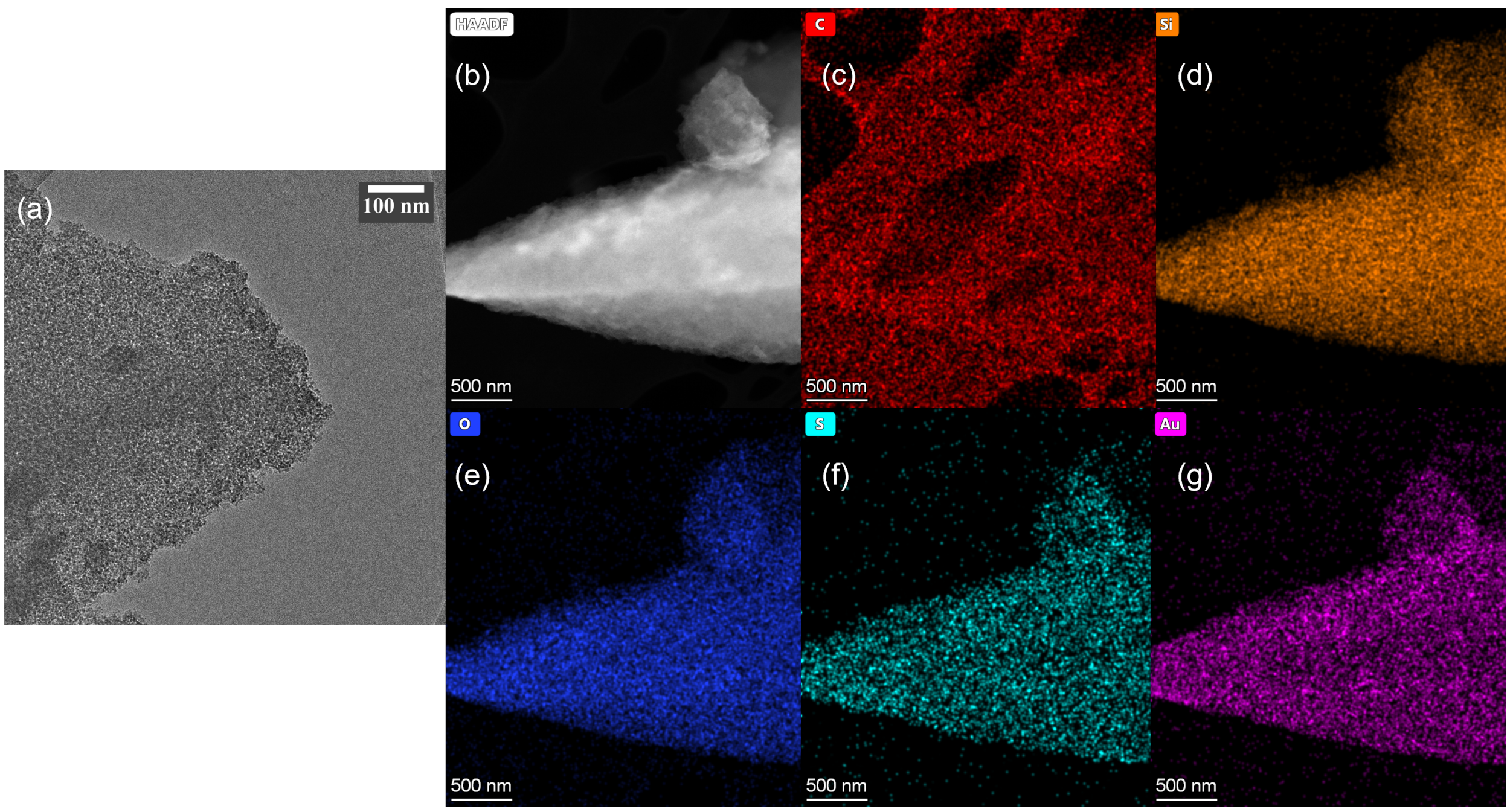

Figure S11. (a) TEM and (b) HAADF-STEM images with (c-g) EDS analysis on Au-MPTMS-SiO_2-as.

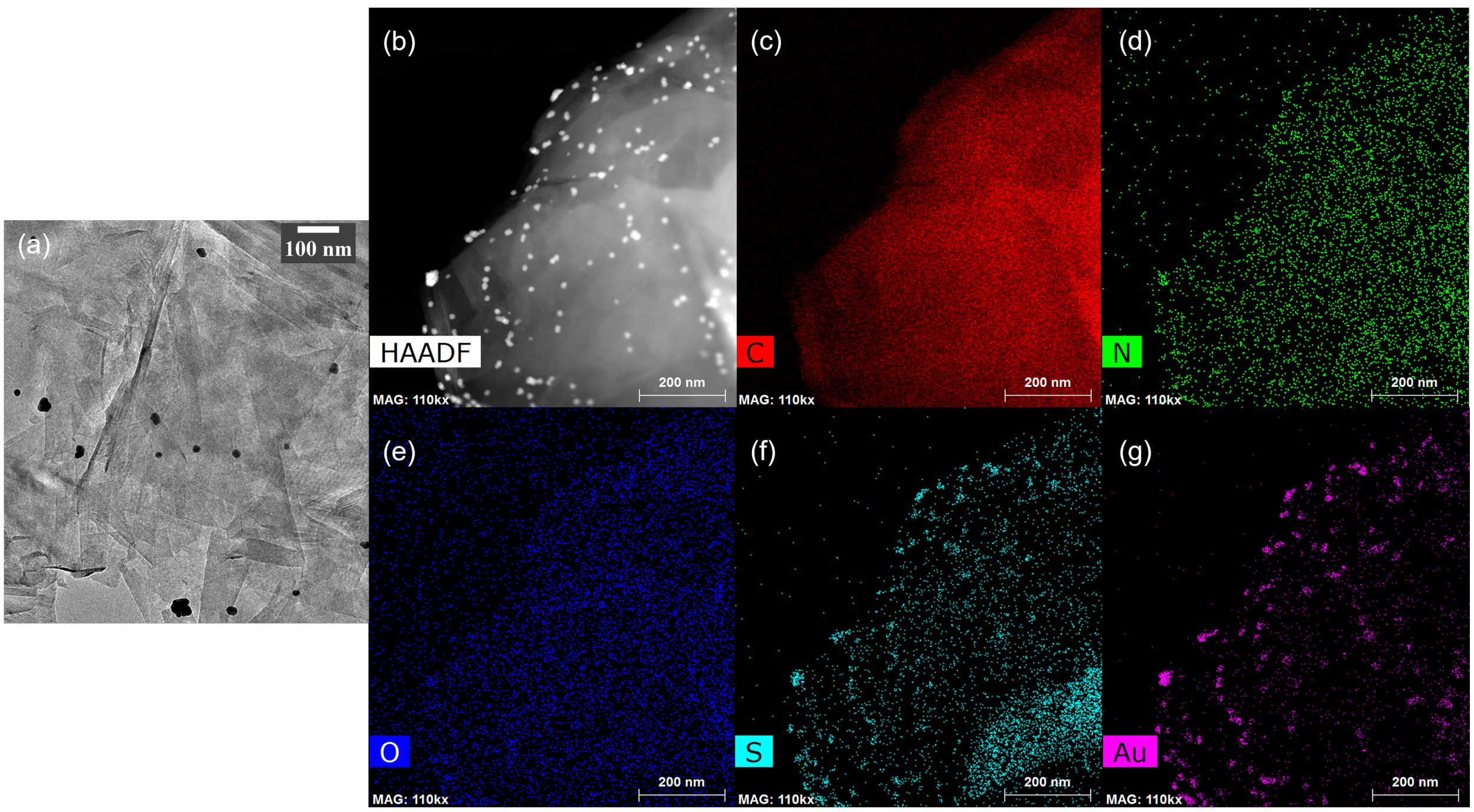

Figure S12. (a) TEM and (b) HAADF-STEM images with (c-g) EDS analysis on Au-MTT-G-as.

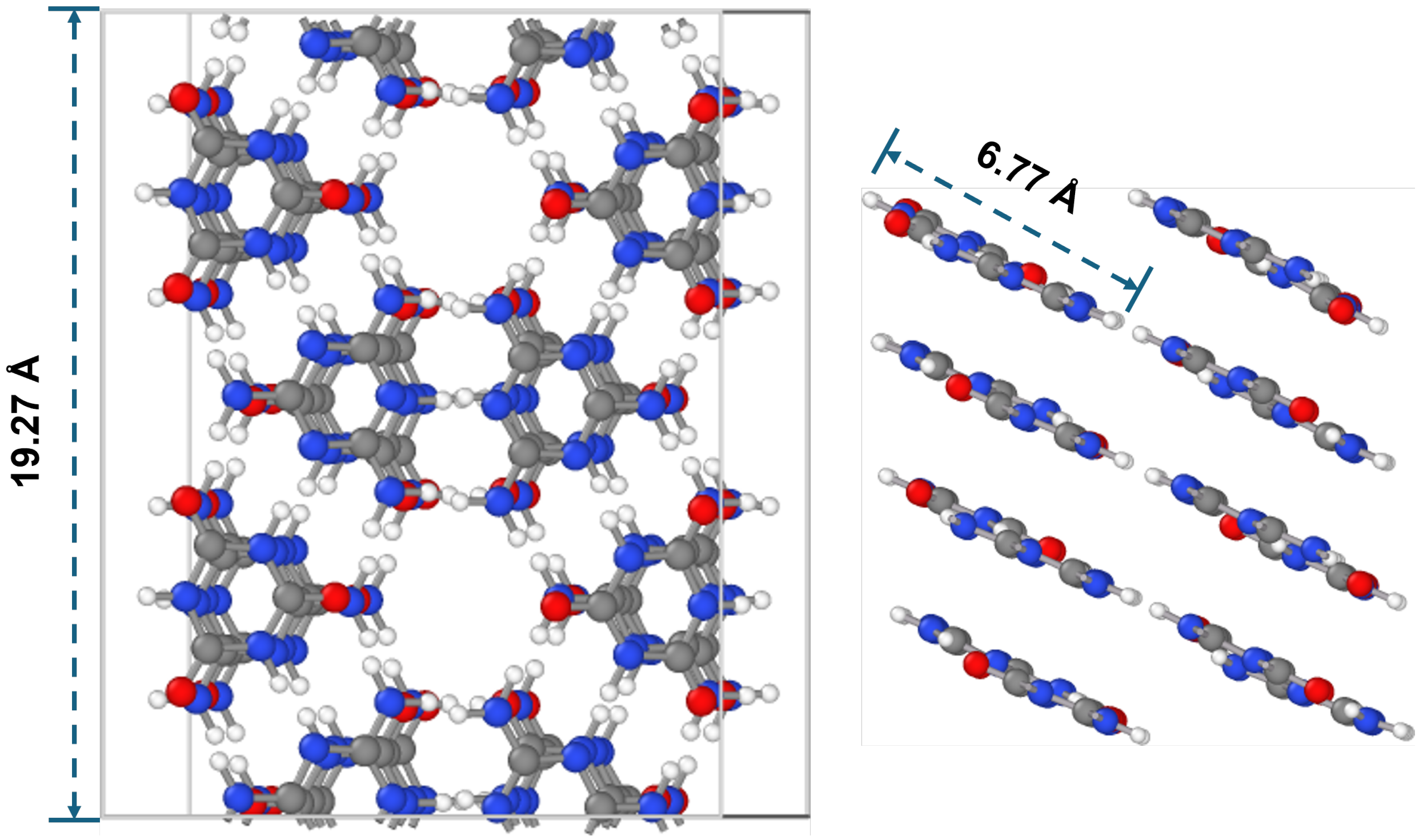

Figure S13. Top view and side profile of stepped MCA surface with dimensions of each step.

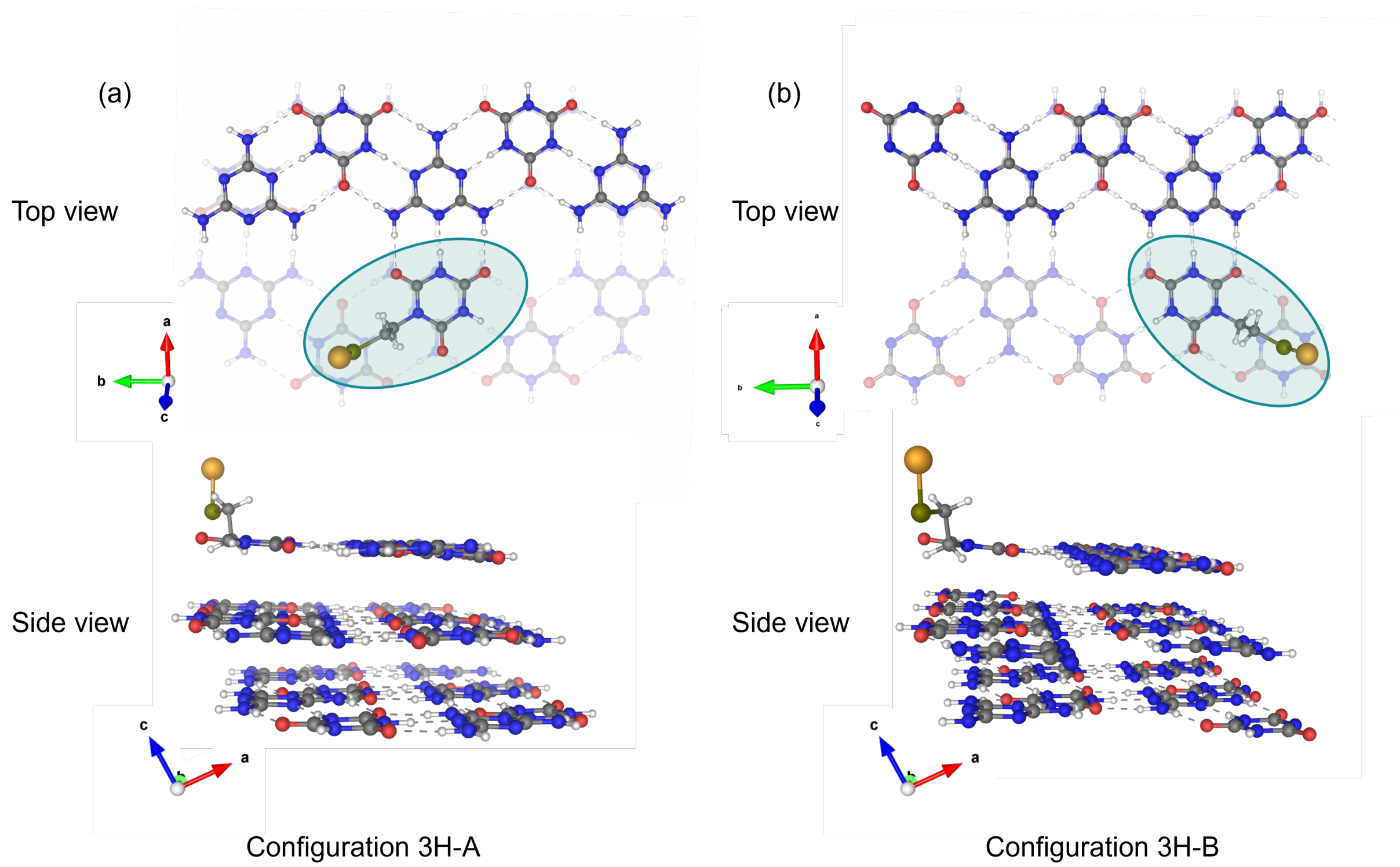

Figure S14. Top and side views of geometry optimized Au-MTT coordinations to MCA with 3 hydrogen bonds, with the cyanuric acid backbone of MTT coordinating to the melamine in MCA. The two distinct coordinations arise when the Au-thiol arm is facing (a) in the $+\hat{b}$ (3H-A) and (b) in the $-\hat{b}$ (3H-B) directions.

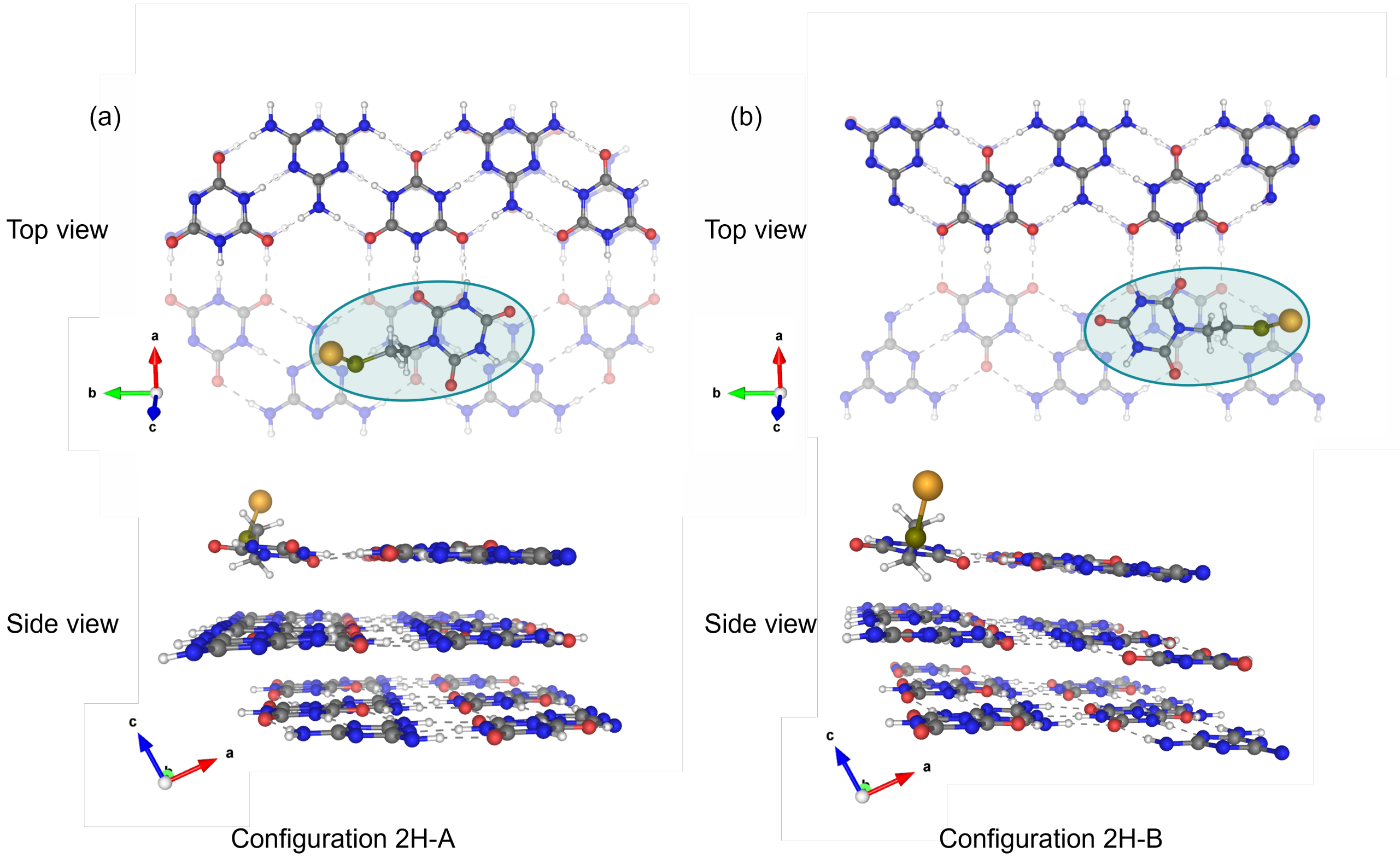

Figure S15. Top and side views of geometry optimized Au-MTT coordinations to MCA with 2 hydrogen bonds, with the cyanuric acid backbone of MTT coordinating to the cyanuric acid in MCA. The two distinct coordinations arise when the Au-thiol arm is facing (a) in the $+\hat{b}$ (2H-A) and (b) in the $-\hat{b}$ (2H-B) directions.

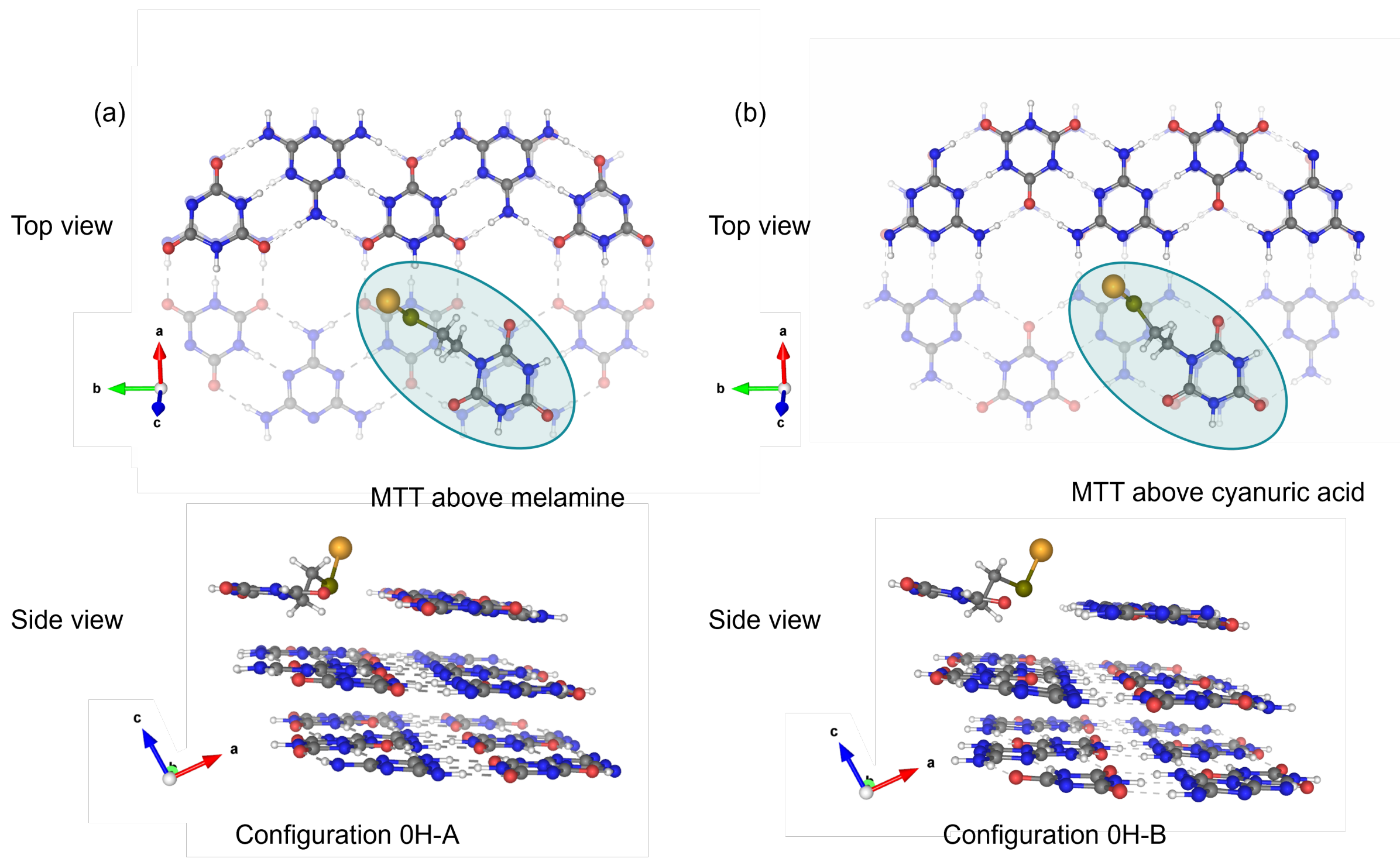

Figure S16. Top and side views of geometry optimized Au-MTT coordination to MCA with 0 hydrogen bonds, containing only nonspecific π- π interactions above the MCA basal planes. The two distinct coordinations arise when the MTT is centered above (a) melamine (0H-A) and (b) cyanuric acid (0H-B).

Table S4. Different Au-MTT coordination configurations and adsorption energies.

Support	***Au-MTT ⋯support configuration code***	***Adsorption Energy* (eV)**	***Interactions***
MCA	3H-A	-1.16	π-π stacking, 3 hydrogen bonds, Au-S head facing $+\hat{b}$
	3H-B	-1.13	π-π stacking, 3 hydrogen bonds, Au-S head facing $-\hat{b}$
	2H-A	-1.05	π-π stacking, 2 hydrogen bonds, Au-S head facing $+\hat{b}$
	2H-B	-0.99	π-π stacking, 2 hydrogen bonds, Au-S head facing $-\hat{b}$
	0H-A	-0.79	π-π stacking only (above melamine)
	0H-B	-0.56	π-π stacking only (above cyanuric acid)
graphite	0H-C	-0.79	coordinated above graphite basal planes, π-π stacking only

The configurations containing three hydrogen bonds to MCA are most energetically favorable, followed by the ones containing two hydrogen bonds. The energies of these respective configurations are similar among the A and B coordinations due to the periodic nature of MCA. The discrepancy between 0H-A and 0H-B arises from more favorable stacking interactions between melamine-cyanuric acid pairs when compared to cyanuric acid-cyanuric acid pairs, as reported by Prior et al.[12]

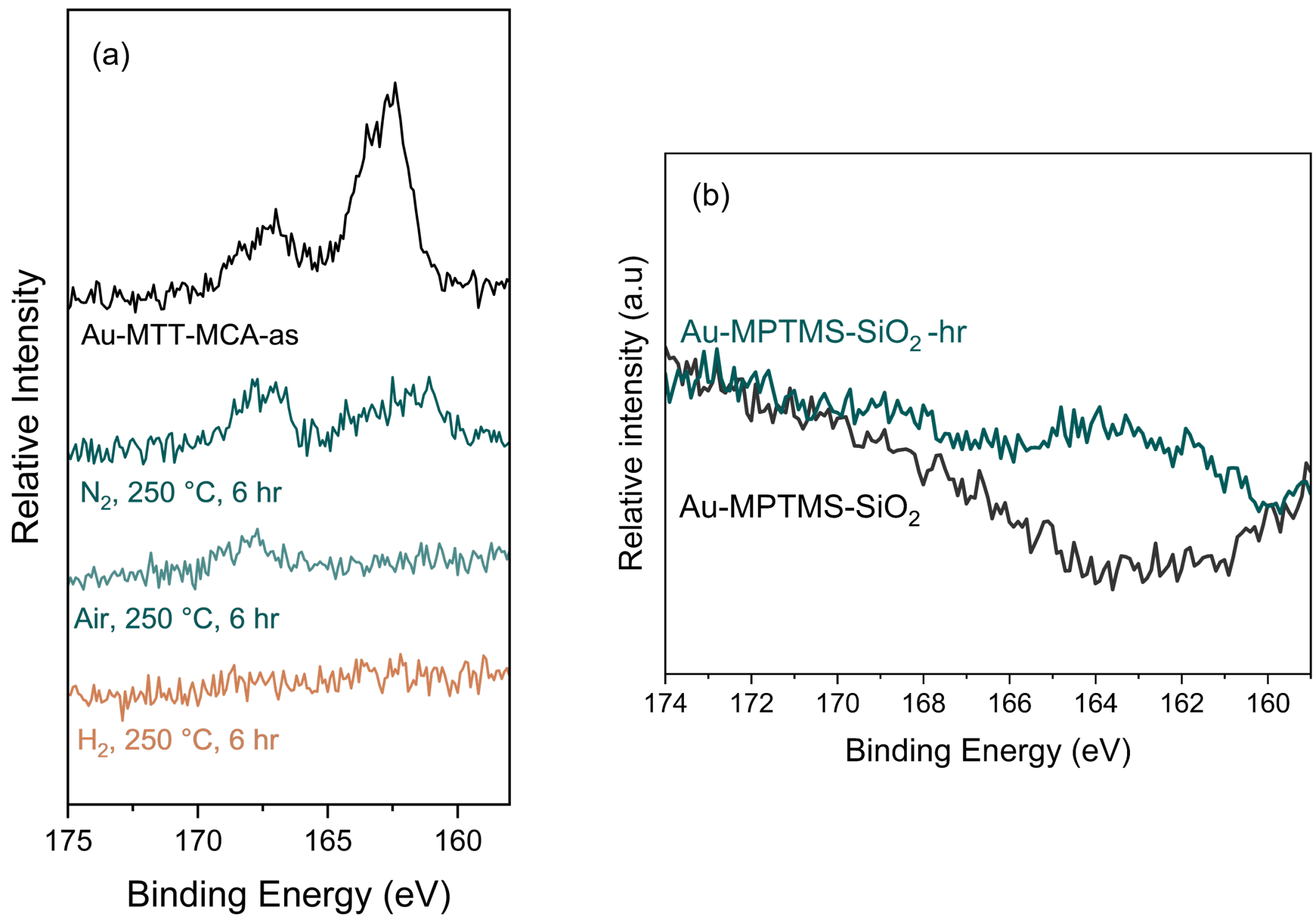

Figure S17. High-resolution S 2p XPS spectra of (a) Au-MTT-MCA-as before and after annealing in in N_2, air, and H_2 for 6 hr at 250 °C and (b) Au-MPTMS-SiO_2 calcined in air (550 °C) and H_2 (250 °C, "-hr"). Since the milder conditions were not sufficient to remove sulfur from Au-MPTMS-SiO_2-as, further characterization and catalysis proceeded on the air-calcined sample. The trailing peak around 158 eV followed by a local minimum around 163 eV present on the silica samples is an artifact from the Si 2s peak.[13]

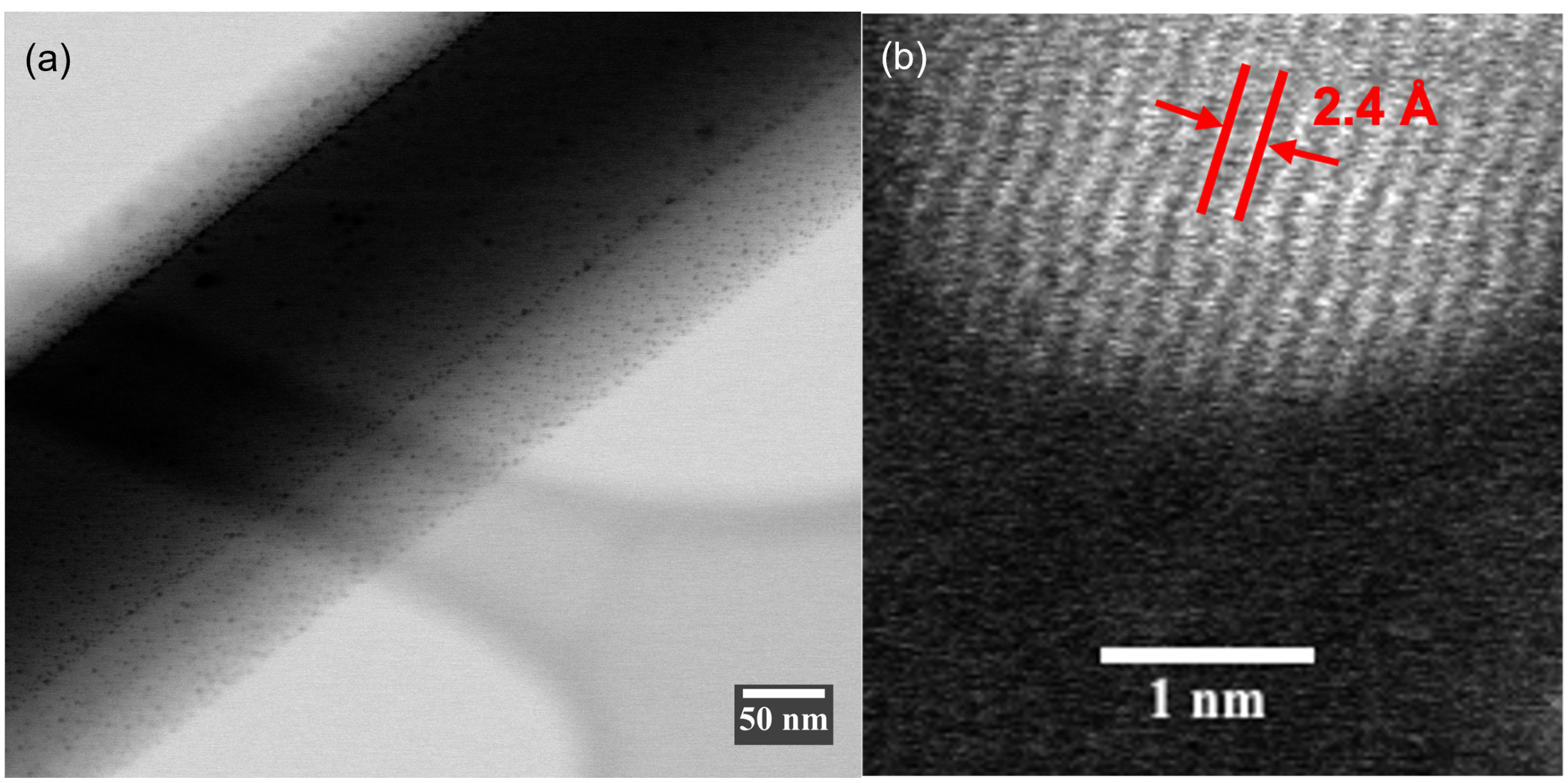

Figure S18. (a) Bright-field STEM image of Au-MTT-MCA. (b) Au nanoparticle lattice fringes visualized by HAADF-STEM show d-spacing of 2.4 Å on Au-MTT-MCA.

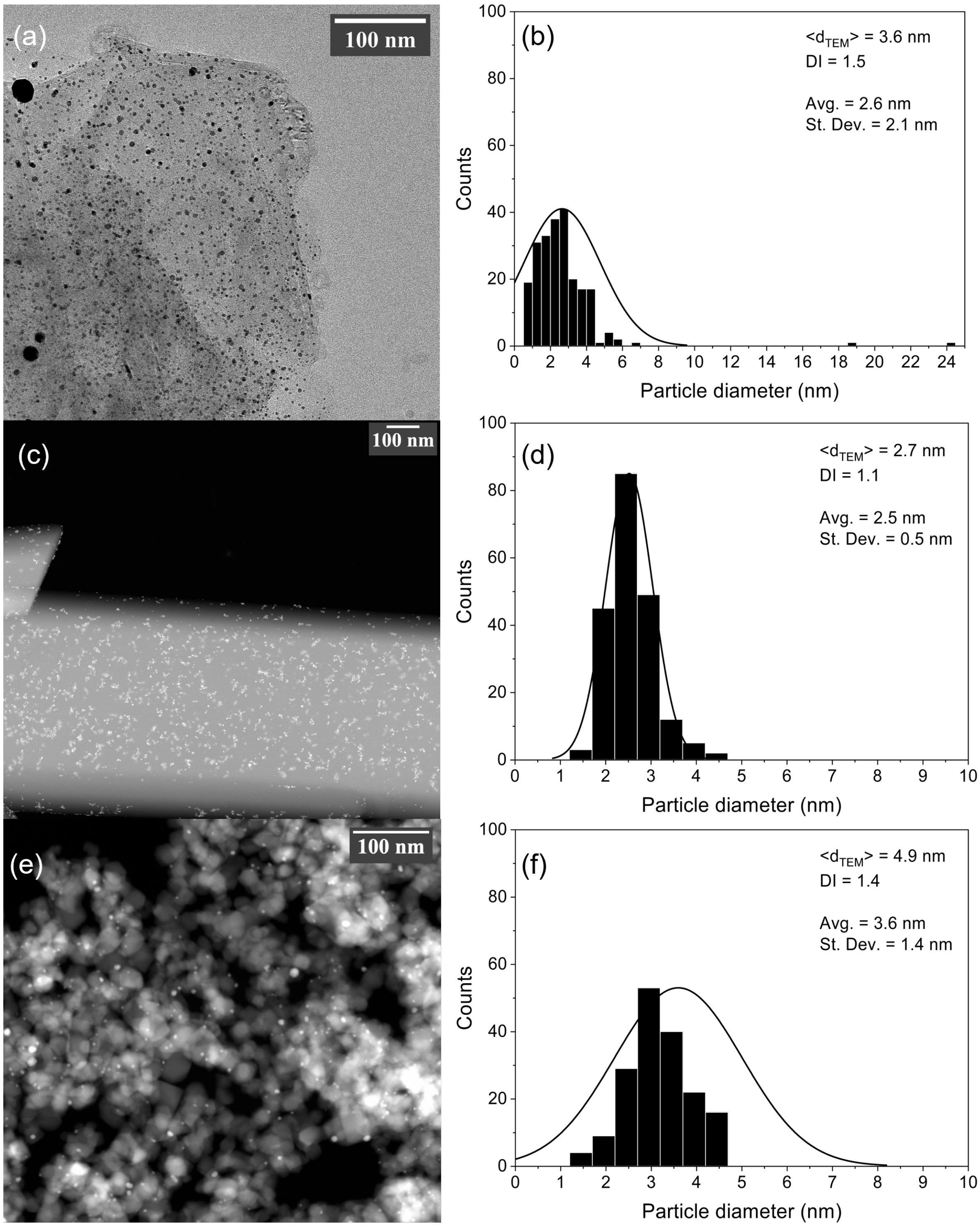

Figure S19. (a) TEM image and (b) PSD of Au-MTT-G. (c) HAADF-STEM image and (d) PSD of Au-PVA-MCA. (e) HAADF-STEM image and (f) PSD of Au-P25 TiO_2.

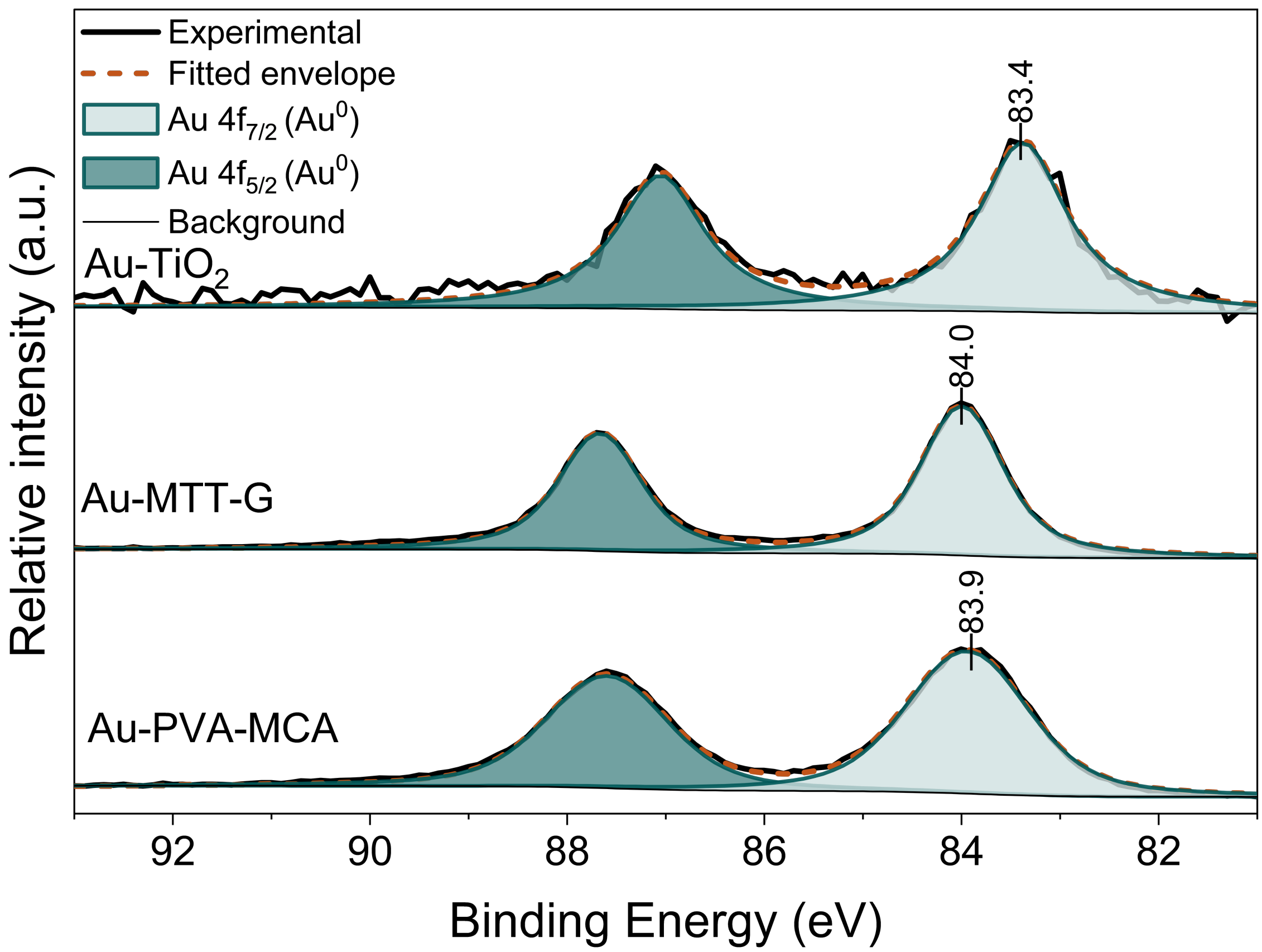

Figure S20. High resolution Au 4f XPS spectra of Au-PVA-MCA, Au-MTT-G, and Au-TiO_2.

Table S5. Full width at half maximum (FWHM) of fitted high resolution O1s and N1s XPS peaks comparing MCA-as and -hr with the functionalized Au-MTT-MCA catalyst.

Sample	O 1s FWHM (eV)	N 1s, melamine FWHM exocyclic (eV)	N 1s, melamine endocyclic FWHM (eV)	N 1s, cyanuric acid FWHM (eV)
MCA-as	1.7	1.4	1.6	1.6
MCA-hr	2.0	1.3	1.3	1.5
Au-MTT-MCA	2.1	1.7	2.0	2.0

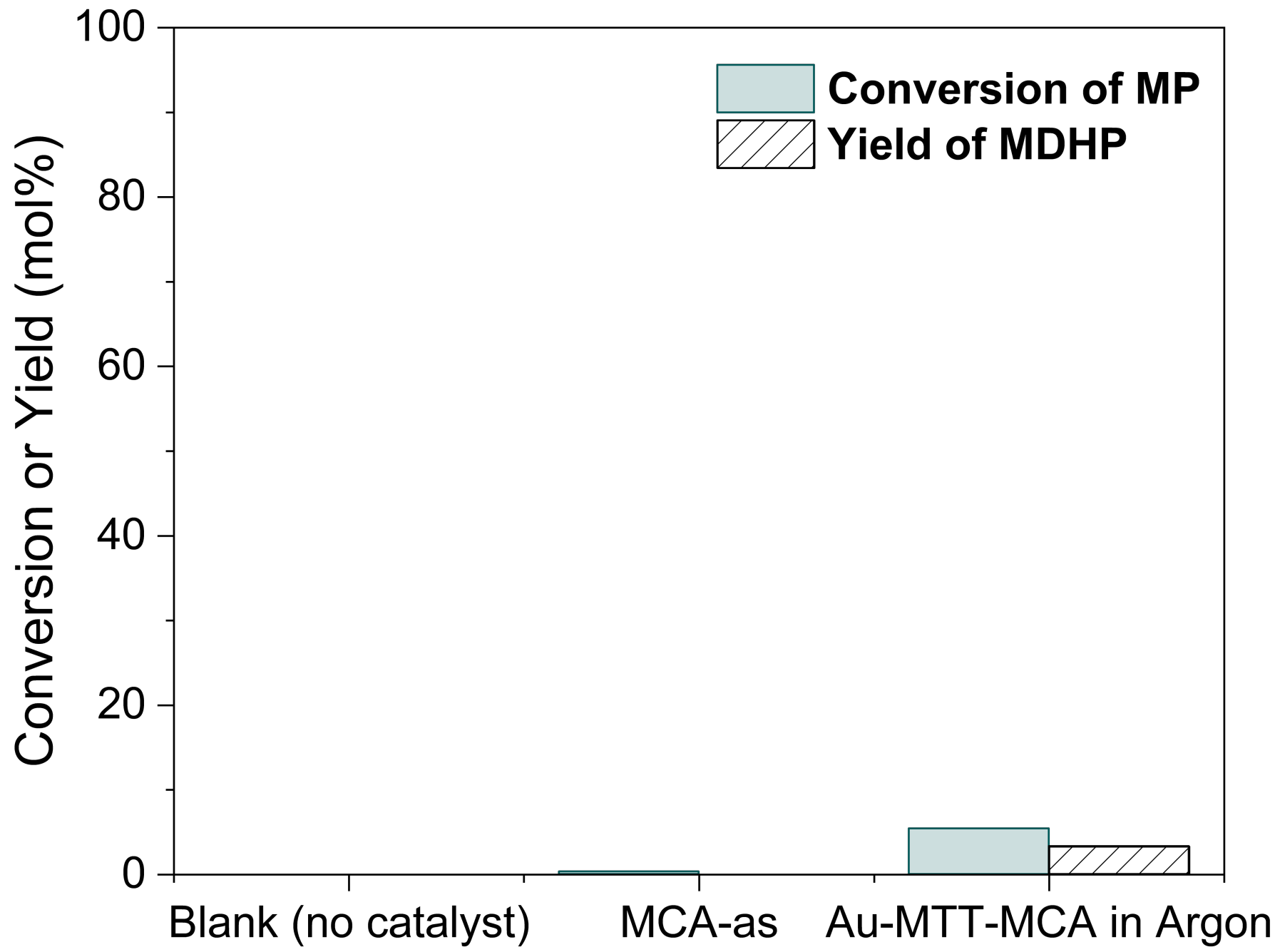

Figure S21. Control catalyst experiments. Typical run conditions (0.1 mmol MP, 0.1 mmol NMP (internal standard), 2 mL DI H_2O, 1 bar O_2, 50 mg catalyst, 60 °C, 6 hr) were modified depending on the run. In the blank, no catalyst was added. In the MCA-as run, as-synthesized, Au-free MCA was added. In the Argon run, Argon gas was bubbled through the headspace instead of oxygen to create a 1 bar Ar atmosphere. The activity in the Argon control can be attributed to a small amount of residual dissolved oxygen.

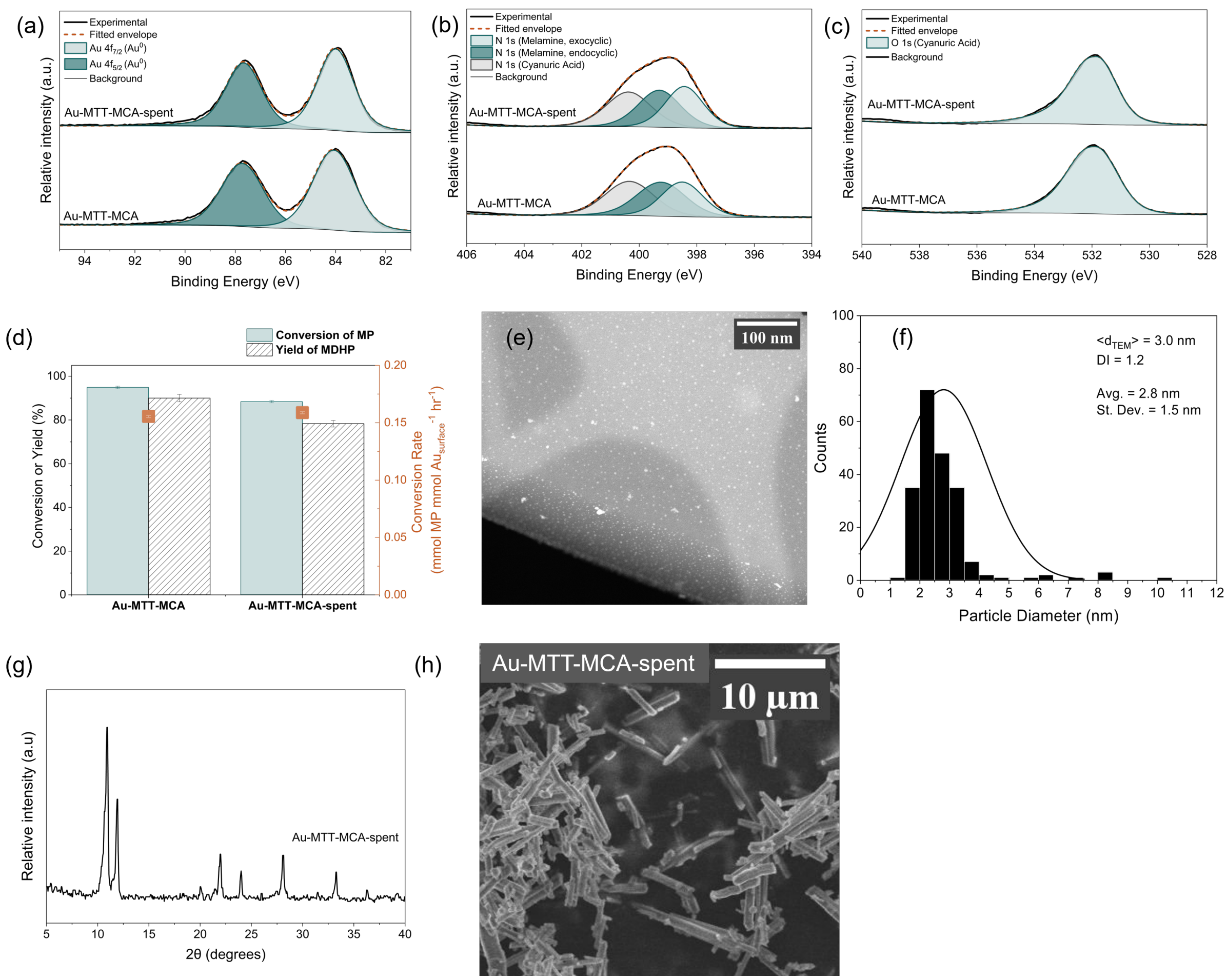

Figure S22. High resolution (a) Au 4f, (b) N 1s, and (c) O 1s XPS spectra of Au-MTT-MCA before and after catalysis (-spent). The catalyst was run under typical reaction conditions for 6 hr, filtered, washed with water, and dried at 80 °C, yielding Au-MTT-MCA-spent, which was then run in the recycle run. (d) Conversion, MDHP yield, and conversion rate of the initial and recycle runs. (e) High resolution STEM image and (f) particle size distribution of Au-MTT-MCA-spent. (g) Powder XRD patterns and (h) SEM images of Au-MTT-MCA after a catalytic run.

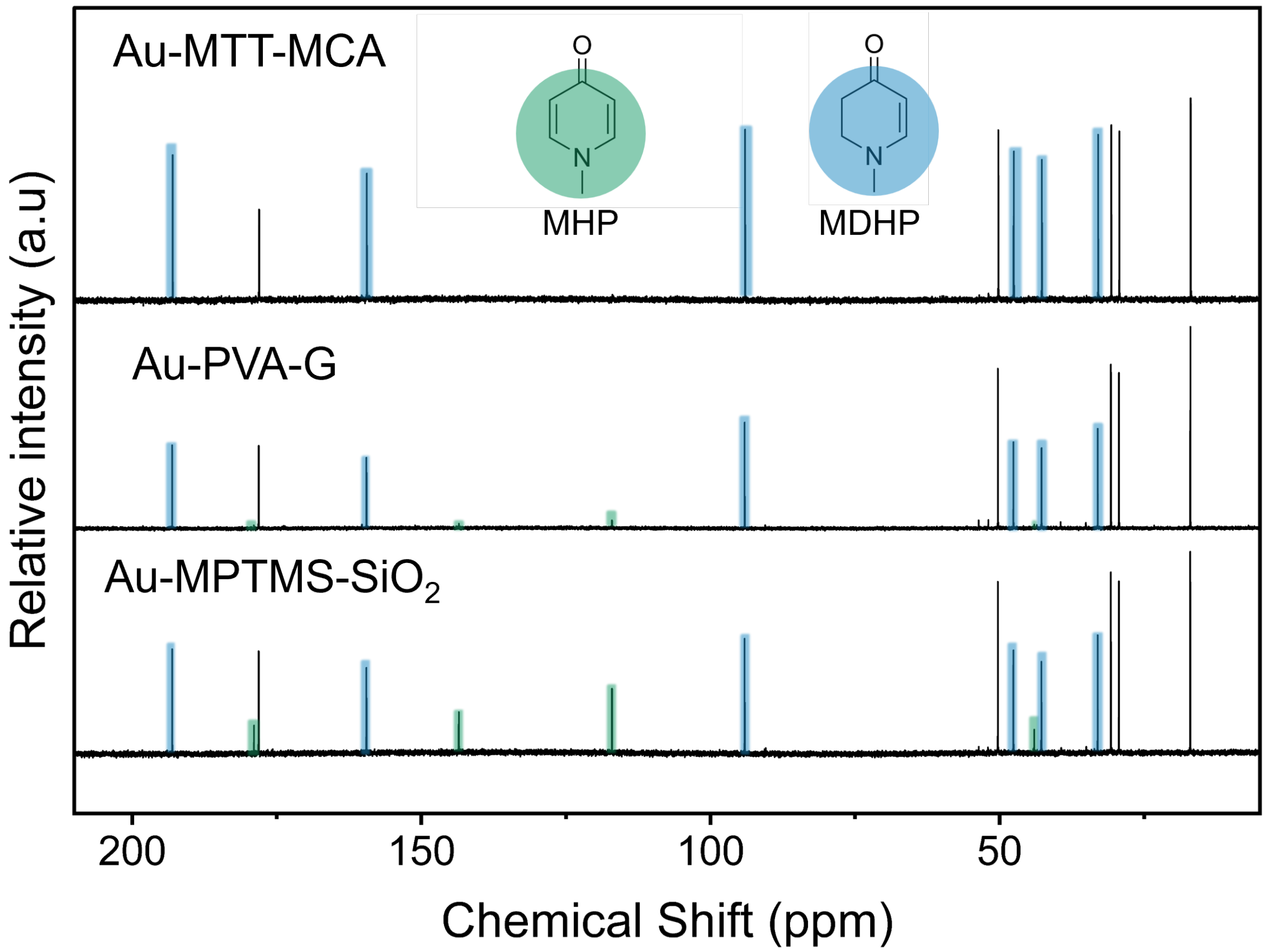

Figure S23. ^{13}C NMR spectroscopy spectra of reaction mixtures run for 6 hr. The two products, MDHP and MHP, are labeled with colors corresponding to the distinct carbons present in each sample. ^{13}C NMR in 90:10 H_2O:D_2O δ (ppm) MDHP (blue): 33, 43, 48, 94, 160, 193;[14] MHP (green): 179, 143 (2C), 117 (2C), 44.[15] Uncolored peaks correspond to NMP, the internal standard.

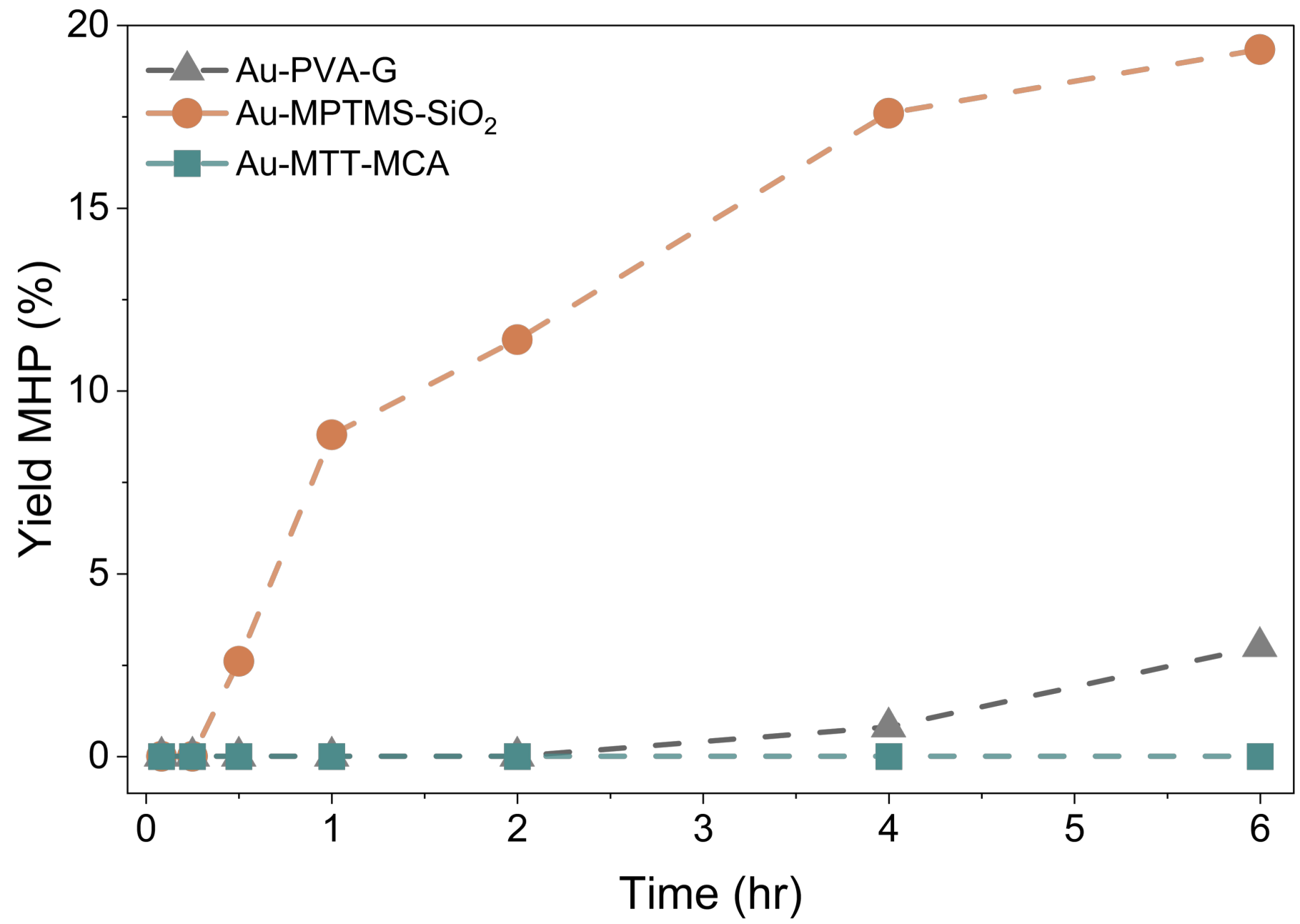

Figure S24. Time resolved yield of MHP for three catalysts. Typical run conditions were used: 0.1 mmol MP, 0.1 mmol NMP (internal standard), 2 mL DI H_2O, 1 bar O_2, 50 mg catalyst, 60 °C with variable time points.

Table S6. Approximate site fractions on Au NPs, assuming a truncated cuboctahedral model.[16,17]

Catalyst	Au NP <d_{TEM}> (nm)	Terrace Fraction (%)	Edge Fraction (%)	Corner Fraction (%)
Au-PVA-G	2.5	73	21	6
Au-MTT-MCA	2.7	75	20	5
Au-MPTMS-SiO_2	3.8	82	16	3

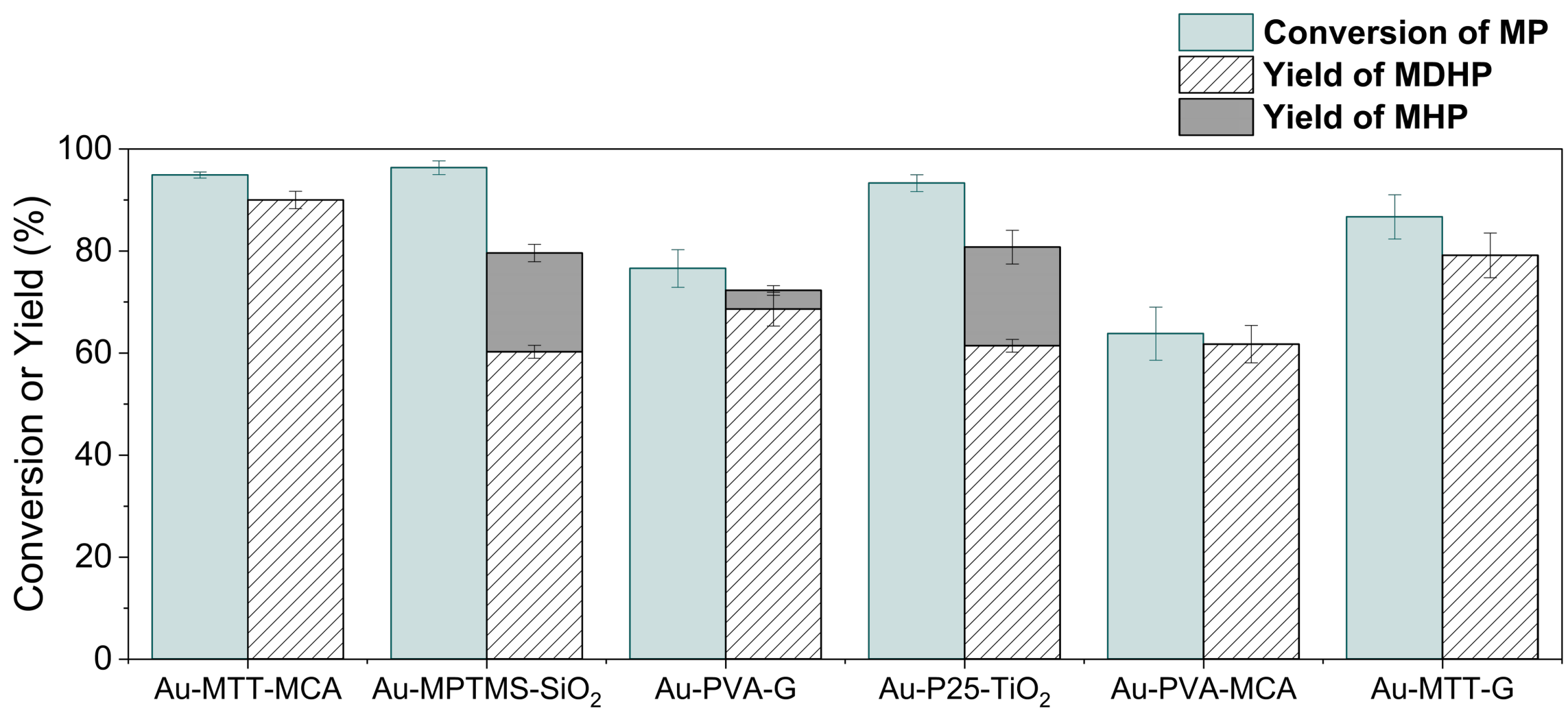

Figure S25. Conversion and yield of all catalyst samples at 6 hr. Run conditions: 0.1 mmol MP, 0.1 mmol NMP (internal standard), 2 mL DI H_2O, 1 bar O_2, 50 mg catalyst, 60 °C, 6 hr.

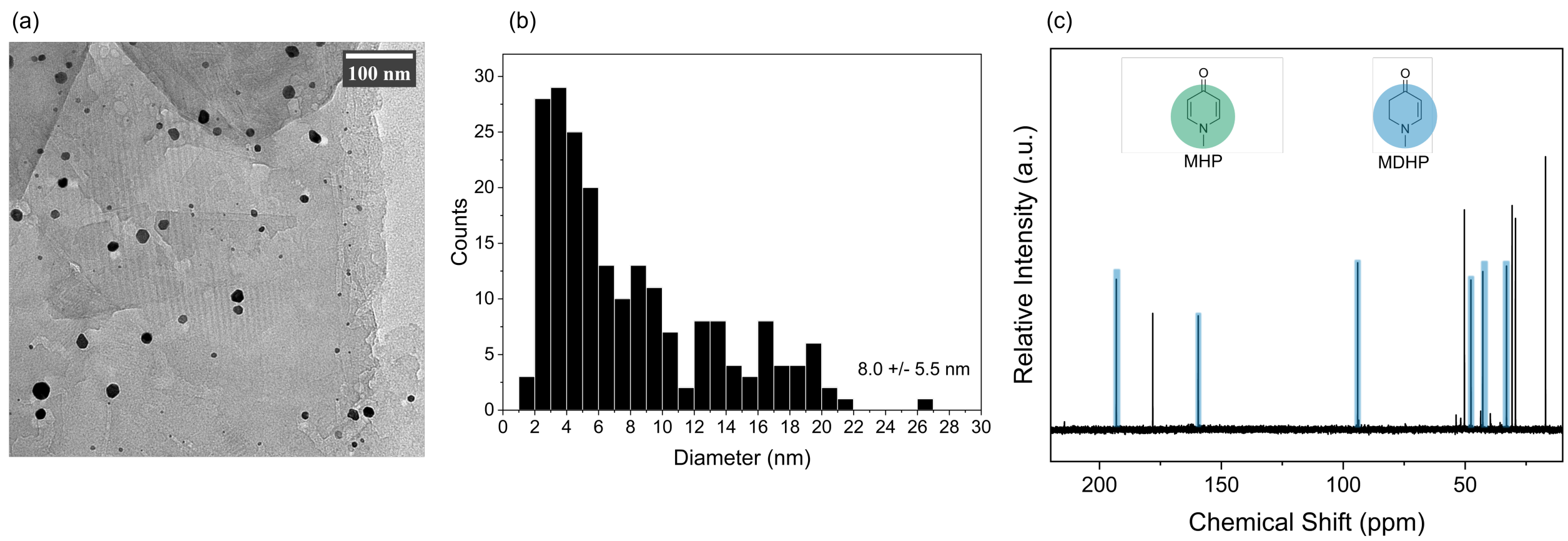

Figure S26. (a) TEM image and (b) particle size distribution of calcined Au-PVA-G. (c) ^{13}C NMR spectroscopy spectra of a calcined Au-PVA-G reaction mixture run for 6 hr. Unlabeled peaks (major) correspond to NMP, the internal standard, and unreacted MP (minor).

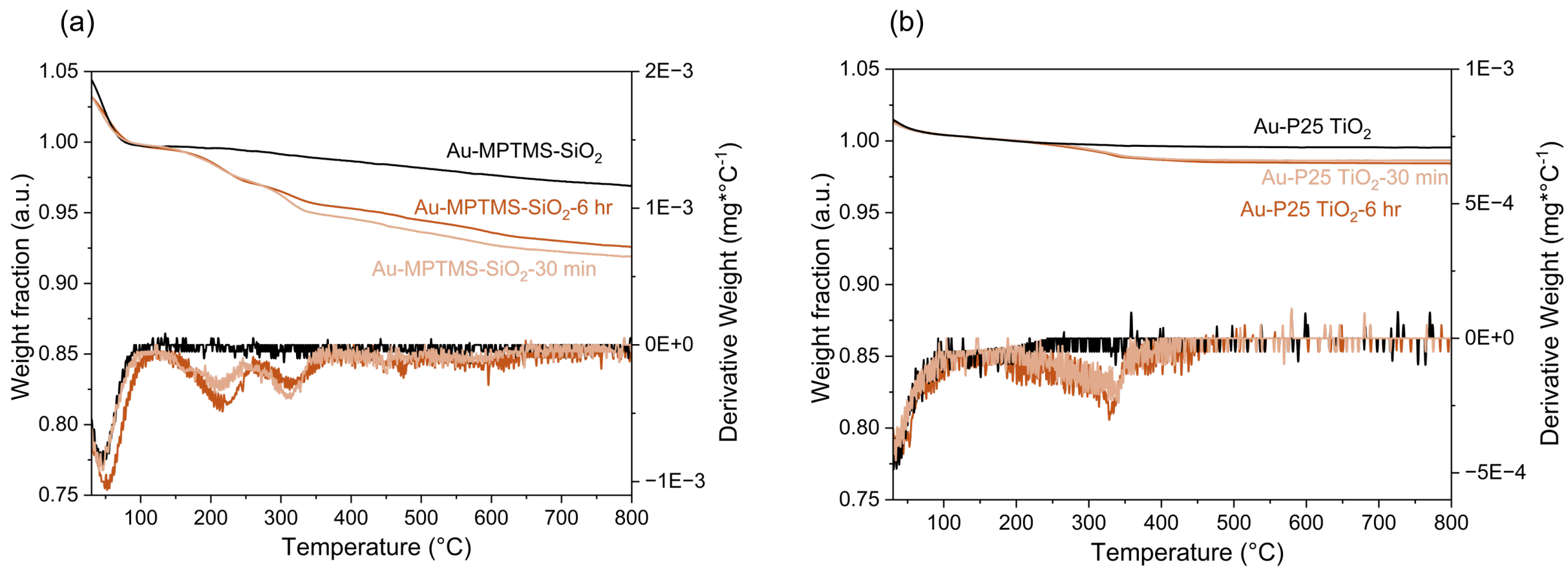

Figure S27. TGA of (a) Au-MPTMS-SiO_2 and (b) Au-P25-TiO_2 before and after reaction for 30 minute and 6 hour runs. Run conditions: 20 sccm air, 5 °C per minute ramp from 30 °C to 800 °C. Weight fraction is plotted on the upper half and derivative weight is plotted on the bottom half. Weight fraction was normalized to the dried catalyst mass at 100 °C for comparison of the samples.

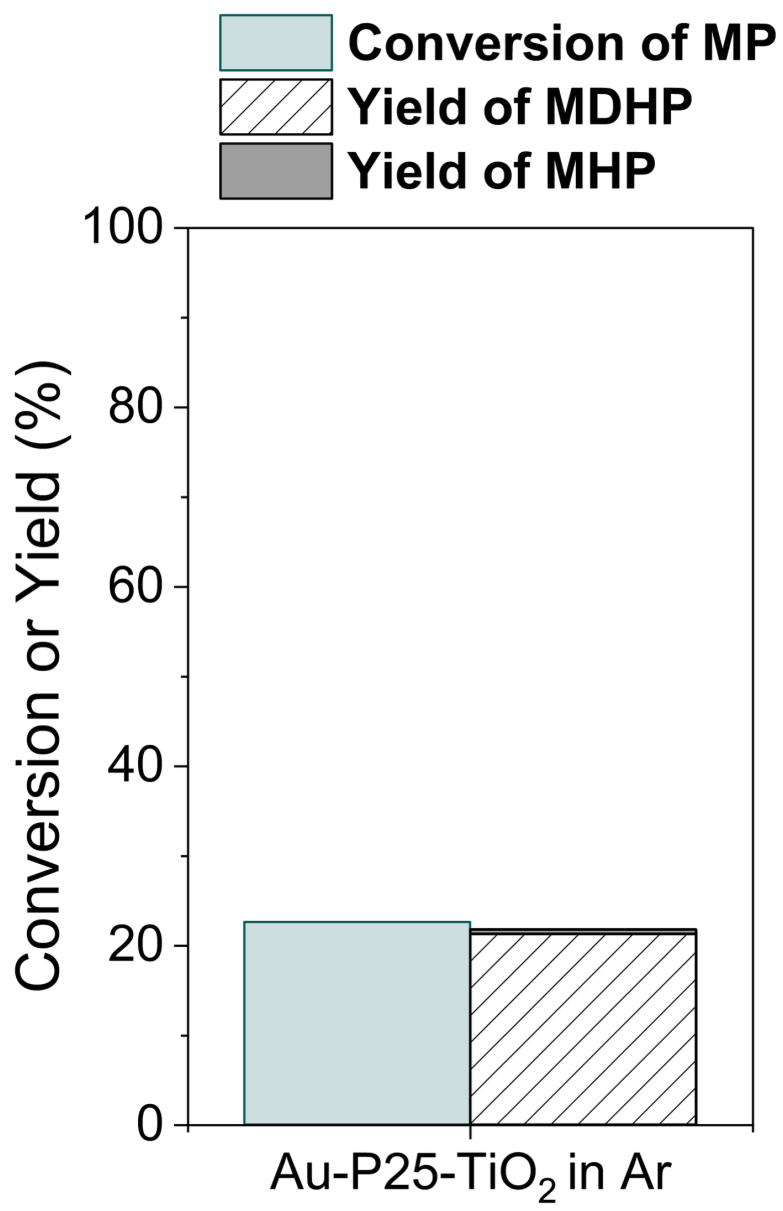

Figure S28. Conversion and yields of Au-P25-TiO_2 run in argon, under conditions similar to those in Figure S23.

References

(1) Klayman, D. L.; Woods, T. S. 2-Amino-2-Thiazoline. Unequivocal Structure Assignment of the Products of the Reaction of 2-Amino-2-Thiazoline and Its Analogs with Carbethoxy Isothiocyanate. *Journal of Organic Chemistry* **1974**, *39* (13), 1819–1823.

(2) Orekhov, N.; Kondratyuk, N.; Logunov, M.; Timralieva, A.; Shilovskikh, V.; Skorb, E. V. Insights into the Early Stages of Melamine Cyanurate Nucleation from Aqueous Solution. *Cryst. Growth Des.* **2021**, *21* (4), 1984–1992. https://doi.org/10.1021/acs.cgd.0c01285

(3) Wu, J. X.; Bag, P. P.; Xu, Y. T.; Gong, L.; He, C. T.; Chen, X. M.; Zhang, J. P. Graphene-Like Hydrogen-Bonded Melamine–Cyanuric Acid Supramolecular Nanosheets as Pseudo-Porous Catalyst Support. *Advanced Materials* **2021**, *33* (22). https://doi.org/10.1002/adma.202007368

(4) Sangeetha, V.; Kanagathara, N.; Sumathi, R.; Sivakumar, N.; Anbalagan, G. Spectral and Thermal Degradation of Melamine Cyanurate. *J. Mater.* **2013**, *2013*, 1–7. https://doi.org/10.1155/2013/262094

(5) Masud, N.; Hansal, P.; Ward, B. D.; Cable, J. Assessing the Solubility, Chemical Stability and Ecotoxicology of an Emerging Non-Halogenated Flame Retardant, Melamine Cyanurate, against a Prevalent Halogenated Congener, Tetrabromophthalic Anhydride. *Aquatic Toxicology* **2025**, *287*. https://doi.org/10.1016/j.aquatox.2025.107537

(6) Force, C.; Román, E.; Guil, J. M.; Sanz, J. XPS And1H NMR Study of Thermally Stabilized Rh/CeO2 Catalysts Submitted to Reduction/Oxidation Treatments. *Langmuir* **2007**, *23* (8), 4569–4574. https://doi.org/10.1021/la0628118

(7) Hou, Z.; Wu, K.; Wei, H.; Chi, H.; Xi, Y.; Ma, L.; Lin, X. Strong Metal-Support Interactions of TiO2 Interface-Loaded Pt Constructed under Different Atmospheres for Adjusting the Hydrogen Storage Reaction Performance of N-Ethylcarbazole. *RSC Adv.* **2024**, *14* (37), 27310–27322. https://doi.org/10.1039/d4ra03386h

(8) Li, P.; Arman, H. D.; Wang, H.; Weng, L.; Alfooty, K.; Angawi, R. F.; Chen, B. Solvent Dependent Structures of Melamine: Porous or Nonporous? *Cryst. Growth Des.* **2015**, *15* (4), 1871–1875. https://doi.org/10.1021/acs.cgd.5b00039

(9) Carrettin, S.; McMorn, P.; Johnston, P.; Griffin, K.; Kiely, C. J.; Hutchings, G. J. Oxidation of Glycerol Using Supported Pt, Pd and Au Catalysts. *Physical Chemistry Chemical Physics* **2003**, *5* (6), 1329–1336. https://doi.org/10.1039/b212047j

(10) Petrovykh, D. Y.; Kimura-Suda, H.; Opdahl, A.; Richter, L. J.; Tarlov, M. J.; Whitman, L. J. Alkanethiols on Platinum: Multicomponent Self-Assembled Monolayers. *Langmuir* **2006**, *22* (6), 2578–2587. https://doi.org/10.1021/la050928a

(11) Fu, X.; Wang, Y.; Wu, N.; Gui, L.; Tang, Y. Surface Modification of Small Platinum Nanoclusters with Alkylamine and Alkylthiol: An XPS Study on the Influence of Organic Ligands on the Pt 4f Binding Energies of Small Platinum Nanoclusters. *J. Colloid Interface Sci.* **2001**, *243* (2), 326–330. https://doi.org/10.1006/jcis.2001.7861

(12) Prior, T. J.; Armstrong, J. A.; Benoit, D. M.; Marshall, K. L. The Structure of the Melamine-Cyanuric Acid Co-Crystal. *CrystEngComm* **2013**, *15*, 5838–5843. https://doi.org/10.1039/c3ce40709h

(13) Armelao, L.; Barreca, D.; Bottaro, G.; Gasparotto, A.; Maragno, C.; Tondello, E. Study of Ag/SiO2 Nanosystems by XPS. *Surface Science Spectra* **2003**, *10*, 170–181.

(14) Xia, K.; Yatabe, T.; Yonesato, K.; Yabe, T.; Kikkawa, S.; Yamazoe, S.; Nakata, A.; Yamaguchi, K.; Suzuki, K. Supported Anionic Gold Nanoparticle Catalysts Modified Using Highly Negatively Charged Multivacant Polyoxometalates. *Angew. Chem. Int. Ed.* **2022**. https://doi.org/10.1002/anie.202205873

(15) Chen, H.; Yang, Y.; Wang, L.; Niu, Y.; Guo, M.; Ren, X.; Zhao, W.; Tang, X.; Wang, G. Slicing and Splicing of Bromodifluoro-N-Arylacetamides: Dearomatization and Difunctionalization of Pyridines. *Org. Lett.* **2020**, *22* (16), 6610–6616. https://doi.org/10.1021/acs.orglett.0c02368

(16) Shekhar, M.; Wang, J.; Lee, W. S.; Williams, W. D.; Kim, S. M.; Stach, E. A.; Miller, J. T.; Delgass, W. N.; Ribeiro, F. H. Size and Support Effects for the Water-Gas Shift Catalysis over Gold Nanoparticles Supported on Model Al 2O 3 and TiO 2. *J. Am. Chem. Soc.* **2012**, *134* (10), 4700–4708. https://doi.org/10.1021/ja210083d

(17) Chen, J.; Fang, W.; Zhang, Q.; Deng, W.; Wang, Y. A Comparative Study of Size Effects in the Au-Catalyzed Oxidative and Non-Oxidative Dehydrogenation of Benzyl Alcohol. *Chem. Asian J.* **2014**, *9* (8), 2187–2196. https://doi.org/10.1002/asia.201402238
